# Drug Efficacy in the Treatment of Antipsychotic-Induced Akathisia: A Systematic Review and Network Meta-Analysis

**DOI:** 10.1001/jamanetworkopen.2024.1527

**Published:** 2024-03-07

**Authors:** Cyril Gerolymos, Romain Barazer, Dong Keon Yon, Anderson Loundou, Laurent Boyer, Guillaume Fond

**Affiliations:** 1 Health Service Research and Quality of Life Center (CEReSS), Assistance Publique-Hôpitaux de Marseille, Aix-Marseille Université, Marseille, France; 2 FondaMental Foundation, Creteil, France; 3 Department of Pediatrics, Kyung Hee University College of Medicine, Seoul, Republic of Korea; 4 Center for Digital Health, Medical Science Research Institute, Kyung Hee University Medical Center, Kyung Hee University College of Medicine, Seoul, Republic of Korea

## Abstract

**Importance:**

Antipsychotic-induced akathisia (AIA) occurs in 14% to 35% of patients treated with antipsychotics and is associated with increased suicide and decreased adherence in patients with schizophrenia. However, no comprehensive review and network meta-analysis has been conducted to compare the efficacy of treatments for AIA.

**Objective:**

To compare the efficacy associated with AIA treatments.

**Data Sources:**

Three databases (MEDLINE, Web of Science, and Google Scholar) were systematically searched by multiple researchers for double-blind randomized clinical trials (RCTs) comparing active drugs for the treatment of AIA with placebo or another treatment between May 30 and June 18, 2023.

**Study Selection:**

Selected studies were RCTs that compared adjunctive drugs for AIA vs placebo or adjunctive treatment in patients treated with antipsychotics fulfilling the criteria for akathisia, RCTs with sample size of 10 patients or more, only trials in which no additional drugs were administered during the study, and RCTs that used a validated akathisia score. Trials with missing data for the main outcome (akathisia score at the end points) were excluded.

**Data Extraction and Synthesis:**

Data extraction and synthesis were performed, estimating standardized mean differences (SMDs) through pairwise and network meta-analysis with a random-effects model. The Preferred Reporting Items for Systematic Reviews and Meta-Analyses (PRISMA) guideline was followed.

**Main Outcomes and Measures:**

The primary outcome was the severity of akathisia measured by a validated scale at the last available end point.

**Results:**

Fifteen trials involving 492 participants compared 10 treatments with placebo. Mirtazapine (15 mg/d for ≥5 days; SMD, −1.20; 95% CI, −1.83 to −0.58), biperiden (6 mg/d for ≥14 days; SMD, −1.01; 95% CI, −1.69 to −0.34), vitamin B_6_ (600-1200 mg/d for ≥5 days; SMD, −0.92; 95% CI, −1.57 to −0.26), trazodone (50 mg/d for ≥5 days; SMD, −0.84; 95% CI, −1.54 to −0.14), mianserin (15 mg/d for ≥5 days; SMD, −0.81; 95% CI, −1.44 to −0.19), and propranolol (20 mg/d for ≥6 days; SMD, −0.78; 95% CI, −1.35 to −0.22) were associated with greater efficacy than placebo, with low to moderate heterogeneity (*I*^2^ = 34.6%; 95% CI, 0.0%-71.1%). Cyproheptadine, clonazepam, zolmitriptan, and valproate did not yield significant effects. Eight trials were rated as having low risk of bias; 2, moderate risk; and 5, high risk. Sensitivity analyses generally confirmed the results for all drugs except for cyproheptadine and propranolol. No association between effect sizes and psychotic severity was found.

**Conclusions and Relevance:**

In this systematic review and network meta-analysis, mirtazapine, biperiden, and vitamin B_6_ were associated with the greatest efficacy for AIA, with vitamin B_6_ having the best efficacy and tolerance profile. Trazodone, mianserin, and propranolol appeared as effective alternatives with slightly less favorable efficacy and tolerance profiles. These findings should assist prescribers in selecting an appropriate medication for treating AIA.

## Key Points

**Question:**

Which drugs are associated with the greatest efficacy in the treatment of antipsychotic-induced akathisia?

**Findings:**

This systematic review and network meta-analysis assessed the global akathisia score of 10 drugs in 15 double-blind randomized clinical trials with 492 participants. Mirtazapine, vitamin B_6_, and biperiden exhibited the 3 most favorable efficacy and tolerability profiles; trazodone, mianserin, and propranolol demonstrated greater efficacy than the placebo; and cyproheptadine, clonazepam, zolmitriptan, and valproate did not yield significant effects.

**Meaning:**

Vitamin B_6_ may have the most favorable efficacy and tolerability profile, followed by mirtazapine and biperiden, for the treatment of antipsychotic-induced akathisia.

## Teaser

This systematic review with network meta-analysis compares the efficacy associated with 10 drugs for the treatment of antipsychotic-induced akathisia.

## Introduction

The *Diagnostic and Statistical Manual of Mental Disorders, Fifth Edition* (*DSM-5*) defines akathisia as “subjective complaints of restlessness, often accompanied by objective excessive movements (such as continuous leg movements, rocking from foot to foot, pacing, or an inability to sit down and remain still).”^1^ This movement disorder primarily occurs in patients treated with antipsychotics, where it is known as antipsychotic-induced akathisia (AIA). A meta-analysis^2^ published in 2019 that compared the tolerability of 32 antipsychotics concluded that first-generation antipsychotics carry a higher risk of inducing akathisia compared with second-generation antipsychotics, with risks ranging from 24-fold (zuclopenthixol, a first-generation antipsychotic) to 1.9-fold (aripiprazole, a second-generation antipsychotic). Overall, the prevalence of AIA ranges from 14% to 35% based on studies involving patients treated with antipsychotics.^3,4,5,6,7^ This adverse effect has significant clinical implications, including an increased risk of suicide (which is the primary cause of mortality in early schizophrenia) and nonadherence to treatment (which is the primary cause of relapse).^8^

The primary clinical recommendations for treating AIA are to consider antipsychotic monotherapy, to reduce the antipsychotic dose, and/or to switch to an antipsychotic associated with a lower risk of akathisia.^9^ However, these options are not always feasible in clinical practice, and addressing akathisia remains challenging in many cases. Therefore, the use of adjunctive drugs is necessary to alleviate AIA. Because of the lack of comprehensive network meta-analyses that examine the efficacy of treatments in alleviating akathisia, we performed a meta-analysis to examine the efficacy of drugs in treating AIA. A secondary objective was to assess the acceptability (defined as the proportion of dropout due to tolerance issues) and tolerability (defined by the frequency of adverse events) of each drug.

## Methods

This study follows the 2020 Preferred Reporting Items for Systematic Reviews and Meta-analyses (PRISMA) reporting guideline.^10^ The protocol has been registered in PROSPERO (CRD42023431136) without any amendments to the provided information.

### Study Eligibility Criteria

Two authors (R.B. and G.F.) searched 3 databases (MEDLINE, Web of Science, and Google Scholar) from inception, with no language restriction. In case of no consensus on the inclusion of a study, a third author (L.B.) made the final decision about study inclusion. The systematic review began on May 30, 2023, and ended on June 18, 2023. The search terms were as follows: *akathisia* AND *antipsychotic* OR *neuroleptic* OR *schizophrenia* OR *schizoaffective disorder* combined with a list of the different adjunctive drugs. A second search was performed for each drug identified in the first search: *biperiden, clonazepam, cyproheptadine, diazepam, diphenhydramine, mianserin, mirtazapine, propranolol, trazodone, valproate, vitamin B_6_,* and *zolmitriptan.* Each drug term was combined with the term *akathisia*. The search identified 847 records.

Inclusion criteria were as follows: randomized clinical trials (RCTs) that (1) compared an adjunctive drug for AIA vs placebo or adjunctive treatment in patients treated with antipsychotics fulfilling the criteria for akathisia, (2) had sample sizes of at least 10 patients, (3) had no additional drugs administered during the study, and (4) used a validated akathisia score. Trials with missing data for the main outcome (akathisia score at the 2 end points) were excluded.

### Outcomes

Our primary outcome was the reduction of the mean akathisia score on the last time point with a scale assessing global, subjective, and/or objective akathisia. When repeated assessment time points were recorded, we chose the longest one. For crossover trials, the last scores at the end of the first period were analyzed. Secondary outcomes were tolerance, defined as the total number of adverse effects and the total number of serious adverse effects reported at the end of the trial, and acceptability, defined as the number of dropouts after randomization for tolerance issues (if the reasons for dropout were not available, the total number of dropouts was included).

### Data Extraction

All records were screened and extracted by 2 researchers (R.B. and C.G.). Disagreements were resolved through discussion with a third author (G.F.). The secondary outcomes were extracted through the trials, namely, the total number of patients experiencing adverse effects and dropouts. Percentages relative to the total number of participants in the network meta-analysis are presented. Twenty-seven baseline characteristics were extracted and are presented in the eMethods in Supplement 1.

### Risk of Bias

We assessed the risk of bias for individual studies according to the *Cochrane Handbook for Systematic Reviews of Interventions* using the Risk of Bias 2 tool.^11^ More details on identification and selection of studies, outcomes, data extraction, missing data, risk of bias, and the details of statistical analyses are presented in the eMethods in Supplement 1.

### Statistical Analysis

We estimated standardized mean differences (SMDs) and their respective SEs for continuous outcome using pairwise and network meta-analysis. If the total sample size was 20 or fewer patients, a Hedges g (SMD) correction was applied.^12^ If not, a Cohen *d* (SMD) was calculated. The different effect sizes were compiled using a frequentist random-effects network meta-analysis model, and 95% CIs are presented. Pooled weights for each intervention were calculated from pairwise comparisons model.^13^ Results were resumed in a forest plot and a league table.

The statistical heterogeneity of our model was assessed with *I*^2^ and τ^2^.^14,15^ A Cochran *Q* test^16^ was conducted for overall heterogeneity and inconsistency. The ranking of treatments is presented through P-score based on the random-effects model. Surface under the cumulative ranking curve (SUCRA) has been used for the rankogram. To examine the transitivity assumption, we listed relevant sociodemographic and clinical factors and compared them by means of boxplots. Tests and 2-sided *P* value threshold for statistical significance were as follows: Cochran *Q* test *P* < .05, Separate Indirect From Direct Evidence (SIDE) test *P* < .10, and Egger test, Pustejovsky-Rodgers corrected test, and Thompson-Sharp test *P* < .05. All statistical analyses were performed using the netmeta package (version 2.8-2) in R software, version 4.1.3 (R Project for Statistical Computing).^17^

## Results

### Selection, Inclusion, and Characteristics of Studies

**Figure 1.**
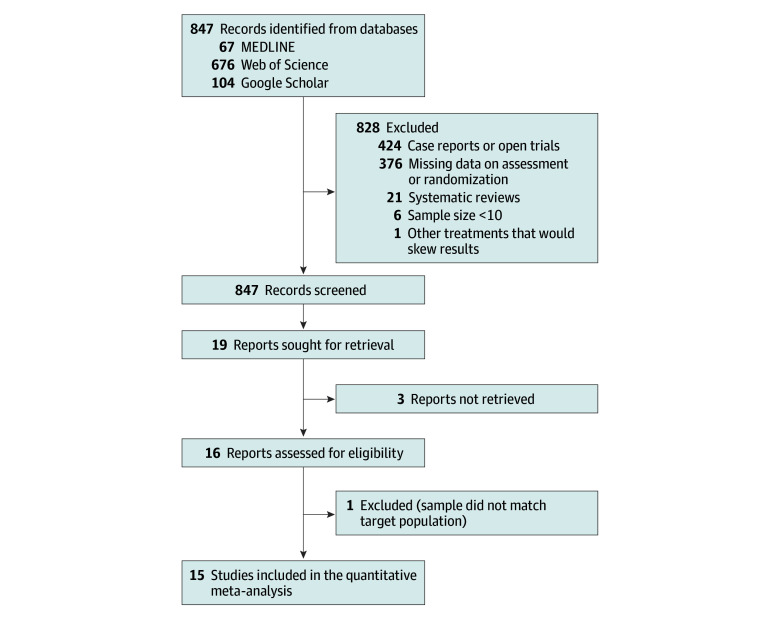
Study Selection Process Overall, 15 double-blind randomized clinical trials corresponding to 12 interventions were included.

Figure 1 illustrates the flowchart of the study analysis. Of the 847 records identified initially in the databases, we selected 15 double-blind RCTs (1.8%) that met the inclusion criteria for a network meta-analysis.^18,19,20,21,22,23,24,25,26,27,28,29,30,31,32^ The excluded studies and reasons for exclusion are presented in eTable 1 in Supplement 1.

The 15 included RCTs consist of 10 parallel group trials (66.7%),^19,20,21,22,26,27,28,29,30,32^3 crossover trials (20.0%),^18,24,25^ and 3 multiarm studies (20.0%)^23,25,31^ (1 trial has both crossover and multiarm designs). A total of 492 patients are included, with 324 patients (65.9%) allocated to the experimental arms and 168 patients (34.1%) to the placebo arm. The placebo acted as the reference group in 12 studies (80.0%),^18,19,21,23,24,25,26,27,28,29,30,31^ whereas in 3 studies (20.0%),^20,22,32^ an active treatment served as the reference group (2 studies used propranolol, and 1 study used diphenhydramine). The RCTs used 5 scores: Barnes Akathisia Rating Scale, Akathisia Rating Scale, Simpson Angus Scale, and 2 scores based on the *DSM-5* criteria for akathisia. The descriptions of these scales and the characteristics of the trials can be found in eTable 2 in Supplement 1.

**Figure 2.**
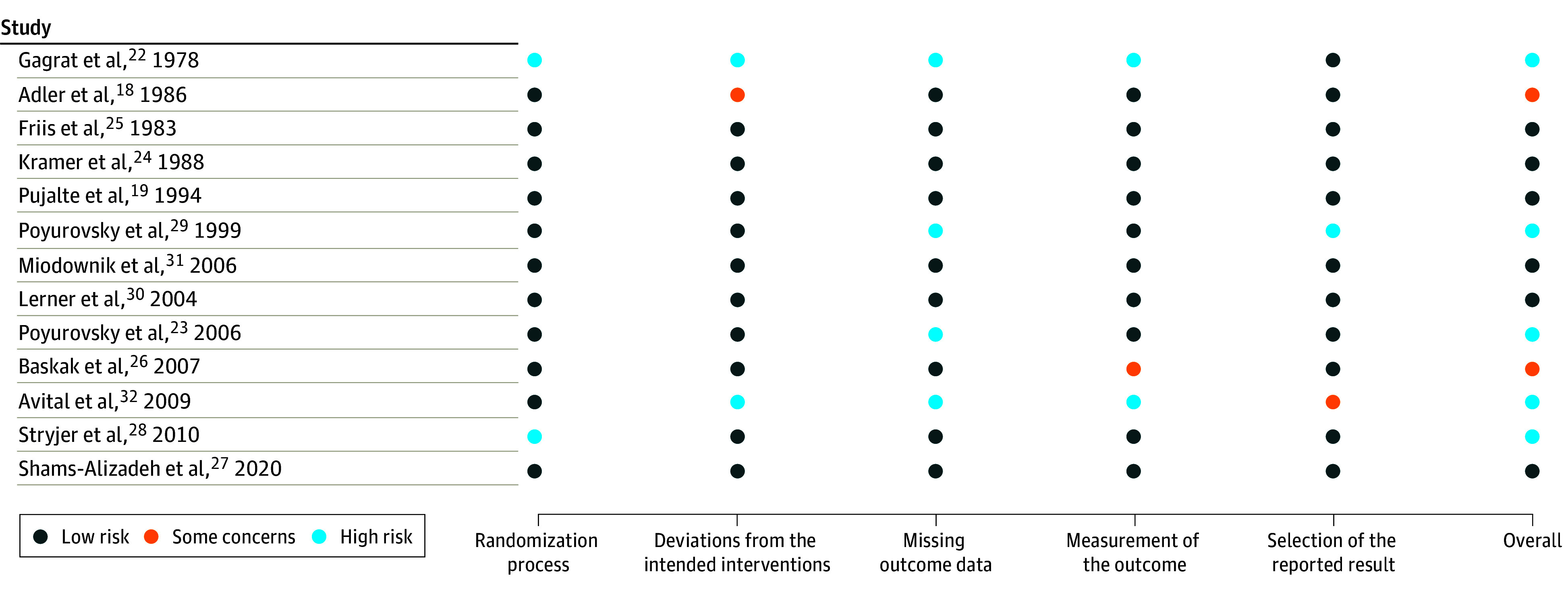
Risk of Bias Assessment for the 15 Studies Included in the Network Meta-Analysis

Regarding the risk of bias assessment, 13 studies (86.7%)^18,19,20,21,23,24,25,26,27,28,29,30,31,32^ reported an adequate randomization process, 12 studies (80.0%)^19,20,21,23,24,25,26,27,28,29,30,31^ did not present any deviations from intended interventions, 11 studies (73.3%)^18,19,20,21,24,25,26,27,28,30,31^ dealt properly with missing outcome data, 12 studies (80.0%)^18,19,20,21,24,25,26,27,28,30,31^ used an appropriate way to measure outcome, and 13 studies (86.7%)^18,19,20,21,22,23,24,25,26,27,28,30,31^ reported the full results without any selection. Eight studies (53.3%)^19,20,21,24,25,27,30,31^ were at overall low risk of bias and met all the quality criteria, 12 studies (80.0%)^18,19,20,21,23,24,25,26,27,28,30,31^ met at least 4 quality criteria, and 13 studies (86.7%)^18,19,20,21,23,24,25,26,27,28,29,30,31^ met at least 3 quality criteria. Two studies (13.3%)^18,26^ presented some concerns, whereas 5 studies (33.3%)^22,23,28,29,32^ were categorized as high risk of bias. Figure 2 provides details of the risk of bias assessment.

### Network Graph

**Figure 3.**
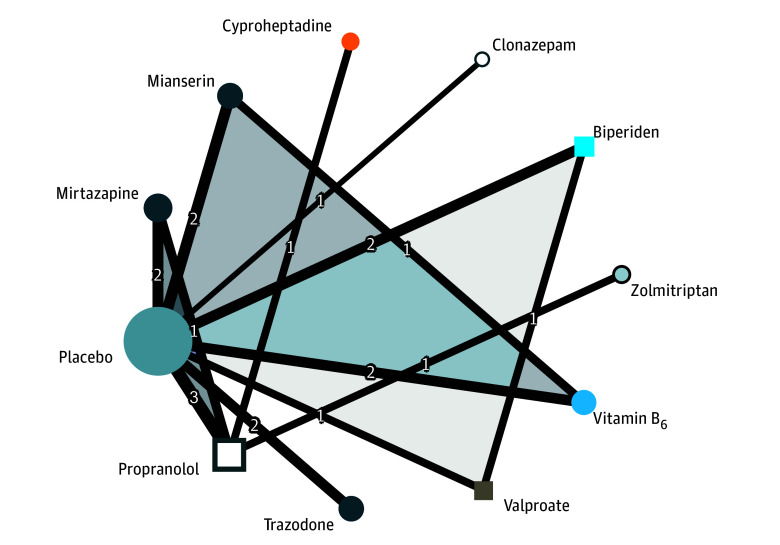
Network Graph for Main Results of Meta-Analysis Node size is proportional to the total number of patients for each intervention. Line thickness is proportional to weight from the random-effects model. The number overlying the lines is equal to the number of studies corresponding to the comparison. Triangles represent comparisons for 3-arm studies. Colors of the nodes represent the therapeutic classes of intervention: β-blocker (propranolol), antidepressant (mirtazapine, mianserin, and trazodone), mood stabilizer (valproate), anticholinergic (biperiden), antihistaminic (cyproheptadine), benzodiazepine (clonazepam), and triptan (zolmitriptan).

The network graph is presented in Figure 3. One study^22^ was excluded from the main network due to the absence of a comparison linking diazepam or diphenhydramine with other treatments or placebo in the network, leaving 14 trials in the main network. Individual effect size and calculation for the study by Gagrat et al^22^ are described in eTable 3 in Supplement 1. Despite the limited number of trials, the network demonstrated overall strong connectivity. The most frequently studied comparisons involved propranolol, mirtazapine, mianserin, vitamin B_6_, biperiden, and the placebo control condition. Cyproheptadine, clonazepam, and zolmitriptan had weak connections within the network, with only 1 direct comparison each.

eTable 4 in Supplement 1 summarizes the characteristics of the interventions initially included in the network. In descending order, the placebo, propranolol, mirtazapine, mianserin, vitamin B_6_, and biperiden arms account for the highest number of participants.

### Network Meta-Analysis for Efficacy

**Figure 4.**
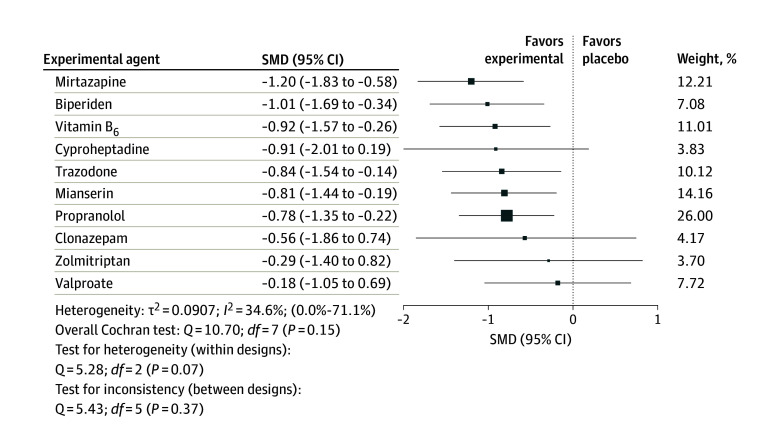
Ranked Forest Plot of Network Meta-Analysis for Efficacy of Treatments in Antipsychotic-Induced Akathisia The different treatments are compared with placebo (the reference group). The more negative the standardized mean difference (SMD) is, the higher the reduction in akathisia scale score for intervention compared with placebo. Weight represents the sum of the inverse variance of all effect sizes in the pairwise comparison model for respective intervention. Square size is proportional to weight.

Figure 4 illustrates that the following medications were associated with significantly greater efficacy than placebo in the treatment of AIA: mirtazapine (15 mg/d for ≥5 days; SMD, −1.20; 95% CI, −1.83 to −0.58), biperiden (6 mg/d for ≥14 days; SMD, −1.01; 95% CI, −1.69 to −0.34), vitamin B_6_ (600-1200 mg/d for ≥5 days; SMD, −0.92; 95% CI, −1.57 to −0.26), trazodone (50 mg/d for ≥5 days; SMD, −0.84; 95% CI, −1.54 to −0.14), mianserin (15 mg/d for ≥5 days; SMD, −0.81; 95% CI, −1.44 to −0.19), and propranolol (20 mg/d for ≥6 days; SMD, −0.78; 95% CI, −1.35 to −0.22) were associated with greater efficacy than placebo, with low to moderate heterogeneity (*I*^2^ = 34.6%; 95% CI, 0.0%-71.1%). However, cyproheptadine (16 mg/d), clonazepam (0.5-2.5 mg/d), zolmitriptan (7.5 mg/d), and valproate (1700 mg/d) did not show a significant difference compared with placebo. The SMDs ranged from −1.20 (95% CI, −1.83 to −0.58) for mirtazapine to −0.18 (95% CI, −1.05 to 0.69) for valproate. The between-study heterogeneity was estimated at τ^2^ = 0.0907 (95% CI, 0.0-0.30).

**Figure 5.**
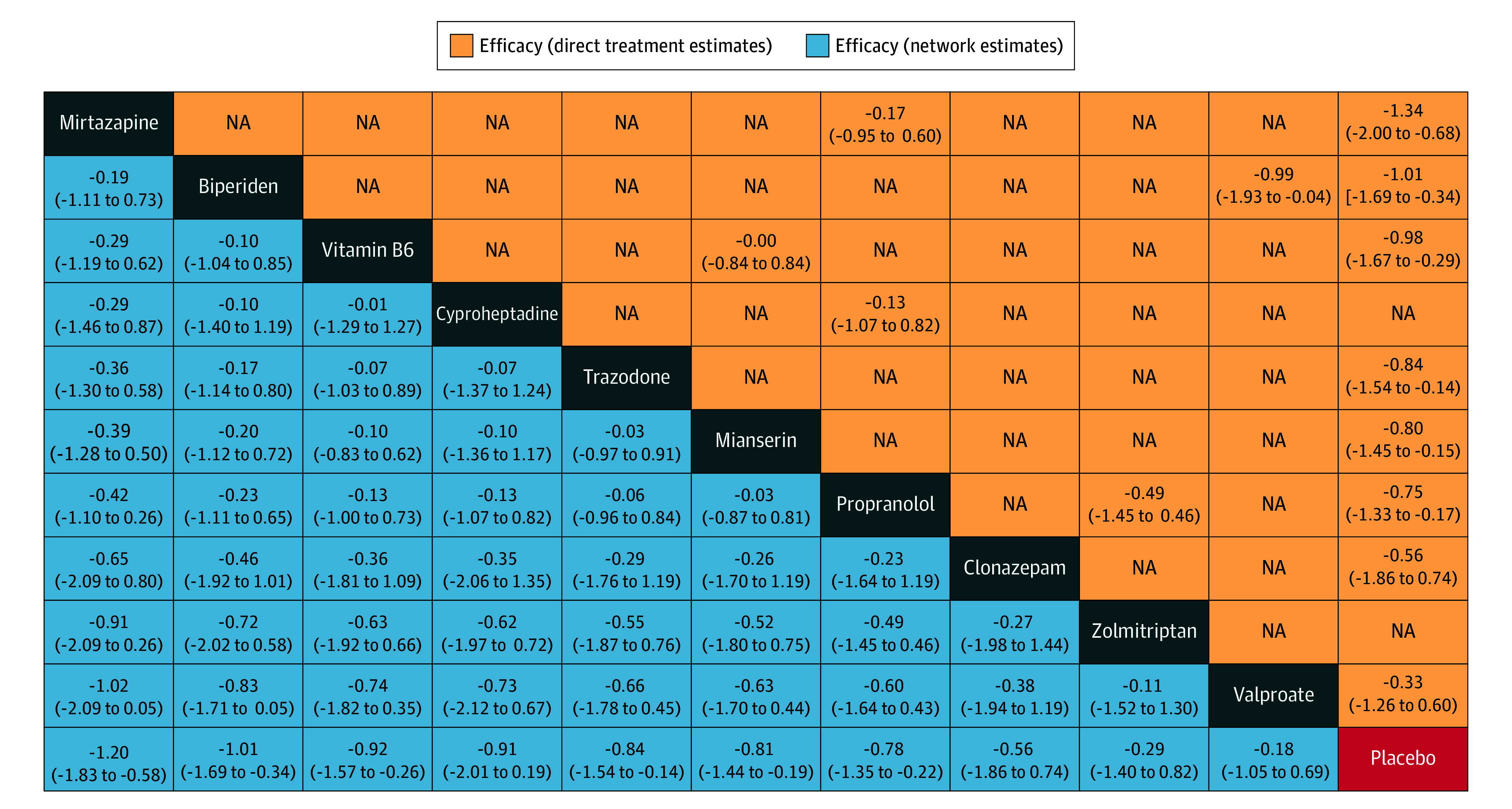
Head-to-Head Comparisons for Efficacy of the 10 Treatments in Antipsychotic-Induced Akathisia Drugs are reported by decreasing rank order. Data are standardized mean differences (SMDs) (95% CIs). Comparisons should be read from left to right. For the lower triangle that reports network estimates, column-defining treatment is compared with row-defining treatment. For the upper triangle that reports direct treatment estimates, row-defining treatment is compared with column-defining treatment. The SMDs above 0 favor the column-defining treatment in the lower triangle, whereas they favor the row-defining treatment in the upper triangle. NA indicates not applicable.

In Figure 5, the head-to-head comparisons of the efficacy of the 10 included treatments and placebo are presented in order of ranking. None of the network estimates comparing active treatments reached statistical significance (all 95% CIs of SMDs contain the value 0). There was no direct evidence comparing cyproheptadine or zolmitriptan with placebo because the trials evaluating these drugs did not include placebo as a reference group. Only biperiden demonstrated greater efficacy than valproate (SMD, −0.99; 95% CI, −1.93 to −0.04). There was no significant difference in efficacy between propranolol and zolmitriptan, cyproheptadine and propranolol, mirtazapine and propranolol, or vitamin B_6_ and mianserin.

eFigure 2 in Supplement 1 provides a summary of the ranking using SUCRA. The top 5 ranked molecules for treating AIA, in decreasing order of probability, are mirtazapine, biperiden, vitamin B_6_, cyproheptadine, and trazodone. Mirtazapine had a 33.4% probability of being the top-ranked molecule, whereas propranolol had a 16.4% probability of being ranked seventh (eTable 5 in Supplement 1). The P-scores support these findings, ranking mirtazapine (P-score = 0.826), biperiden (P-score = 0.703), and vitamin B_6_ (P-score = 0.641) as the 3 most effective therapies for treating AIA (eFigure 1 in Supplement 1). The results for the efficacy at different time points and the efficacy on subjective and objective symptoms are presented in the eResults in Supplement 1.

### Consistency of the Network and Reporting Bias

We did not find any evidence of inconsistency regarding the SIDE test. None of the 8 comparisons between direct and indirect evidence reached significance (eTable 6 in Supplement 1). The forest plot, which separates direct and indirect evidence (eFigure 3 in Supplement 1), revealed that the network comparison with the most influence on heterogeneity (73%) was propranolol vs placebo. The proportion of direct and indirect comparisons for each face-to-face intervention is presented in eFigure 4 in Supplement 1.

Funnel plot analysis did not indicate any skewness (eFigure 5 in Supplement 1). The statistical significance of the Egger test was not observed. The Pustejovsky-Rodgers corrected test for continuous effect sizes confirmed this trend along with the Thompson-Sharp test, supporting the absence of reporting bias (eTables 7-9 in Supplement 1).

### Acceptability and Tolerability

Details of the tolerability outcomes are reported in eTable 10 in Supplement 1. Significant adverse effects reported compared with the placebo arm included drowsiness and dizziness for trazodone and mirtazapine, hypersalivation and depression for valproate, dry mouth and sedation for biperiden and valproate, hypotension for propranolol, and transient sedation for mianserin. Details of the acceptability outcomes are given in eTable 11 in Supplement 1.

### Transitivity Assumption

All details of the exploratory analysis of the transitivity assumption are summarized in the eResults in Supplement 1 and detailed in eFigures 6 to 29 in Supplement 1. Except for some outliers, the transitivity assumption was roughly met.

### Sensitivity Analyses

All details regarding the effect size modifiers and subgroup analyses are presented in eFigures 30 to 57 in Supplement 1. In all subgroup analyses, mirtazapine was ranked first, and biperiden remained in the second position for the first 2 subgroup analyses.

## Discussion

This work represents the first network meta-analysis, to our knowledge, to explore the efficacy associated with adjunctive drugs in AIA, which is a common clinical issue in psychiatric daily practice. The following adjunctive drugs demonstrated efficacy, listed in decreasing order of effect sizes: mirtazapine (15 mg/d), biperiden (2.5-15 mg/d), vitamin B_6_ (600 mg/d), mianserin (15 mg/d), trazodone (100 mg/d), and propranolol (20-120 mg/d). Cyproheptadine also appeared to be potentially effective, but data were insufficient to fully support its efficacy. Clonazepam and valproate did not demonstrate superiority over placebo and are not currently recommended. These results have important implications for clinical practice.

Mirtazapine, biperiden, and vitamin B_6_ exhibited moderate to large effect sizes with comparable efficacy, and mirtazapine consistently ranked first in both the main analysis and all subgroup analyses. However, mirtazapine may be poorly tolerated due to its sedative effects and the potential for weight gain. Mianserin is also effective, similar to mirtazapine, in reducing akathisia symptoms. This antidepressant also has a good tolerability profile except for sedation. However, 10% to 20% of patients do not respond to mirtazapine and mianserin, which suggests that other mechanisms, apart from serotonin blockade, may be involved in AIA.^31^

Vitamin B_6_ may be considered the best option in terms of the risk-benefit ratio for AIA treatment. In cases involving insomnia, mirtazapine may still be the preferred choice^33^ for the treatment of comorbid depressive disorder.^34^ Vitamin B_6_ has a moderate to large effect size that also extends to different subgroup analyses. Its major potency lies in its excellent tolerability and acceptability profile. Vitamin B_6_ may play a role, notably as a corrector of dopamine imbalance and a free radical scavenger.^30,31^

Biperiden may be the best alternative in the event of vitamin B_6_ and mirtazapine failure. Its anticholinergic action has been tested for both oral and intramuscular administration.^25,26^ However, sedation occurred in 48% of cases. The effect size of intramuscular biperiden may have been underestimated, as 1 study evaluated efficacy only 6 hours after administration,^26^ whereas its half-life varies from 11 to 24 hours.^35^ Furthermore, biperiden has been administered at a low dose (2.5-mg injections), which may have favored tolerance over efficacy. Higher doses and longer evaluation may provide better efficacy. The optimal dosage for biperiden appears to be 12 mg/d, and the optimal treatment duration is 14 days.

Trazodone is another antidepressant that has shown a significant effect. The optimal dose appears to be 100 mg/d. The most common adverse effect associated with trazodone is drowsiness. A limitation to its use is the absence of marketing authorization in some European countries, including France and Denmark. Rare cases of priapism have also been reported in men treated with trazodone.^36,37^ Additionally, trazodone should be avoided in men who have specific hematologic or neurologic diseases (such as sickle cell anemia, multiple myeloma, leukemia, hypercoagulable states, or autonomic nervous system disorders) or in men with anatomical deformations of the penis.^38^

Propranolol, 50 mg/d for 8 days, is the intervention that contributes the most to the heterogeneity of our main random-effects model due to the inclusion of trials lasting 2 days or less. It seems therefore recommended to administer propranolol for AIA with a duration longer than 2 days.^18,20,23,32^ There is no evidence to suggest that increasing the dosage beyond 50 mg/d enhances efficacy. Dumon et al^39^ compared the effects of betaxolol, a selective β-blocker, with those of propranolol in akathisia and concluded that betaxolol and propranolol demonstrate comparable efficacy. The shared mechanism of action between the 2 drugs is the blockade of β_1_-adrenergic receptors.

Studies on cyproheptadine have produced conflicting results. The SUCRA ranking places it in fourth position, but the CI of its SMD includes the value of 0, suggesting that cyproheptadine is not significantly more effective than placebo. Considering the low confidence associated with the SMD of cyproheptadine, it is important to prioritize these results over the questionable ranking. Rankings are based on probabilities and do not guarantee reliable results for wide or nonsignificant CIs.^40^ The fourth subgroup analysis, pooling by class antihistaminics (cyproheptadine and diphenhydramine), has shown similar conclusions for the efficacy of antihistaminics. Although cyproheptadine appears to be as effective as propranolol,^20^ it cannot be currently recommended, and additional data are needed.

### Limitations

Limitations of the study included the subgroup analysis for low risk of bias, which should be interpreted cautiously due to the inclusion of only 8 studies, resulting in low statistical power. The efficacy of propranolol may be underestimated due to study design. In the study by Kramer et al,^24^ propranolol did not demonstrate significant efficacy at the end of the first period (2 days), whereas it showed efficacy at the end of the study (5 days). Some studies excluded benzodiazepines and anticholinergics, whereas others included them but ensured that they were initiated before the start of the trial, with constant and balanced doses between arms to avoid confounding effects. Because only 1.5% of patients were treated with antipsychotic polytherapy, the efficacy of AIA can theoretically not be extrapolated to patients treated with antipsychotic polytherapy. However, antipsychotic polytherapy is common in clinical practice and is a recognized risk factor of AIA, and antipsychotic daily dose was not associated with efficacy in our results. Antipsychotic monotherapy is still recommended in cases of akathisia induced by antipsychotic polytherapy. Additionally, we did not use pre-post effect sizes to evaluate the evolution of scores over time because the intertime correlation coefficient (*r*) was not reported in the trials. Furthermore, between-group SMDs provide better control over covariates compared with within-group SMDs.^41^ Finally, our random-effects model relies on numerous indirect evidence due to the lack of direct comparisons between active treatments. The consistency between direct and indirect evidence defends the accuracy of network estimates.

## Conclusions

This systematic review and network meta-analysis found that mirtazapine (15 mg/d for ≥5 days), biperiden (12 mg/d for ≥14 days), and vitamin B_6_ (600 mg/d for ≥5 days) were associated with the greatest efficacy for treating AIA, with vitamin B_6_ having the best efficacy and tolerance profile. Because the number of available RCTs remains low and sample sizes are limited, prudence is advised. Trazodone (100 mg/d for ≥5 days), mianserin (15 mg/d for ≥5 days), and propranolol (50 mg/d for ≥8 days) may be effective alternatives with less favorable efficacy and tolerance profiles.

## Notes

Supplement 1. **eMethods.** Identification and selection of studies, outcomes, missing data, and statistical analysis **eResults.** Results for the efficacy at different time points and the efficacy on subjective and objective symptoms **eFigure 1.** P-score ranking for the 10 interventions treating AIA and placebo **eFigure 2.** Bar chart (rankogram) showing Surface Under the Cumulative Ranking (SUCRA) score for the efficacy of the 10 different treatments in AIA and placebo **eFigure 3.** Forest plot separating direct and indirect evidence for pairwise comparisons in NMA **eFigure 4.** Plot showing direct and indirect evidence proportions for each network estimate (random-effects model) **eFigure 5.** Funnel plot of NMA showing publication bias assessment **eTable 1.** Excluded studies and reasons for exclusion **eTable 2.** Characteristics of the included studies comprised in network meta-analysis **eTable 3.** Subnetwork for Gagrat 1978 with treatment effect size estimate and calculation method **eTable 4.** Characteristics of the interventions comprised in the network meta-analysis **eTable 5.** Cross-tabulation of ranking probabilities for the 10 akathisia interventions and placebo **eTable 6.** Results for Separate Indirect from Direct Evidence (SIDE) using back-calculation method, testing agreement between direct and indirect evidence **eTable 7.** Egger's test details (publication bias assessment) **eTable 8.** Pustejovsky-Rodgers's corrected test details (for standard error of SMD) (publication bias assessment) **eTable 9.** Thompson-Sharp's test details (publication bias assessment) **eTable 10.** Tolerability outcome details: adverse events for the 10 akathisia interventions and placebo (meta-analysis level) **eTable 11.** Acceptability outcome details: dropouts for the 10 akathisia interventions and placebo (meta-analysis level) **eFigure 6.** Boxplot showing mean ratio clustered by intervention **eFigure 7.** Boxplot showing age (mean) clustered by intervention **eFigure 8.** Error bars showing mean age ± standard deviation clustered by intervention **eFigure 9.** Boxplot showing schizophrenia ratio (DSM-5 diagnosis) in participants clustered by intervention **eFigure 10.** Boxplot showing akathisia treatment duration (days) clustered by intervention **eFigure 11.** Boxplot showing akathisia treatment dose (mg/day), sort by dose range (A – low dose) for each intervention **eFigure 12.** Boxplots showing akathisia treatment dose (mg/day), sort by dose range (B – intermediate dose, C – high) for each intervention **eFigure 13.** Boxplot showing PANSS severity score at baseline (means) for each intervention **eFigure 14.** Error bars showing the mean PANSS scores at baseline ± standard deviation for each intervention **eFigure 15.** Boxplot showing first-generation (typical) antipsychotic (FGA) ratio for each intervention **eFigure 16.** Boxplot showing chlorpromazine equivalent dose (mg/day) for each intervention **eFigure 17.** Error bars showing mean chlorpromazine equivalent dose (mg/day) ± standard deviation for each intervention **eFigure 18.** Boxplot showing ratio of patients treated with anticholinergic as cotreatment for each intervention **eFigure 19.** Error bars showing mean biperiden dose (cotreatment drug) (mg/day) ± standard deviation for each intervention **eFigure 20.** Error bars showing mean trihexyphenidyl dose (mg/day) ± standard deviation for each intervention **eFigure 21.** Error bars showing mean benztropine dose (A), procyclidine dose (B) and tropatepine dose (C) (mg/day) ± standard deviation for each intervention **eFigure 22.** Boxplot showing ratio of patients treated with benzodiazepine (cotreatment drug) for each intervention **eFigure 23.** Error bars showing mean diazepam dose (cotreatment drug) (mg/day) ± standard deviation for each intervention **eFigure 24.** Error bars showing mean lorazepam dose (mg/day) ± standard deviation for each intervention **eFigure 25.** Error bars showing mean nitrazepam dose (A), clonazepam dose (B) (cotreatment drug) and temazepam dose (C) (mg/day) ± standard deviation for each intervention **eFigure 26.** Boxplot showing mean Simpson Angus Scale (SAS) scores at baseline for each intervention **eFigure 27.** Error bars showing mean SAS scores at baseline ± standard deviation for each intervention **eFigure 28.** Boxplot showing mean Hamilton Depression Rating Scale (HAM-D) scores at baseline for each intervention **eFigure 29.** Error bars showing mean HAM-D scores at baseline ± standard deviation for each intervention **eFigure 30.** Error bars showing the different standardized mean differences ± standard deviation for each intervention according to akathisia treatment duration (in days) **eFigure 31.** Error bars showing the different standardized mean differences ± standard deviation for each intervention, sort by dose range (A – low dose), according to akathisia treatment dose (mg/day) **eFigure 32.** Error bars showing the different standardized mean differences ± standard deviation for each intervention, sort by dose range (B – intermediate dose, C – high dose), according to akathisia treatment dose (mg/day) **eFigure 33.** Error bars showing the different standardized mean differences ± standard deviation for each intervention according to PANSS score (mean) **eFigure 34.** Error bars showing the different standardized mean differences ± standard deviation for each intervention according to chlorpromazine equivalent dose (mg/day) **eFigure 35.** Error bars showing the different standardized mean differences ± standard deviation for each intervention according to biperiden mean dose (mg/day), used as cotreatment drug **eFigure 36.** Error bars showing the different standardized mean differences ± standard deviation for each intervention according to trihexyphenidyl mean dose (mg/day) **eFigure 37.** Error bars showing the different standardized mean differences ± standard deviation for each intervention according to benztropine mean dose (mg/day) **eFigure 38.** Error bars showing the different standardized mean differences ± standard deviation for each intervention according to diazepam mean dose (mg/day), used as cotreatment drug **eFigure 39.** Error bars showing the different standardized mean differences ± standard deviation for each intervention according to lorazepam mean dose (mg/day) **eFigure 40.** Error bars showing the different standardized mean differences ± standard deviation for each intervention according to nitrazepam mean dose (mg/day) **eFigure 41.** Error bars showing the different standardized mean differences ± standard deviation fo**r** each intervention according to Simpson Angus Scale score (mean) **eFigure 42.** Error bars showing the different standardized mean differences ± standard deviation for each intervention according to Hamilton Depression Rating Scale (mean) **eFigure 43.** Low-risk bias assessment, subgroup 1: network graph (random-effects model) **eFigure 44.** Low-risk bias assessment, subgroup 1: forest plot (random-effects model) **eFigure 45.** Low-risk bias assessment, subgroup 1: league table (random-effects model) **eFigure 46.** Akathisia treatment duration > two days, subgroup 2: network graph (random-effects model) **eFigure 47.** Akathisia treatment duration > two days, subgroup 2: forest plot (random-effects model) **eFigure 48.** Akathisia treatment duration > two days, subgroup 2: league table (random-effects model) **eFigure 49.** BARS as akathisia scale, subgroup 3: network graph (random-effects model) **eFigure 50.** BARS as akathisia scale, subgroup 3: forest plot (random-effects model) **eFigure 51.** BARS as akathisia scale, subgroup 3: league table (random-effects model) **eFigure 52.** Benzodiazepines, antihistaminics and other actives vs placebo, subgroup 4: network graph (random-effects model) **eFigure 53.** Benzodiazepines, antihistaminics and other actives vs placebo, subgroup 4: forest plot (random-effects model) **eFigure 54.** Benzodiazepines, antihistaminics and other actives vs placebo, subgroup 4: league table (random-effects model) **eFigure 55.** Therapeutic classes in akathisia vs placebo, subgroup 5: network graph (random-effects model) **eFigure 56.** Therapeutic classes in akathisia vs placebo, subgroup 5: forest plot (random-effects model) **eFigure 57.** Therapeutic classes in akathisia vs placebo, subgroup 5: league table (random-effects model)
eReferences

Supplement 2.
Data Sharing Statement
