# Supplementary material for: Drug Efficacy in the Treatment of Antipsychotic-Induced Akathisia: A Systematic Review and Network Meta-Analysis
Source: JAMA Netw Open. 2024 Mar 7;7(3):e241527. doi: 10.1001/jamanetworkopen.2024.1527 (PMC10921255; doi:10.1001/jamanetworkopen.2024.1527)
Supplement: Supplement 1. — eMethods. Identification and selection of studies, outcomes, missing data, and statistical analysis eResults. Results for the efficacy at different time points and the efficacy on subjective and objective symptoms eFigure 1. P-score ranking for the 10 interventions treating AIA and placebo eFigure 2. Bar chart (rankogram) showing Surface Under the Cumulative Ranking (SUCRA) score for the efficacy of the 10 different treatments in AIA and placebo eFigure 3. Forest plot separating direct and indirect evidence for pairwise comparisons in NMA eFigure 4. Plot showing direct and indirect evidence proportions for each network estimate (random-effects model) eFigure 5. Funnel plot of NMA showing publication bias assessment eTable 1. Excluded studies and reasons for exclusion eTable 2. Characteristics of the included studies comprised in network meta-analysis eTable 3. Subnetwork for Gagrat 1978 with treatment effect size estimate and calculation method eTable 4. Characteristics of the interventions comprised in the network meta-analysis eTable 5. Cross-tabulation of ranking probabilities for the 10 akathisia interventions and placebo eTable 6. Results for Separate Indirect from Direct Evidence (SIDE) using back-calculation method, testing agreement between direct and indirect evidence eTable 7. Egger's test details (publication bias assessment) eTable 8. Pustejovsky-Rodgers's corrected test details (for standard error of SMD) (publication bias assessment) eTable 9. Thompson-Sharp's test details (publication bias assessment) eTable 10. Tolerability outcome details: adverse events for the 10 akathisia interventions and placebo (meta-analysis level) eTable 11. Acceptability outcome details: dropouts for the 10 akathisia interventions and placebo (meta-analysis level) eFigure 6. Boxplot showing mean ratio clustered by intervention eFigure 7. Boxplot showing age (mean) clustered by intervention eFigure 8. Error bars showing mean age ± standard deviation clustered by intervention [file jamanetwopen-e241527-s001.pdf]

Supplementary Online Content

Gerolymos C, Barazer R, Yon DK, Loundou A, Boyer L, Fond G. Drug efficacy in the treatment of akathisia: a systematic review and network meta-analysis. *JAMA Netw Open*. 2024;7(3):e241527. doi:10.1001/jamanetworkopen.2024.1527

eMethods. Identification and Selection of Studies, Outcomes, Missing Data, and Statistical Analysis
eResults. Results for the Efficacy at Different Time Points and the Efficacy on Subjective and Objective Symptoms
eFigure 1. P-Score Ranking for the 10 Interventions Treating AIA and Placebo
eFigure 2. Bar chart (rankogram) showing Surface Under the Cumulative Ranking (SUCRA) score for the efficacy of the 10 different treatments in AIA and placebo
eFigure 3. Forest plot separating direct and indirect evidence for pairwise comparisons in NMA
eFigure 4. Plot showing direct and indirect evidence proportions for each network estimate (random-effects model)
eFigure 5. Funnel plot of NMA showing publication bias assessment
eTable 1. Excluded studies and reasons for exclusion
eTable 2. Characteristics of the included studies comprised in network meta-analysis
eTable 3. Subnetwork for Gagrat 1978 with treatment effect size estimate and calculation method
eTable 4. Characteristics of the interventions comprised in the network meta-analysis
eTable 5. Cross-tabulation of ranking probabilities for the 10 akathisia interventions and placebo
eTable 6. Results for Separate indirect from direct evidence (SIDE) using back-calculation method, testing agreement between direct and indirect evidence
eTable 7. Egger's test details (publication bias assessment)
eTable 8. Pustejovsky-Rodgers's corrected test details (for standard error of SMD) (publication bias assessment)
eTable 9. Thompson-Sharp's test details (publication bias assessment)
eTable 10. Tolerability outcome details: adverse events for the 10 akathisia interventions and placebo (meta-analysis level)
eTable 11. Acceptability outcome details: dropouts for the 10 akathisia interventions and placebo (meta-analysis level)
eFigure 6. Boxplot showing men ratio clustered by intervention
eFigure 7. Boxplot showing age (mean) clustered by intervention.
eFigure 8. Error bars showing mean age ± standard deviation clustered by intervention
eFigure 9. Boxplot showing schizophrenia ratio (DSM-5 diagnosis) in participants clustered by intervention
eFigure 10. Boxplot showing akathisia treatment duration (days) clustered by intervention
eFigure 11. Boxplot showing akathisia treatment dose (mg/day), sort by dose range (A – low dose) for each intervention
eFigure 12. Boxplots showing akathisia treatment dose (mg/day), sort by dose range (B – intermediate dose, C – high) for each intervention
eFigure 13. Boxplot showing PANSS severity score at baseline (means) for each intervention

© 2024 Gerolymos C et al. *JAMA Network Open.*

eFigure 14. Error bars showing the mean PANSS scores at baseline ± standard deviation for each intervention
eFigure 15. Boxplot showing first-generation (typical) antipsychotic (FGA) ratio for each intervention
eFigure 16. Boxplot showing chlorpromazine equivalent dose (mg/day) for each intervention
eFigure 17. Error bars showing mean chlorpromazine equivalent dose (mg/day) ± standard deviation for each intervention
eFigure 18. Boxplot showing ratio of patients treated with anticholinergic as cotreatment for each intervention
eFigure 19. Error bars showing mean biperiden dose (cotreatment drug) (mg/day) ± standard deviation for each intervention
eFigure 20. Error bars showing mean trihexyphenidyl dose (mg/day) ± standard deviation for each intervention
eFigure 21. Error bars showing mean benztropine dose (A), procyclidine dose (B) and tropatepine dose (C) (mg/day) ± standard deviation for each intervention
eFigure 22. Boxplot showing ratio of patients treated with benzodiazepine (cotreatment drug) for each intervention
eFigure 23. Error bars showing mean diazepam dose (cotreatment drug) (mg/day) ± standard deviation for each intervention
eFigure 24. Error bars showing mean lorazepam dose (mg/day) ± standard deviation for each intervention
eFigure 25. Error bars showing mean nitrazepam dose (A), clonazepam dose (B) (cotreatment drug) and temazepam dose (C) (mg/day) ± standard deviation for each intervention
eFigure 26. Boxplot showing mean Simpson Angus Scale (SAS) scores at baseline for each intervention
eFigure 27. Error bars showing mean SAS scores at baseline ± standard deviation for each intervention
eFigure 28. Boxplot showing mean Hamilton Depression Rating Scale (HAM-D) scores at baseline for each intervention
eFigure 29. Error bars showing mean HAM-D scores at baseline ± standard deviation for each intervention
eFigure 30. Error bars showing the different standardized mean differences ± standard deviation for each intervention according to akathisia treatment duration (in days)
eFigure 31. Error bars showing the different standardized mean differences ± standard deviation for each intervention, sort by dose range (A – low dose), according to akathisia treatment dose (mg/day)
eFigure 32. Error bars showing the different standardized mean differences ± standard deviation for each intervention, sort by dose range (B – intermediate dose, C – high dose), according to akathisia treatment dose (mg/day)
eFigure 33. Error bars showing the different standardized mean differences ± standard deviation for each intervention according to PANSS score (mean)
eFigure 34. Error bars showing the different standardized mean differences ± standard deviation for each intervention according to chlorpromazine equivalent dose (mg/day)
eFigure 35. Error bars showing the different standardized mean differences ± standard deviation for each intervention according to biperiden mean dose (mg/day), used as cotreatment drug

© 2024 Gerolymos C et al. *JAMA Network Open.*

eFigure 36. Error bars showing the different standardized mean differences ± standard deviation for each intervention according to trihexyphenidyl mean dose (mg/day)
eFigure 37. Error bars showing the different standardized mean differences ± standard deviation for each intervention according to benztropine mean dose (mg/day)
eFigure 38. Error bars showing the different standardized mean differences ± standard deviation for each intervention according to diazepam mean dose (mg/day), used as cotreatment drug
eFigure 39. Error bars showing the different standardized mean differences ± standard deviation for each intervention according to lorazepam mean dose (mg/day)
eFigure 40. Error bars showing the different standardized mean differences ± standard deviation for each intervention according to nitrazepam mean dose (mg/day)
eFigure 41. Error bars showing the different standardized mean differences ± standard deviation for each intervention according to Simpson Angus Scale score (mean)
eFigure 42. Error bars showing the different standardized mean differences ± standard deviation for each intervention according to Hamilton Depression Rating Scale (mean)
eFigure 43. Low-risk bias assessment, subgroup 1: network graph (random-effects model)
eFigure 44. Low-risk bias assessment, subgroup 1: forest plot (random-effects model)
eFigure 45. Low-risk bias assessment, subgroup 1: league table (random-effects model)
eFigure 46. Akathisia treatment duration > two days, subgroup 2: network graph (random-effects model)
eFigure 47. Akathisia treatment duration > two days, subgroup 2: forest plot (random-effects model)
eFigure 48. Akathisia treatment duration > two days, subgroup 2: league table (random-effects model)
eFigure 49. BARS as akathisia scale, subgroup 3: network graph (random-effects model)
eFigure 50. BARS as akathisia scale, subgroup 3: forest plot (random-effects model)
eFigure 51. BARS as akathisia scale, subgroup 3: league table (random-effects model)
eFigure 52. Benzodiazepines, antihistaminics and other actives vs. placebo, subgroup 4: network graph (random-effects model)
eFigure 53. Benzodiazepines, antihistaminics and other actives vs. placebo, subgroup 4: forest plot (random-effects model)
eFigure 54. Benzodiazepines, antihistaminics and other actives vs. placebo, subgroup 4: league table (random-effects model)
eFigure 55. Therapeutic classes in akathisia vs. placebo, subgroup 5: network graph (random-effects model)
eFigure 56. Therapeutic classes in akathisia vs. placebo, subgroup 5: forest plot (random-effects model)
eFigure 57. Therapeutic classes in akathisia vs. placebo, subgroup 5: league table (random-effects model)
eReferences

This supplementary material has been provided by the authors to give readers additional information about their work.

© 2024 Gerolymos C et al. *JAMA Network Open.*

1. eMethods

Supplementary text 1. Identification and selection of studies, Outcomes, Missing data, Statistical analysis

Identification and selection of studies

The search terms used were "akathisia" AND "antipsychotic" OR "neuroleptic" OR "schizophrenia" OR “schizoaffective disorder” combined with a list of the different adjunctive drugs. A second search was carried out for each drug identified in the first search: biperiden, clonazepam, cyproheptadine, diazepam, diphenhydramine, mianserin, mirtazapine, propranolol, trazodone, valproate, vitamin B6, zolmitriptan. Each drug term was combined with "akathisia".

Outcomes

Our primary outcome is the reduction of the mean akathisia score on the last timepoint with a scale assessing global, subjective and/or objective akathisia. One important clinical issue is the under recognition of the diagnosis of AIA, especially the milder forms (Weiden et al. 1987). Furthermore, akathisia can be difficult to distinguish from psychotic anxiety or agitation. A specific scale is therefore needed to assess akathisia severity. When the akathisia severity was reported with different scales, we chose to keep BARS score if available (the most frequently used scale). When studies reported the subscales of akathisia (objective, subjective and global score), we selected global scores in the first instance and if not available, the objective scale.

Data extraction

The 27 extracted baseline characteristics were:

- mean sample age (years), percentage of men, psychiatric diagnosis,
- psychiatric severity defined by the baseline Positive and Negative Syndrome Scale (PANSS) score. When only Brief Psychiatric Rating Scale (BPRS) was provided, a transformation to PANSS equivalent score was conducted, using validated conversion tables (Leucht et al. 2013).

© 2024 Gerolymos C et al. *JAMA Network Open.*

- the baseline Simpson Angus Scale (SAS) score and depression severity defined by the baseline Hamilton Depression Rating Scale (HAM-D).
- proportion of first-generation antipsychotic (defined by one of the following ATC codes: chlorpromazine N05AA01, levomepromazine N05AA02, cyamemazine N05AA06, fluphenazine N05AB02, perphenazine N05AB03, thioridazine N05AC02, pipotiazine N05AC04, haloperidol N05AD01, ziprasidone N05AE04, flupentixol, clopenthixol, chlorprothixene, thiothixene, zuclopenthixol N05AF01 – 05, pimozide N05AG02, loxapine N05AH01, clotiapine N05AH06, sulpiride N05AL01), proportion of patients with antipsychotic polytherapy, chlorpromazine equivalent dose (CPZeq) (reported with the lowest effective method calculation if available), adjunctive treatment duration (days) and adjunctive treatment dose. In case of missing CPZeq data, we calculated them using raw data and expert consensus recommendations (Gardner et al. 2010) to convert antipsychotic doses into their chlorpromazine equivalent.
- proportion of participants treated with anticholinergic or benzodiazepine at baseline (not as augmentation drug) and among them, mean daily dose of biperiden, trihexyphenidyl, benztropine, procyclidine, tropatepine, diazepam, lorazepam, nitrazepam, clonazepam and temazepam.
- number of serious adverse events, number of adverse events, proportion of dropouts and proportion of dropouts for tolerance issues.

Missing data

First, last and/or corresponding authors were contacted in case of missing or incomplete data. Missing standard deviation (SD) were calculated from raw data when available. If we couldn't obtain raw data, we calculated missing SD from confidence intervals, SE or p-values (Student t test). If any data wasn't available, we calculated missing means and SDs from raw data if available (Gagrat et al. 1978; Friis et al. 1983; Adler et al. 1986; Kramer et al. 1988; Poyurovsky et al. 2006).

Risk of bias

© 2024 Gerolymos C et al. *JAMA Network Open.*

Two assessors (C.G. and G.F.) analyzed the five criteria of the risk-of-bias assessment tool for each study included in the quantitative meta-analysis: adequate randomization process and concealment; deviations from intended interventions; dealing with missing outcome data; correct measurement of outcome; and selection of reported results. In case of disagreement, discrepancies were managed through discussion between the two assessors and in case of doubt, a third author (L.B.) was contacted for the final decision.

Statistical analysis

We estimated standardized mean differences (SMDs) and their respective standard error (seSMD) for our continuous outcome using pairwise and network meta-analysis. If the total sample size was ≤ 20, Hedges's g (SMD) correction was applied (Hedges 1981). If not, Cohen's d (SMD) was calculated. For multi-arm studies, to avoid double counting and preserve individual treatment contribution, we adopted reducing weights method conceived by Rücker and Schwarzer (Rücker et Schwarzer 2014), recalculating standard errors for each comparison. This method preserves the total residual heterogeneity, measured by the Cochran's Q. To avoid the emergence of intrinsic inconsistencies in the calculation of SMDs for three-arm studies, the SMDs were computed using a common pooled standard deviation from all the associated three treatment groups. Only Cohen's d (SMD) is adequate for multiple-arm studies (Crippa et Orsini 2016). The different effect sizes were compiled using a frequentist random-effects network meta-analysis model and 95% confidence intervals (95%CI) are presented. Frequentist setting gives the same results than Bayesian setting (Shim et al. 2019). Pooled weights for each intervention were calculated from pairwise comparisons model (Rücker et al. 2020). Results were resumed in a forest plot and a league table.

The statistical heterogeneity of our model was assessed with I^2 (Higgins 2003) and τ^2 (Higgins 2008). Cochran's Q test was conducted for overall heterogeneity and inconsistency.

We assessed the publication bias using Egger's test (Egger et al. 1997) of the intercept from the funnel plot constructed. We have verified the consistency of our results by using Pustejovsky-Rodger's correction test (more adapted for continuous effect sizes) (Pustejovsky et Rodgers 2019) and the Thompson-Sharp's test (Thompson et Sharp 1999).

We explored the proportion of direct and indirect comparisons for each face-to-face of interventions. We also evaluated the consistency between direct and indirect evidence through separate indirect from direct evidence (SIDE) back calculation method, with p-value of test of disagreement for each comparison.

© 2024 Gerolymos C et al. *JAMA Network Open.*

The ranking of treatments is presented through P-score, based on the random-effects model. Surface under the cumulative ranking curve (SUCRA) has been used for the rankogram. To examine the transitivity assumption, we listed relevant sociodemographic and clinical factors (cf. 3.3 Data extraction) and compared them by means of boxplots. For continuous variables, we used error bars to present means ± SD according to intervention group.

Sensitivity analyses. Effect size modifiers analyses were carried out for continuous variables (mean age, proportion of men, akathisia treatment duration, akathisia treatment dose, psychotic severity, chlorpromazine equivalent dose, biperiden, trihexyphenidyl, benztropine, diazepam, lorazepam and nitrazepam dose as cotreatments). Five subgroup analyses were carried out limiting analyses to trials (i) with low risk of bias, (ii) with akathisia treatment duration > two days, (iii) using the BARS scale (the most frequent one) vs. other scales. (iv) exploring the efficacy of benzodiazepines (diazepam and clonazepam) and antihistaminics (diphenhydramine and cyproheptadine), (v) by therapeutic classes.

All statistical analyses were performed using the 'netmeta' package (version 2.8-2) in R 4.1.3 (R Foundation, Vienna, Austria; Balduzzi S, 2019).

2. eResults

Supplementary text 2. Results for the efficacy at different time points and the efficacy on subjective and objective symptoms

About the efficacy at different time points, vitamin B6 appears to be effective in reducing subjective akathisia symptoms starting from day three[24,25]. Mianserin is effective from three days in one RCT [16] whereas it is effective from five days on subjective symptoms in another RCT [21]. The same conclusions have been found for trazodone for all subscales [28,29].

About the efficacy on subjective and objective symptoms, mianserin and vitamin B6 are not statistically effective for objective symptoms, but are effective in all other subscales (global, subjective, distress) [24,25]. Mirtazapine and propranolol, tested in Poyurovsky 2006[21], were effective on all subscales, except for distress subscale. Mirtazapine does not reach significance for subjective symptoms and distress subscales in another RCT [26]. Biperiden is effective on objective and subjective subscales but no data were reported for global and distress subscale, and Baskak et al. do not report efficacy for biperiden but the treatment duration was too short (6 hours)[18,23].

eFigure 1. P-score ranking for the 10 interventions treating AIA and placebo.

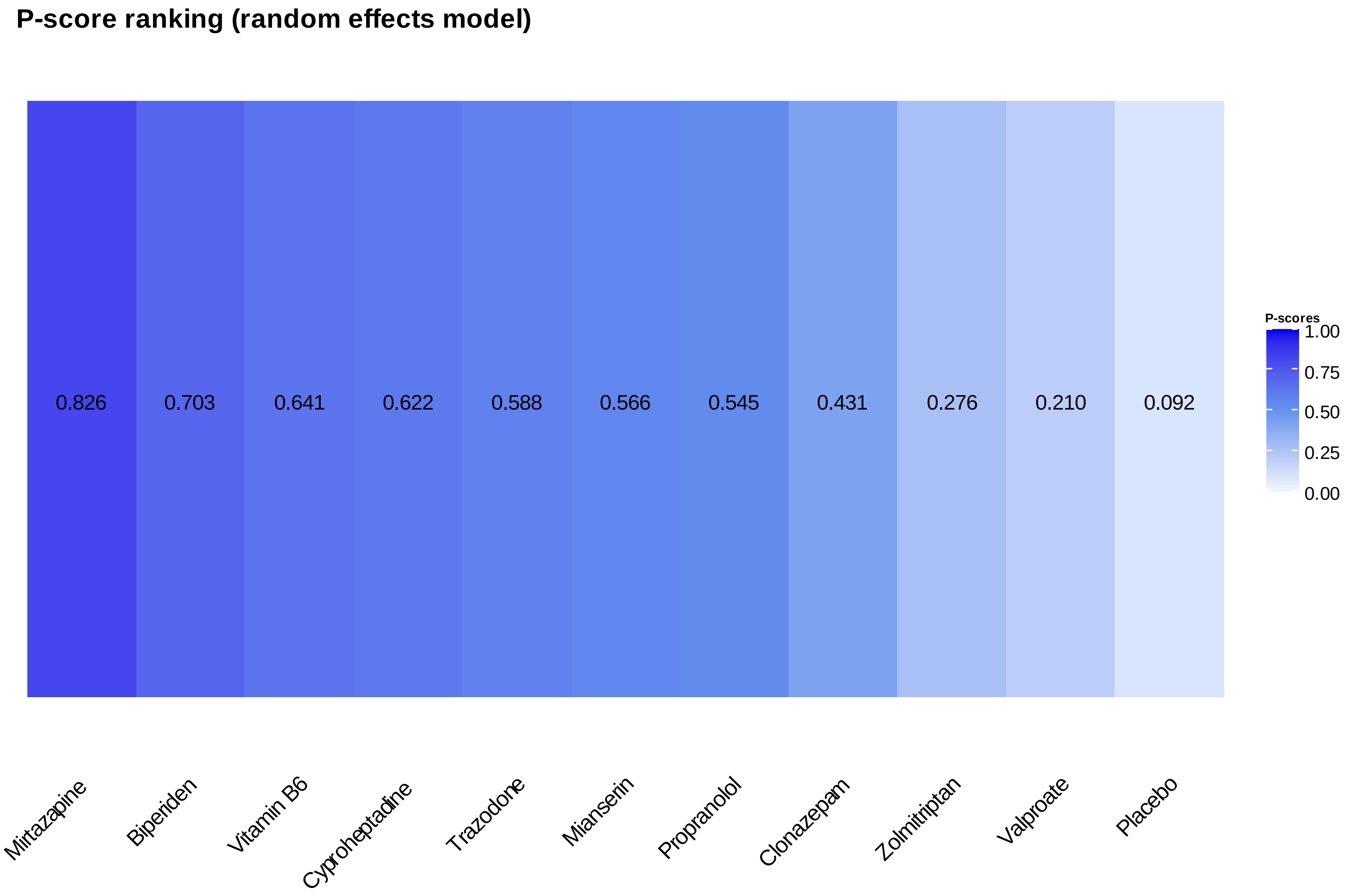

© 2024 Gerolymos C et al. *JAMA Network Open.*

eFigure 2. Bar chart (rankogram) showing Surface Under the Cumulative Ranking (SUCRA) score for the efficacy of the 10 different treatments in AIA and placebo.

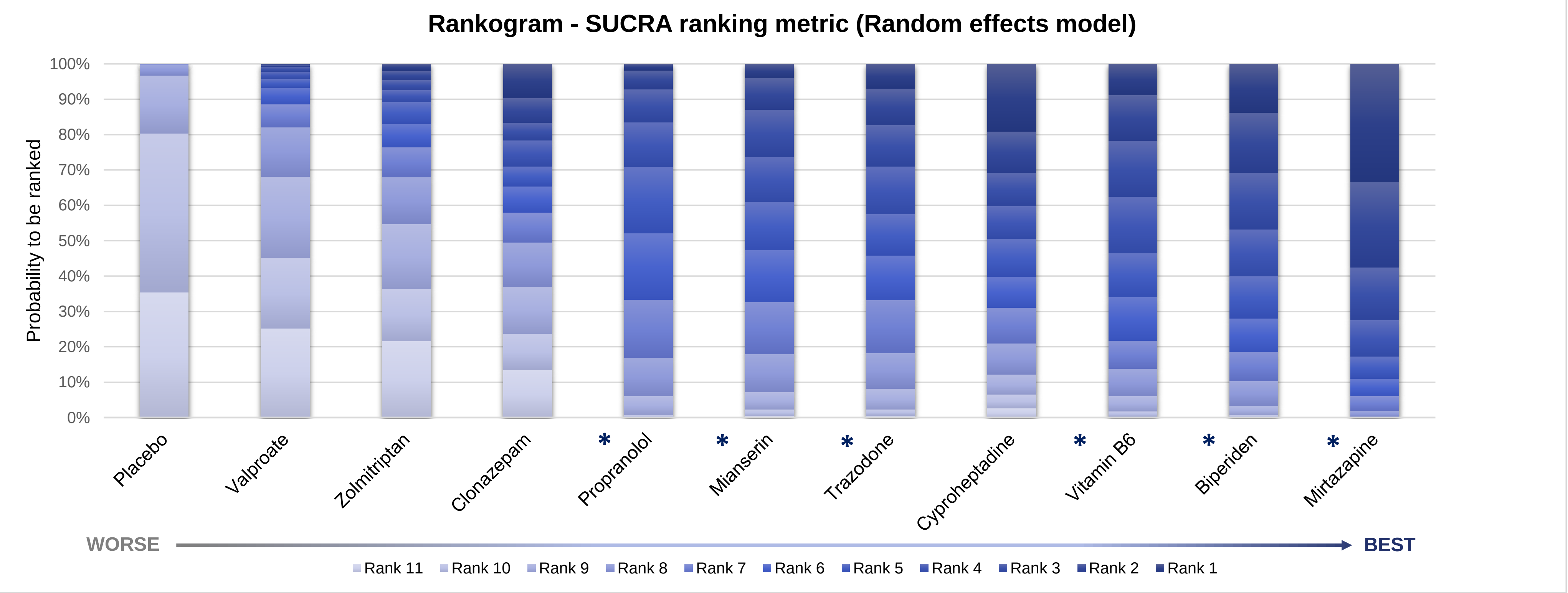

The set of probabilities to be rank from first (rank 1) to eleventh (rank 11) for each intervention is represented in a cumulative bar chart. Raw probabilities are summarized in the cross-tabulation (eTable 5). Darkest colors represent highest ranks, whereas whitest colors represent lowest rank. Asterisks underline interventions for which effect size, as reported in the forest plot (Figure 3), is statistically different than 0.

© 2024 Gerolymos C et al. *JAMA Network Open.*

eFigure 3. Forest plot separating direct and indirect evidence for pairwise comparisons in NMA.

Comparison	Number of Studies	Direct Evidence	I2	SMD	95%-CI
**Biperiden vs Valproate**					
Direct estimate	1	0.86		-0.99	[-1.94; -0.04]
Indirect estimate				0.14	[-2.18; 2.46]
Network estimate				-0.83	[-1.71; 0.05]
**Mianserin vs Placebo**					
Direct estimate	2	0.93	0%	-0.80	[-1.45; -0.15]
Indirect estimate				-1.00	[-3.46; 1.45]
Network estimate				-0.81	[-1.44; -0.19]
**Mianserin vs Vitamin B6**					
Direct estimate	1	0.75		0.00	[-0.84; 0.84]
Indirect estimate				0.41	[-1.03; 1.85]
Network estimate				0.10	[-0.62; 0.83]
**Mirtazapine vs Placebo**					
Direct estimate	2	0.91	0%	-1.34	[-2.00; -0.68]
Indirect estimate				0.18	[-1.91; 2.27]
Network estimate				-1.20	[-1.83; -0.58]
**Mirtazapine vs Propranolol**					
Direct estimate	1	0.77		-0.17	[-0.95; 0.60]
Indirect estimate				-1.22	[-2.63; 0.18]
Network estimate				-0.42	[-1.10; 0.26]
**Propranolol vs Placebo**					
Direct estimate	3	0.95	73%	-0.75	[-1.33; -0.17]
Indirect estimate				-1.49	[-4.04; 1.06]
Network estimate				-0.78	[-1.35; -0.22]
**Valproate vs Placebo**					
Direct estimate	1	0.87		-0.33	[-1.26; 0.60]
Indirect estimate				0.84	[-1.59; 3.28]
Network estimate				-0.18	[-1.05; 0.69]
**Vitamin B6 vs Placebo**					
Direct estimate	2	0.91	0%	-0.98	[-1.67; -0.29]
Indirect estimate				-0.33	[-2.48; 1.83]
Network estimate				-0.92	[-1.57; -0.26]

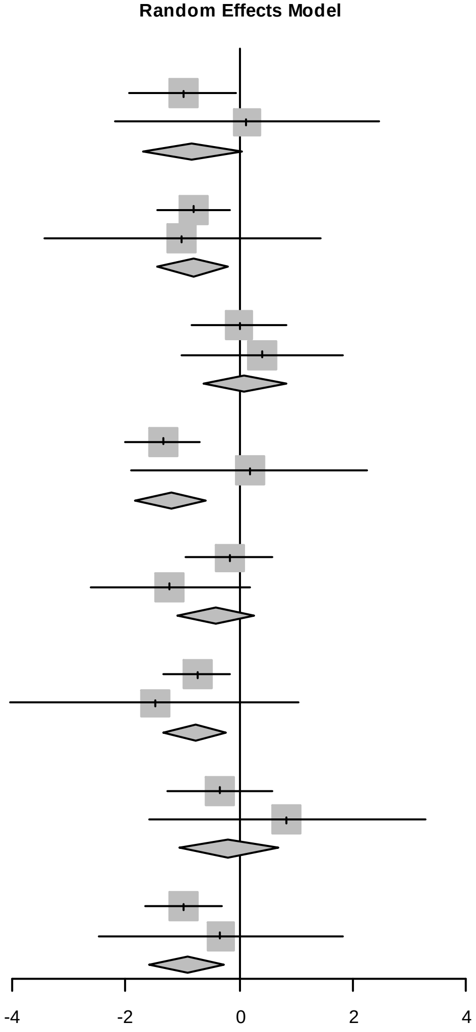

© 2024 Gerolymos C et al. *JAMA Network Open.*

eFigure 4. Plot showing direct and indirect evidence proportions for each network estimate (random-effects model).

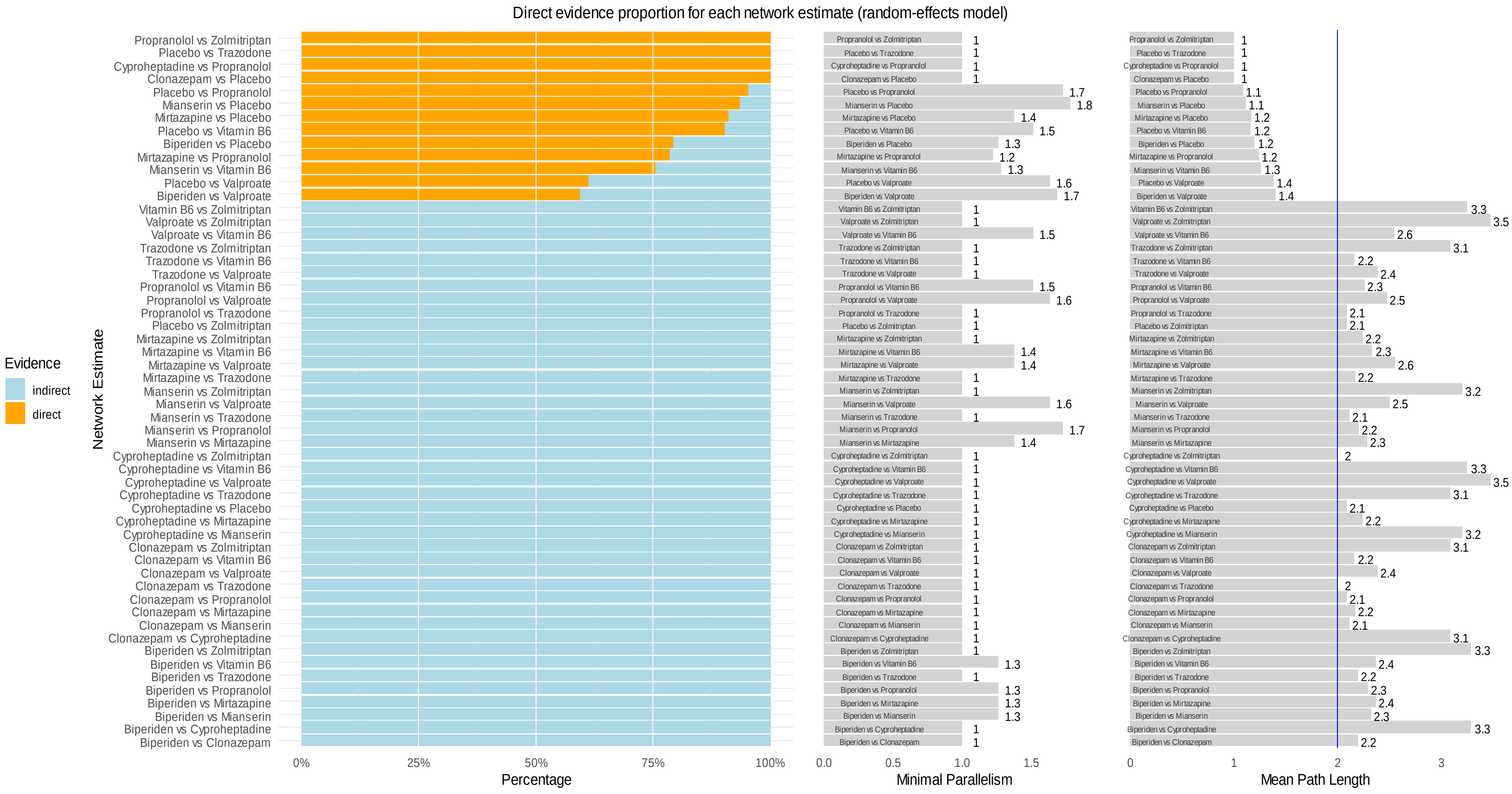

© 2024 Gerolymos C et al. *JAMA Network Open.*

eFigure 5. Funnel plot of NMA showing publication bias assessment.

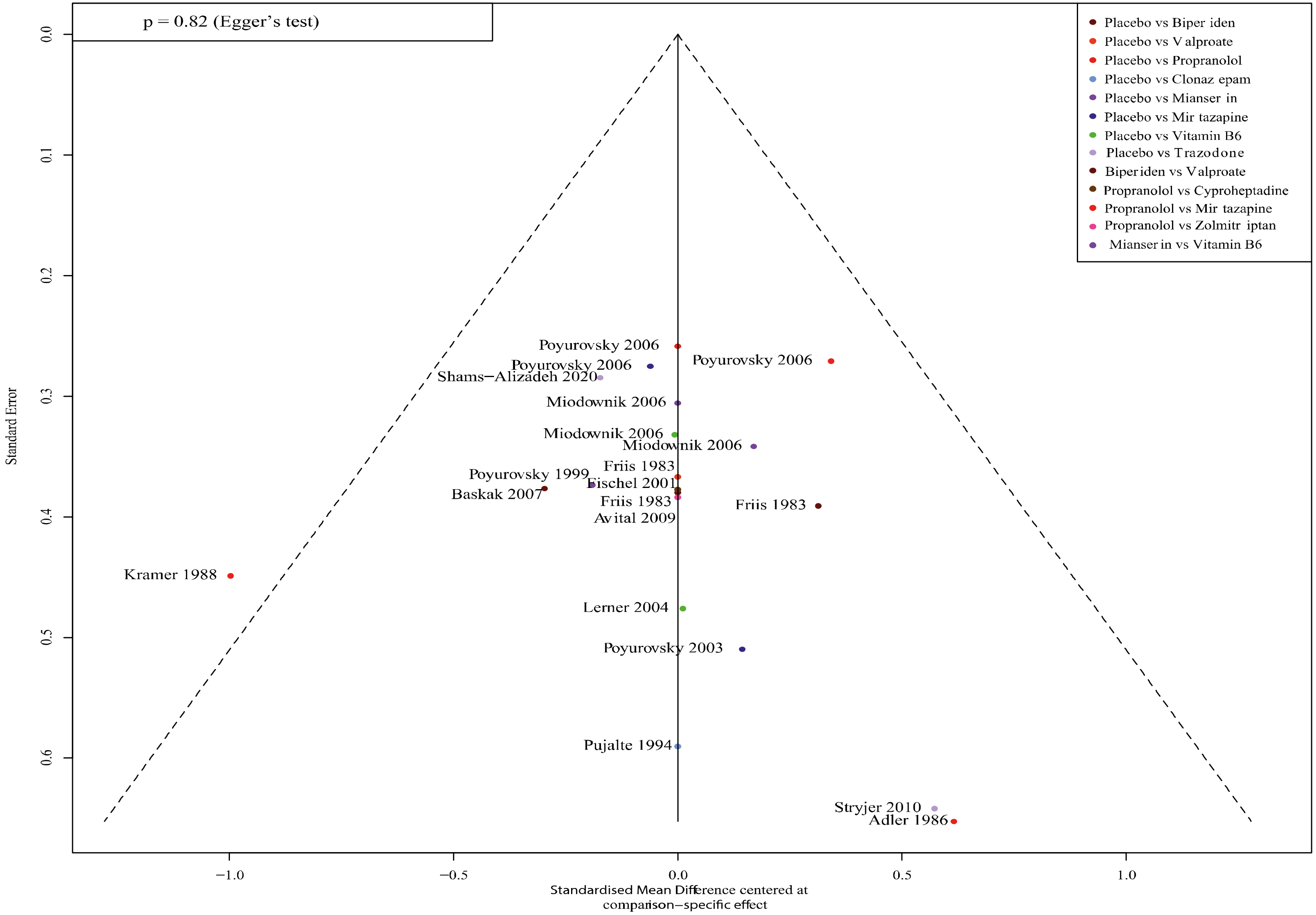

© 2024 Gerolymos C et al. *JAMA Network Open.*

eTable 1. Excluded studies and reasons for exclusion.

Reference	Reason for exclusion
Bjarke J, Gjerde HN, Jørgensen HA, Kroken RA, Løberg EM, Johnsen E. Akathisia and atypical antipsychotics: relation to suicidality, agitation and depression in a clinical trial. Acta Neuropsychiatr. 2022 Oct;34(5):282-288. doi: 10.1017/neu.2022.9. Epub 2022 May 20. PMID: 35260218.	Missing data for the main outcome
Juncal-Ruiz M, Ramirez-Bonilla M, Gomez-Arnau J, Ortiz-Garcia de la Foz V, Suarez-Pinilla P, Martinez-Garcia O, Neergaard KD, Tabares-Seisdedos R, Crespo-Facorro B. Incidence and risk factors of acute akathisia in 493 individuals with first episode non-affective psychosis: a 6-week randomised study of antipsychotic treatment. Psychopharmacology (Berl). 2017 Sep;234(17):2563-2570. doi: 10.1007/s00213-017-4646-1. Epub 2017 May 31. PMID: 28567698.	Missing data for the main outcome
Seemüller F, Schennach R, Mayr A, Musil R, Jäger M, Maier W, Klingenberg S, Heuser I, Klosterkötter J, Gastpar M, Schmitt A, Schlösser R, Schneider F, Ohmann C, Lewitzka U, Gaebel W, Möller HJ, Riedel M; German Study Group on First-Episode Schizophrenia. Akathisia and suicidal ideation in first-episode schizophrenia. J Clin Psychopharmacol. 2012 Oct;32(5):694-8. doi: 10.1097/JCP.0b013e3182677958. PMID: 22926606.	Missing data for the main outcome
Trottier ED, Bailey B, Lucas N, Lortie A. Prochlorperazine in children with migraine: a look at its effectiveness and rate of akathisia. Am J Emerg Med. 2012 Mar;30(3):456-63. doi: 10.1016/j.ajem.2010.12.020. Epub 2011 Feb 5. PMID: 21296523.	No psychiatric patient
de Leon J, Diaz FJ, Aguilar MC, Jurado D, Gurpegui M. Does smoking reduce akathisia? Testing a narrow version of the self-medication hypothesis. Schizophr Res. 2006 Sep;86(1-3):256-68. doi: 10.1016/j.schres.2006.05.009. Epub 2006 Jun 30. PMID: 16814524.	No randomized controlled trial
Gross-Isseroff R, Magen A, Shiloh R, Hermesh H, Weizman A. The 5-HT1D receptor agonist zolmitriptan for neuroleptic-induced akathisia: an open label preliminary study. Int Clin Psychopharmacol. 2005 Jan;20(1):23-5. doi: 10.1097/00004850-200501000-00005. PMID: 15602112.	No randomized controlled trial
Vinson DR. Diphenhydramine in the treatment of akathisia induced by prochlorperazine. J Emerg Med. 2004 Apr;26(3):265-70. doi: 10.1016/j.jemermed.2003.11.011. PMID: 15028322.	No randomized controlled trial
Stryjer R, Strous RD, Bar F, Poyurovsky M, Weizman A, Kotler M. Treatment of neuroleptic-induced akathisia with the 5-HT2A antagonist trazodone. Clin Neuropharmacol. 2003 May-Jun;26(3):137-41. doi: 10.1097/00002826-200305000-00006. PMID: 12782915.	No randomized controlled trial

© 2024 Gerolymos C et al. *JAMA Network Open.*

Reference	Reason for exclusion
Hirose S, Ashby CR. Immediate effect of intravenous diazepam in neuroleptic-induced acute akathisia: an open-label study. J Clin Psychiatry. 2002 Jun;63(6):524-7. doi: 10.4088/jcp.v63n0610. PMID: 12088165.	No randomized controlled trial
Collins RW, Jones JB, Walthall JD, Chisholm CD, Giles BK, Brizendine EJ, Cordell WH. Intravenous administration of prochlorperazine by 15-minute infusion versus 2-minute bolus does not affect the incidence of akathisia: a prospective, randomized, controlled trial. Ann Emerg Med. 2001 Nov;38(5):491-6. doi: 10.1067/mem.2001.119249. PMID: 11679859.	No psychiatric patient
Vinson DR, Migala AF, Quesenberry CP Jr. Slow infusion for the prevention of akathisia induced by prochlorperazine: a randomized controlled trial. J Emerg Med. 2001 Feb;20(2):113-9. doi: 10.1016/s0736-4679(00)00297-3. PMID: 11207403.	Missing data for the main outcome
Hirose S, Ashby CR. Intravenous biperiden in akathisia: an open pilot study. Int J Psychiatry Med. 2000;30(2):185-94. doi: 10.2190/RAFD-AXDF-RJAD-FL1R. PMID: 11001281.	No randomized controlled trial
Sharma AN, Steinberg K, Nelson LS. Akathisia and prochlorperazine. Ann Emerg Med. 2000 Aug;36(2):169-70. doi: 10.1067/mem.2000.108179. PMID: 10918114.	No randomized controlled trial
Duncan EJ, Adler LA, Stephanides M, Sanfilipo M, Angrist B. Akathisia and exacerbation of psychopathology: a preliminary report. Clin Neuropharmacol. 2000 May-Jun;23(3):169-73. doi: 10.1097/00002826-200005000-00008. PMID: 10895402.	Missing data for the main outcome
Poyurovsky M, Weizman A. Lack of efficacy of the 5-HT3 receptor antagonist granisetron in the treatment of acute neuroleptic-induced akathisia. Int Clin Psychopharmacol. 1999 Nov;14(6):357-60. doi: 10.1097/00004850-199911000-00006. PMID: 10565803.	No randomized controlled trial
Drotts DL, Vinson DR. Prochlorperazine induces akathisia in emergency patients. Ann Emerg Med. 1999 Oct;34(4 Pt 1):469-75. doi: 10.1016/s0196-0644(99)80048-1. PMID: 10499947.	Missing data for the main outcome
Gruber O, Northoff G, Pflug B. Subjektives Erleben von Patienten mit akuter neuroleptika-induzierter Akathisie [Subjective experiences of patients with acute neuroleptic-induced akathisia]. Fortschr Neurol Psychiatr. 1998 Dec;66(12):531-8. German. doi: 10.1055/s-2007-995295. PMID: 9922925.	Missing data for the main outcome
Nishimatsu O, Horiguchi J, Inami Y, Sukegawa T, Sasaki A. Periodic limb movement disorder in neuroleptic-induced akathisia. Kobe J Med Sci. 1997 Oct;43(5):169-77. PMID: 9642972.	Sample size < 10
Poyurovsky M, Weizman A. Serotonergic agents in the treatment of acute neuroleptic-induced akathisia: open-label study of buspirone and mianserin. Int Clin Psychopharmacol. 1997 Sep;12(5):263-8. doi: 10.1097/00004850-199709000-00003. PMID: 9466160.	No randomized controlled trial

© 2024 Gerolymos C et al. *JAMA Network Open.*

Reference	Reason for exclusion
Anfang MK, Pope HG Jr. Treatment of neuroleptic-induced akathisia with nicotine patches. Psychopharmacology (Berl). 1997 Nov;134(2):153-6. doi: 10.1007/s002130050436. PMID: 9399378.	No randomized controlled trial
Spivak B, Mester R, Abesgaus J, Wittenberg N, Adlersberg S, Gonen N, Weizman A. Clozapine treatment for neuroleptic-induced tardive dyskinesia, parkinsonism, and chronic akathisia in schizophrenic patients. J Clin Psychiatry. 1997 Jul;58(7):318-22. doi: 10.4088/jcp.v58n0706. PMID: 9269253.	Missing data for the main outcome
Miller CH, Hummer M, Oberbauer H, Kurzthaler I, DeCol C, Fleischhacker WW. Risk factors for the development of neuroleptic induced akathisia. Eur Neuropsychopharmacol. 1997 Feb;7(1):51-5. doi: 10.1016/s0924-977x(96)00041-7. PMID: 9088885.	No randomized controlled trial
DiMartini AF, Trzepacz PT, Daviss SR. Prospective study of FK506 side effects: anxiety or akathisia? Biol Psychiatry. 1996 Sep 1;40(5):407-11. doi: 10.1016/0006-3223(95)00395-9. PMID: 8874843.	Missing data for the main outcome
Foster PN, Stickle BR, Laurence AS. Akathisia following low-dose droperidol for antiemesis in day-case patients. Anaesthesia. 1996 May;51(5):491-4. doi: 10.1111/j.1365-2044.1996.tb07800.x. PMID: 8694168.	No psychiatric patient
Weiss D, Aizenberg D, Hermesh H, Zemishlany Z, Munitz H, Radwan M, Weizman A. Cyproheptadine treatment in neuroleptic-induced akathisia. Br J Psychiatry. 1995 Oct;167(4):483-6. doi: 10.1192/bjp.167.4.483. PMID: 8829717.	No randomized controlled trial
Movin-Osswald G, Karlsson P, Hammarlund-Udenaes M, Farde L. Influence of rate of administration of raclopride on akathisia and prolactin response. Psychopharmacology (Berl). 1994 Mar;114(2):248-56. doi: 10.1007/BF02244845. PMID: 7838916.	No psychiatric patient
Sachdev P, Loneragan C. Intravenous benztropine and propranolol challenges in acute neuroleptic-induced akathisia. Clin Neuropharmacol. 1993 Aug;16(4):324-31. doi: 10.1097/00002826-199308000-00004. PMID: 8104097.	Sample size < 10
Remington G, Fornazzari L, Sethna R. Placebo response in refractory tardive akathisia. Can J Psychiatry. 1993 May;38(4):248-50. doi: 10.1177/070674379303800404. PMID: 8100184.	No randomized controlled trial
Sachdev P, Loneragan C. Low-dose apomorphine challenge in tardive akathisia. Neurology. 1993 Mar;43(3 Pt 1):544-7. doi: 10.1212/wnl.43.3_part_1.544. PMID: 8450998.	Missing data for the main outcome
Sachdev P, Loneragan C. Intravenous benztropine and propranolol challenges in tardive akathisia. Psychopharmacology (Berl). 1993;113(1):119-22. doi: 10.1007/BF02244343. PMID: 7862817.	Sample size < 10

© 2024 Gerolymos C et al. *JAMA Network Open.*

Reference	Reason for exclusion
Horiguchi J, Nishimatsu O. Usefulness of antiparkinsonian drugs during neuroleptic treatment and the effect of clonazepam on akathisia and parkinsonism occurred after antiparkinsonian drug withdrawal: a double-blind study. Jpn J Psychiatry Neurol. 1992 Sep;46(3):733-9. doi: 10.1111/j.1440-1819.1992.tb00549.x. PMID: 1362592.	Additional drugs administered during the course of the study
Adler LA, Angrist B, Fritz P, Rotrosen J, Mallya G, Lipinski JF Jr. Lack of efficacy of d-propranolol in neuroleptic-induced akathisia. Neuropsychopharmacology. 1991 Feb;4(2):109-15. PMID: 1673844.	No randomized controlled trial
Blin O, Durup M, Pailhous J, Serratrice G. Akathisia, motility, and locomotion in healthy volunteers. Clin Neuropharmacol. 1990 Oct;13(5):426-35. doi: 10.1097/00002826-199010000-00004. PMID: 2272022.	No psychiatric patient
Fleischhacker WW, Roth SD, Kane JM. The pharmacologic treatment of neuroleptic-induced akathisia. J Clin Psychopharmacol. 1990 Feb;10(1):12-21. doi: 10.1097/00004714-199002000-00003. PMID: 1968470.	No randomized controlled trial
Miller CH, Fleischhacker WW, Ehrmann H, Kane JM. Treatment of neuroleptic induced akathisia with the 5-HT2 antagonist ritanserin. Psychopharmacol Bull. 1990;26(3):373-6. PMID: 1980375.	No randomized controlled trial
Kutcher S, Williamson P, MacKenzie S, Marton P, Ehrlich M. Successful clonazepam treatment of neuroleptic-induced akathisia in older adolescents and young adults: a double-blind, placebo-controlled study. J Clin Psychopharmacol. 1989 Dec;9(6):403-6. PMID: 2574191.	Population < 18 years old
Adler L, Duncan E, Angrist B, Hemdal P, Rotrosen J, Slotnick V. Effects of a specific beta 2-receptor blocker in neuroleptic-induced akathisia. Psychiatry Res. 1989 Jan;27(1):1-4. doi: 10.1016/0165-1781(89)90002-4. PMID: 2564208.	No randomized controlled trial
Lipinski JF Jr, Keck PE Jr, McElroy SL. Beta-adrenergic antagonists in psychosis: is improvement due to treatment of neuroleptic-induced akathisia? J Clin Psychopharmacol. 1988 Dec;8(6):409-16. PMID: 2906947.	Missing data for the main outcome
Reiter S, Adler L, Angrist B, Corwin J, Rotrosen J. Atenolol and propranolol in neuroleptic-induced akathisia. J Clin Psychopharmacol. 1987 Aug;7(4):279-80. doi: 10.1097/00004714-198708000-00034. PMID: 2887590.	Sample size < 10

© 2024 Gerolymos C et al. *JAMA Network Open.*

Kutcher SP, Mackenzie S, Galarraga W, Szalai J. Clonazepam treatment of adolescents with neuroleptic-induced akathisia. Am J Psychiatry. 1987 Jun;144(6):823-4. doi: 10.1176/ajp.144.6.aj1446823. PMID: 2884890.	Population < 18 years old
Dupuis B, Catteau J, Dumon JP, Libert C, Petit H. Comparison of propranolol, sotalol, and betaxolol in the treatment of neuroleptic-induced akathisia. Am J Psychiatry. 1987 Jun;144(6):802-5. doi: 10.1176/ajp.144.6.802. PMID: 2884889.	No randomized controlled trial

© 2024 Gerolymos C et al. *JAMA Network Open.*

Reference	Reason for exclusion
Bartels M, Heide K, Mann K, Schied HW. Treatment of akathisia with lorazepam. An open clinical trial. Pharmacopsychiatry. 1987 Mar;20(2):51-3. doi: 10.1055/s-2007-1017074. PMID: 2884681.	Missing data for the main outcome
Adler L, Angrist B, Peselow E, Corwin J, Rotrosen J. Noradrenergic mechanisms in akathisia: treatment with propranolol and clonidine. Psychopharmacol Bull. 1987;23(1):21-5. PMID: 2885887.	Sample size < 10
Shaw ED, Mann JJ, Weiden PJ, Sinsheimer LM, Brunn RD. A case of suicidal and homicidal ideation and akathisia in a double-blind neuroleptic crossover study. J Clin Psychopharmacol. 1986 Jun;6(3):196-7. doi: 10.1097/00004714-198606000-00024. PMID: 2872240.	Missing data for the main outcome
Walters A, Hening W, Chokroverty S, Fahn S. Opioid responsiveness in patients with neuroleptic-induced akathisia. Mov Disord. 1986;1(2):119-27. doi: 10.1002/mds.870010206. PMID: 2904116.	Sample size < 10
Van Putten T, May PR, Marder SR. Akathisia with haloperidol and thiothixene. Arch Gen Psychiatry. 1984 Nov;41(11):1036-9. doi: 10.1001/archpsyc.1983.01790220026004. PMID: 6497564.	Missing data for the main outcome
Pringsheim, T., Gardner, D., Addington, D., Martino, D., Morgante, F., Ricciardi, L., ... & Barnes, T. R. (2018). The assessment and treatment of antipsychotic-induced akathisia. *The Canadian Journal of Psychiatry*, *63*(11), 719-729.	No randomized controlled trial
Salem, H., Nagpal, C., Pigott, T., & Lucio Teixeira, A. (2017). Revisiting antipsychotic-induced akathisia: current issues and prospective challenges. Current neuropharmacology, 15(5), 789-798.	Missing data for the main outcome
Hieber, R., Dellenbaugh, T., & Nelson, L. A. (2008). Role of mirtazapine in the treatment of antipsychotic-induced akathisia. Annals of Pharmacotherapy, 42(6), 841-846.	No randomized controlled trial
Forcen, F. E., Matsoukas, K., & Alici, Y. (2016). Antipsychotic-induced akathisia in delirium: a systematic review. Palliative & supportive care, 14(1), 77-84.	No randomized controlled trial
Poyurovsky, M. (2010). Acute antipsychotic-induced akathisia revisited. *The British journal of psychiatry*, *196*(2), 89-91.	Missing data for the main outcome
Shams-Alizadeh, N., Bakhshayesh, H., Rezaei, F., Ghaderi, E., Shams-Alizadeh, N., & Hassanzadeh, K. (2018). Effect of vitamin B6 versus propranolol on antipsychotic-induced akathisia: a pilot comparative double-blind study. *Iranian Journal of Pharmaceutical Research: IJPR*, *17*(Suppl), 130.	Missing data for the main outcome
Kalniunas, A., Chakrabarti, I., Mandalia, R., Munjiza, J., & Pappa, S. (2021). The relationship between antipsychotic-induced akathisia and suicidal behaviour: a systematic review. *Neuropsychiatric Disease and Treatment*, 3489-3497.	Missing data for the main outcome

© 2024 Gerolymos C et al. *JAMA Network Open.*

Reference	Reason for exclusion
Pfeffer, G., Chouinard, G., & Margolese, H. C. (2005). Gabapentin in the treatment of antipsychotic-induced akathisia in schizophrenia. International clinical psychopharmacology, 20(3), 179-181.	No randomized controlled trial
Praharaj, S. K., Kongasseri, S., Behere, R. V., & Sharma, P. S. V. N. (2015). Mirtazapine for antipsychotic-induced acute akathisia: a systematic review and meta-analysis of randomized placebo-controlled trials. Therapeutic advances in psychopharmacology, 5(5), 307-313.	No randomized controlled trial
Poyurovsky, M., & Weizman, A. (2020). Treatment of antipsychotic-induced akathisia: role of serotonin 5-HT 2a receptor antagonists. *Drugs*, *80*, 871-882.	No randomized controlled trial
Jethwa, K. D. (2015). Pharmacological management of antipsychotic-induced akathisia: an update and treatment algorithm. BJPsych Advances, 21(5), 342-344	No randomized controlled trial
LARACH, V. W. (2001). Relationship between antipsychotic-induced akathisia and tardive dyskinesia and suicidality in schizophrenia: impact of clozapine and olanzapine. Acta psychiat. belg, 101, 128-144.	Missing data for the main outcome
Kumar, R., & Sachdev, P. S. (2009). Akathisia and second-generation antipsychotic drugs. Current opinion in psychiatry, 22(3), 293-299.	No randomized controlled trial
Laoutidis, Z. G., & Luckhaus, C. (2014). 5-HT2A receptor antagonists for the treatment of neuroleptic-induced akathisia: a systematic review and meta-analysis. International Journal of Neuropsychopharmacology, 17(5), 823-832.	No randomized controlled trial
Ozyildirim, I., & Kosecioglu, S. (2009). P01-119 mirtazapine induced tardive akathisia: A case report. European Psychiatry, 24, S507.	No randomized controlled trial
Koller, K. (2019). Propranolol for mirtazapine-induced akathisia: Single case report. Mental Health Clinician, 9(1), 61-63.	No randomized controlled trial
Miller, C. H., & Fleischhacker, W. W. (2000). Managing antipsychotic-induced acute and chronic akathisia. Drug Safety, 22, 73-81.	No randomized controlled trial
Kim, E. S., & Heo, Y. A. (2021). Manage antipsychotic-induced akathisia by making changes to the antipsychotic drug regimen and/or adding anti-akathisia agents. Drugs & Therapy Perspectives, 37, 70-74.	No randomized controlled trial
Demyttenaere, K., Detraux, J., Racagni, G., & Vansteelandt, K. (2019). Medication-induced akathisia with newly approved antipsychotics in patients with a severe mental illness: a systematic review and meta-analysis. CNS drugs, 33, 549-566.	No randomized controlled trial
Furuse, T., & Hashimoto, K. (2010). Fluvoxamine for blonanserin-associated akathisia in patients with schizophrenia: report of five cases. Annals of General Psychiatry, 9, 1-4.	No randomized controlled trial

© 2024 Gerolymos C et al. *JAMA Network Open.*

Kane, J. M., Fleischhacker, W. W., Hansen, L., Perlis, R., Pikalov III, A., & Assunção-Talbott, S. (2009). Akathisia: an updated review focusing on second-generation antipsychotics. *Journal of clinical Psychiatry*, *70*(5), 627.	No randomized controlled trial

Reference	Reason for exclusion
Takeshima, M., Ishikawa, H., Kanbayashi, T., & Shimizu, T. (2018). Gabapentin enacarbil for antipsychotic induced akathisia in schizophrenia patients: a pilot open-labeled study. Neuropsychiatric Disease and Treatment, 3179-3184.	No randomized controlled trial
Raveendranathan, D., & Swaminath, G. R. (2015). Mirtazapine induced akathisia: Understanding a complex mechanism. Indian journal of psychological medicine, 37(4), 474-475.	Missing data for the main outcome
Yoshimura, B., Sato, K., Sakamoto, S., Tsukahara, M., Yoshimura, Y., & So, R. (2019). Incidence and predictors of acute akathisia in severely ill patients with first-episode schizophrenia treated with aripiprazole or risperidone: secondary analysis of an observational study. Psychopharmacology, 236, 723-730.	No randomized controlled trial
Poyurovsky, M., & Weizman, A. (2015). Treatment of antipsychotic-related akathisia revisited: the role of serotonin 2A receptor antagonists. Journal of Clinical Psychopharmacology, 35(6), 711-714.	No randomized controlled trial
Kim, J. H., & Byun, H. J. (2007). Association of subjective cognitive dysfunction with akathisia in patients receiving stable doses of risperidone or haloperidol. Journal of clinical pharmacy and therapeutics, 32(5), 461-467.	Missing data for the main outcome
Ranjan, S., Chandra, P. S., Chaturvedi, S. K., Prabhu, S. C., & Gupta, A. (2006). Atypical antipsychotic–induced akathisia with depression: therapeutic role of mirtazapine. Annals of Pharmacotherapy, 40(4), 771-774.	No randomized controlled trial
Barnes, T. R. (2003). The Barnes Akathisia rating scale–revisited. Journal of Psychopharmacology, 17(4), 365-370.	Missing data for the main outcome
Saito, Y., Takekuma, Y., Furuta, M., & Sugawara, M. (2022). Pregabalin Attenuates Carboplatin-Induced Akathisia-Like Neuropathy: A Novel Case Report. Case reports in oncology, 14(3), 1418-1421.	No psychiatric patient
Resende Lima, A., Soares-Weiser, K., Bacaltchuk, J., Barnes, T. R., & Cochrane Schizophrenia Group. (1996). Benzodiazepines for neuroleptic-induced acute akathisia. Cochrane Database of Systematic Reviews, 2010(1).	No randomized controlled trial
Forcen, F. E., Root, J. C., & Alici, Y. (2017). Antipsychotic-induced akathisia in cancer settings. Psycho-oncology, 26(7), 1053.	Missing data for the main outcome

© 2024 Gerolymos C et al. *JAMA Network Open.*

Veeraraghavan, V., & Srinivasan, K. (2022). Role of serotonin receptor antagonist cyproheptadine in treatment-resistant akathisia. Annals of Indian Psychiatry, 6(1), 102-104.	No randomized controlled trial
Poyurovsky, M., & Weizman, A. (2018). Very low-dose Mirtazapine (7.5 mg) in treatment of acute antipsychotic-associated akathisia. Journal of clinical psychopharmacology, 38(6), 609-611.	No randomized controlled trial

Reference	Reason for exclusion
AYAYDIN, H., & ULGAR, Ş. B. (2018). Successful treatment of paliperidone palmitate induced akathisia with mirtazapine: A case report. Harran Üniversitesi Tıp Fakültesi Dergisi, 15(3), 259-261.	No randomized controlled trial
Morgan, J. C. (2019). Treatment of Tardive Akathisia. Therapy of Movement Disorders: A Case-Based Approach, 297-299.	Missing data for the main outcome
Poyurovsky, M., Bergman, J., Pashinian, A., & Weizman, A. (2014). Beneficial effect of low-dose mirtazapine in acute aripiprazole-induced akathisia. International clinical psychopharmacology, 29(5), 296-298.	No randomized controlled trial
Braude, D., & Boling, S. (2006). Case report of unrecognized akathisia resulting in an emergency landing and RSI during air medical transport. Air Medical Journal, 25(2), 85-87.	No randomized controlled trial
Patel, J., & Marwaha, R. (2022). Akathisia. In StatPearls [Internet]. StatPearls Publishing.	Missing data for the main outcome
Furuse, T., & Hashimoto, K. (2010). Fluvoxamine for aripiprazole-associated akathisia in patients with schizophrenia: a potential role of sigma-1 receptors. Annals of general psychiatry, 9, 1-3.	No randomized controlled trial
Shah, R., Grover, S., Maheshwari, U., Kate, N., & Malhotra, N. (2010). Acute akathisia with quetiapine: A case report and review of literature. Indian journal of pharmacology, 42(6), 416.	No randomized controlled trial
Oflaz S, Bakay H, Cekic E, et al. Atypical antipsychotics induced chronic akathisia: a case report. Journal of Mood Disorders 2014; 4: 175.	No randomized controlled trial
Gokcay, H., Solmaz, M., & Balcioglu, Y. H. (2021). Akathisia Related to Multivitamin Complex Supplemented With Ginseng Extract. American Journal of Therapeutics, 28(6), e808-e809.	No randomized controlled trial
Kern, D., & Lang, A. E. (2015). Acute akathisia. In Medication-induced movement disorders (pp. 3-19). Cambridge: Cambridge University Press.	Missing data for the main outcome
Hansen, L. (2001). A critical review of akathisia, and its possible association with suicidal behaviour. Human Psychopharmacology: Clinical and Experimental, 16(7), 495-505.	Missing data for the main outcome

© 2024 Gerolymos C et al. *JAMA Network Open.*

Citrome, L., Yatham, L. N., Patel, M. D., Barabássy, Á., Hankinson, A., & Earley, W. R. (2021). Cariprazine and akathisia, restlessness, and extrapyramidal symptoms in patients with bipolar depression. Journal of Affective Disorders, 288, 191-198.	Missing data for the main outcome
Saboowala, H. (Ed.). (2020). What is Akathisia--or not sitting?-A Drug Induced Emotional Turmoil–DIET. Dr. Hakim Saboowala.	No randomized controlled trial
Sethuram, K., & Gedzior, J. (2014). Akathisia: case presentation and review of newer treatment agents. Psychiatric Annals, 44(8), 391-396.	No randomized controlled trial
Lohr, J. B., Eidt, C. A., Alfaraj, A. A., & Soliman, M. A. (2015). The clinical challenges of akathisia. CNS spectrums, 20(S1), 1-16.	No randomized controlled trial

Reference	Reason for exclusion
Sienaert, P., van Harten, P., & Rhebergen, D. (2019). The psychopharmacology of catatonia, neuroleptic malignant syndrome, akathisia, tardive dyskinesia, and dystonia. Handbook of clinical neurology, 165, 415-428.	No randomized controlled trial
Forcen, F. E. (2015). Akathisia: is restlessness a primary condition or an adverse drug effect. Curr Psychiatry, 14(1), 14-8.	Missing data for the main outcome
Desai, A., Nierenberg, D. W., & Duhaime, A. C. (2010). Akathisia after mild traumatic head injury: Case report. Journal of Neurosurgery: Pediatrics, 5(5), 460-464.	No randomized controlled trial
Musco, S., McAllister, V., & Caudle, I. (2020). Dopamine-receptor blocking agent-associated akathisia: a summary of current understanding and proposal for a rational approach to treatment. Therapeutic Advances in Psychopharmacology, 10, 2045125320937575.	No randomized controlled trial
Back, I., & Taubert, M. (2007). Akathisia and an unusual symptomatic treatment: a case report. Palliative Medicine, 21(8), 713.	No randomized controlled trial
Nepal, H., Black, E., & Bhattarai, M. (2016). Self-Harm in Sertraline-Induced Akathisia| Psychiatrist. com. The Primary Care Companion for CNS Disorders, 18(6), 26843.	Missing data for the main outcome
Wilson, M. S. (2005). Mirtazapine for akathisia in bipolar disorder. Journal of clinical psychopharmacology, 25(4), 394-395.	No randomized controlled trial
Albert, N., Catthoor, K., & Morrens, M. (2022). Akathisia after chronic usage of synthetic cathinones: A case study. Frontiers in Psychiatry, 13.	No randomized controlled trial
Markoula, S., Konitsiotis, S., Chatzistefanidis, D., Lagos, G., & Kyritsis, A. P. (2010). Akathisia induced by mirtazapine after 20 years of continuous treatment. Clinical neuropharmacology, 33(1), 50-51.	Missing data for the main outcome
Hansen, L. K., L'Allemand, T., Thiry, F., & Baldwin, D. S. (2010). Structured relaxation in the treatment of akathisia: case series. Neuropsychiatric disease and treatment, 269-271.	No randomized controlled trial
Tachere, R. O., & Modirrousta, M. (2017). Beyond anxiety and agitation: A clinical approach to akathisia. Australian Family Physician, 46(5), 296-298.	No randomized controlled trial

Schoretsanitis, G., Nikolakopoulou, A., Guinart, D., Correll, C. U., & Kane, J. M. (2020). Iron homeostasis alterations and risk for akathisia in patients treated with antipsychotics: a systematic review and meta-analysis of cross-sectional studies. European neuropsychopharmacology, 35, 1-11.	No randomized controlled trial
Escobar-Córdoba, F., Álvarez-Vanegas, C., & Torres-Espinosa, L. (2015). Pharmacological treatment of neuroleptic-induced akathisia. Acta Neurológica Colombiana, 31(4), 447-453.	No randomized controlled trial
Advokat, C. (2010). A brief overview of iatrogenic akathisia. Clinical Schizophrenia & Related Psychoses, 3(4), 226-236.	No randomized controlled trial

Reference	Reason for exclusion
Kane, J. M., Barnes, T. R., Correll, C. U., Sachs, G., Buckley, P., Eudicone, J., ... & Assunção-Talbott, S. (2010). Evaluation of akathisia in patients with schizophrenia, schizoaffective disorder, or bipolar I disorder: a post hoc analysis of pooled data from short-and long-term aripiprazole trials. Journal of Psychopharmacology, 24(7), 1019-1029.	Missing data for the main outcome
Gulsun, M., & Doruk, A. (2008). Mirtazapine-induced akathisia. *Journal of clinical psychopharmacology*, *28*(4), 467.	Missing data for the main outcome
ÖZTURAN, D. D., & DEMİR, F. Ü. (2021). Mirtazapine Induced Akathisia: A Case Report. *Middle Black Sea Journal of Health Science*, *7*(3), 436-438.	Missing data for the main outcome
Catalano, G., Grace, J. W., Catalano, M. C., Morales, M. J., & Cruse, L. M. (2005). Acute akathisia associated with quetiapine use. Psychosomatics, 46(4), 291-301.	Missing data for the main outcome
Gómez-Arnau Ramírez, J. (2016). Pharmacological and non-pharmacological correlates of acute akathisia in first-episode psychosis.	Missing data for the main outcome
Elhusein, B., Mahgoub, O., Kumar, R., & Abdullah, M. A. The Possibility of the Second Generation Antipsychotic-Olanzapine to Cause Severe Akathisia in a Drug Naive Patient-A Case Report.	No randomized controlled trial
Zareifopoulos, N., Katsaraki, M., Stratos, P., Villiotou, V., Skaltsa, M., Dimitriou, A., ... & Velissaris, D. (2021). Pathophysiology and management of Akathisia 70 years after the introduction of the chlorpromazine, the first antipsychotic. European Review for Medical and Pharmacological Sciences, 25(14), 4746-4756.	No randomized controlled trial
Ali, N., Rasheed, A., Tariq, I., Qadir, A., Sarwar, A., Atta, R., & Maqsood, U. (2018). Incidence of Drug Induced Akathisia with Aripiprazole and Risperidone: A Comparative Study. National Journal of Health Sciences, 3(1), 20-22.	Missing data for the main outcome

© 2024 Gerolymos C et al. *JAMA Network Open.*

ALI, N., TARIQ, I., RASHID, A., KHAN, A. Q., ALI, N., & HABIB, U. (2017). THE HIGH INCIDENCE ILLUSION: AKATHISIA WITH ARIPIPRAZOLE. Pakistan Postgraduate Medical Journal, 28(1), 7-9.	Missing data for the main outcome
Luthra, V., Pinninti, N. R., Yoder, K., Musthaq, M. S., Umapathy, C., & Levinson, D. F. (2000). Is akathisia associated with poor clinical response to antipsychotics during acute hospital treatment?. General hospital psychiatry, 22(4), 276-280.	Missing data for the main outcome
Obyedkov, V., Gorgun, O., Hodjaev, A., Dakukina, T., Hvostova, I., Kaminskaya, Y., ... & Kuchynskaya, A. (2022). Clinical and Psychopathological Phenomenology of Mild Acute Drug-Induced Akathisia.	No randomized controlled trial
Ersche, K. D., Cumming, P., Craig, K. J., Müller, U., Fineberg, N. A., Bullmore, E. T., & Robbins, T. W. (2012). Amisulpride-induced acute akathisia in OCD: an example of dysfunctional dopamine–serotonin interactions?. Journal of Psychopharmacology, 26(6), 887-890.	Missing data for the main outcome

Reference	Reason for exclusion
Hirose, S. (2003). The causes of underdiagnosing akathisia. Schizophrenia Bulletin, 29(3), 547-558.	No randomized controlled trial
Demir, B., Sancaktar, M., & Altindag, A. (2021). Lithium-induced treatment-resistant akathisia: A case report and literature overview. Clinical Neuropharmacology, 44(3), 112-113.	No randomized controlled trial
Tucci, V., Calvo, J. A., Moukaddam, N., Waheed, A., & Wilson, M. P. (2020). Psychiatric Emergencies for Clinicians: Emergency Department Management of Acute Drug-Induced Akathisia. Journal of Emergency Medicine, 58(6), 922-926.	Missing data for the main outcome
Morton, A., Mackle, C., & Pavey, J. (2022). Akathisia and oculogyric crisis in hyperemesis gravidarum. *Obstetric Medicine*, 1753495X221137942.	No psychiatric patient
Naguy, A., Moodliar-Rensburg, S., & Alamiri, B. (2021). Mirtazapine treatment for azithromycin-associated akathisia. American Journal of Therapeutics, 28(6), e751-e752.	No randomized controlled trial
Beardmore, L. (2020). The Psychosocial Effects of Drug-Induced Akathisia (Doctoral dissertation, University of Manchester).	Missing data for the main outcome
SACHDEV, P., & Loneragan, C. (1991). The present status of akathisia. The Journal of nervous and mental disease, 179(7), 381-391.	No randomized controlled trial
Hirjak, D., Kubera, K. M., Bienentreu, S., Thomann, P. A., & Wolf, R. C. (2019). Antipsychotic-induced motor symptoms in schizophrenic psychoses—Part 1: Dystonia, akathisia und parkinsonism. Der Nervenarzt, 90, 1-11.	Missing data for the main outcome
Thompson, H. (2021). Implementation of an Akathisia Scale Into the Mental Health Assessment to Screen for Early-Onset Akathisia.	Missing data for the main outcome
Jaitpal, V., & Gawande, S. (2022). Olanzapine-Induced Parkinsonism and Akathisia: A Case Report. Cureus, 14(1).	No randomized controlled trial

© 2024 Gerolymos C et al. *JAMA Network Open.*

Adler, L. A., Peselow, E., Rosenthal, M., & Angrist, B. (1993). A controlled comparison of the effects of propranolol, benztropine, and placebo on akathisia: an interim analysis. Psychopharmacology bulletin, 29(2), 283–286.	Report not retrieved
Dumon, J. P., Catteau, J., Lanvin, F., & Dupuis, B. A. (1992). Randomized, double-blind, crossover, placebo-controlled comparison of propranolol and betaxolol in the treatment of neuroleptic-induced akathisia. The American journal of psychiatry, 149(5), 647–650.	Sample not matching target population

© 2024 Gerolymos C et al. *JAMA Network Open.*

eTable 2. Characteristics of the included studies comprised in network meta-analysis.

First Author	PY	Country	Akathisia scale	Design
**Gagrat**	1978	USA	Original Q.	Parallel groups
**Friis**	1983	Denmark	Original Q.	3-arms, cross-over.
**Adler**	1986	USA	SAS	Cross-over
**Kramer**	1988	USA	ARS	Cross-over
**Pujalte**	1994	France	BARS	Parallel groups
**Poyurovsky**	1999	Israel	BARS	Parallel groups
**Fischel**	2001	Israel	BARS	Parallel groups
**Poyurovsky**	2003	Israel	BARS	Parallel groups
**Lerner**	2004	Israel	BARS	Parallel groups
**Miodownik**	2006	Israel	BARS	3-arms
**Poyurovsky**	2006	Israel	BARS	3-arms
**Baskak**	2007	Turkey	BARS	Parallel groups
**Avital**	2009	Israel	BARS	Parallel groups
**Stryjer**	2010	Israel	BARS	Parallel groups
**Shams-Alizadeh**	2020	Israel	BARS	Parallel groups

PY: Publication year;
Original Q.: original questionnaire; SAS: Simpson Angus Scale; ARS: Akathisia Rating Scale; BARS: Barnes Akathisia Rating Scale

© 2024 Gerolymos C et al. *JAMA Network Open.*

Description of scales:

Barnes Akathisia Rating Scale (BARS):

- 4-items, with objective, subjective and global assessment,
- Cohen's kappa varies between 0.74 and 0.95 (Barnes 2003).

Akathisia Rating Scale (ARS):

- 10-items, based on Braude et al. (Braude et al. 1983),
- Factors able to distinguish akathisia from other illness-related movements $p < 0.001$-0.02, in principal component analysis (PCA) (Braude et al. 1983).

Simpson Angus Scale (SAS):

- 1-item, objective rating,
- Cronbach $\alpha = 0.79$ (Janno et al. 2005).

Two different original questionnaires (designed for the trial):

- The original scale in Gagrat et al. (Gagrat et al. 1978) was defined as follows: a score of 0 correspond to no akathisia symptoms, 1 to subjective inner restlessness, 2 to objective restlessness, 3 to severe objective restlessness.

- The original scale in Friis et al. (Friis et al. 1983) was defined as follows: for subjective symptoms, a score of 0 corresponds to absence of symptom, 1 to slight feeling of restlessness, slight muscle sensation in legs and slight anxiety or irritation, 2 to moderate feeling of restlessness, strong wish to move or concentration difficulties, dysphoric mood, anxiety and/or irritation, 3 to strong feeling of restlessness, compulsion to move, totally occupied by the symptoms, sleeping problems and agitation, psychosis aggravation. For objective signs, a score 0 correspond to absence, 1 to can sit still up to one hour and slight movements and feet tapping, 2 to can sit still up to 15 minutes, often leg movements and feet tapping, sometimes lifting the arms and stands or walks around most of the time, 3 to cannot sit still, constant arm and leg movements, rocking of the body and pacing.

© 2024 Gerolymos C et al. *JAMA Network Open.*

First Author	PY	N	Arm 1 (n)	Arm 2 (n)	Arm 3 (n)	Reference group
**Gagrat**	1978	20	Diazepam (9)	Diphenhydramine (11)	-	Diphenhydramine
**Friis**	1983	45	Valproate (15)	Biperiden (15)	Placebo (15)	Placebo
**Adler**	1986	12	Propranolol (6)	Placebo (6)	-	Placebo
**Kramer**	1988	20	Propranolol (10)	Placebo (10)	-	Placebo
**Pujalte**	1994	12	Clonazepam (6)	Placebo (6)	-	Placebo
**Poyurovsky**	1999	30	Mianserin (15)	Placebo (15)	-	Placebo
**Fischel**	2001	29	Cyproheptadine (17)	Propranolol (12)	-	Propranolol
**Poyurovsky**	2003	26	Mirtazapine (13)	Placebo (13)	-	Placebo
**Lerner**	2004	20	Vitamin B6 (10)	Placebo (10)	-	Placebo
**Miodownik**	2006	60	Mianserin (20)	Vitamin B6 (23)	Placebo (17)	Placebo
**Poyurovsky**	2006	90	Mirtazapine (30)	Propranolol (30)	Placebo (30)	Placebo
**Baskak**	2007	30	Biperiden (15)	Placebo (15)	-	Placebo
**Avital**	2009	33	Zolmitriptan (14)	Propranolol (19)	-	Propranolol
**Stryjer**	2010	13	Trazodone (8)	Placebo (5)	-	Placebo
**Shams-Alizadeh**	2020	52	Trazodone (26)	Placebo (26)	-	Placebo

© 2024 Gerolymos C et al. *JAMA Network Open.*

The sample sizes of the trials ranged from 12 to 90 participants. The mean duration of intervention was 5.5 days (Standard deviation (SD) = 4.0). Out of the total participants, 309 (63.4%) were male, and the mean age was 36 (SD = 5). Eight studies (53.3%) recruited patients from Israel, three (20.0%) from the USA, and one each from Denmark, France, Turkey, and Iran (6.7% each).

The distribution of diagnoses was as follows: 348 (80.0%) had schizophrenia, 49 (11.3%) had schizoaffective disorder, 12 (2.8%) had bipolar disorder, nine (2.1%) had schizophreniform disorder, five (1.1%) had delusional disorder, three (0.7%) had psychotic depression, three (0.7%) had a personality disorder, three (0.7%) had alcoholic dementia, one (0.2%) had obsessive-compulsive disorder, one (0.2%) had an anxiety disorder, and one (0.2%) had another psychotic disorder. One study (43) did not report any DSM-5 diagnoses for its participants (52 participants).

Among all participants, 384 (80.2%) were treated with first-generation antipsychotics, 99 (21.0%) were treated with second-generation antipsychotics (defined by one of the following ATC codes: sertindole N05AE03, lurasidone N05AE05, clozapine N05AH02, olanzapine N05AH03, quetiapine N05AH04, sultopride N05AL02, amisulpride N05AL05, risperidone N05AX08, aripiprazole N05AX12, paliperidone N05AX13, cariprazine N05AX15, brexpiprazole N05AX16), and 7 (1.5%) received a combination of first and second-generation antipsychotics. One trial (Stryjer et al. 2010) did not report the type of antipsychotics taken by participants (13 participants). The mean PANSS severity score was 72 (SD = 24). The mean chlorpromazine equivalent dose was 814 mg/day (SD = 520). The CPZeq of trials are on average below 700 mg/d, except for three RCTs assessing propranolol (Adler et al. 1986; Kramer et al. 1988) and clonazepam (Pujalte et al. 1994).

The allowance of benzodiazepines and anticholinergics as cotreatments were mentioned in respectively eight RCTs (Adler et al. 1986; Pujalte et al. 1994; Poyurovsky et al. 1999, 2006; Fischel et al. 2001; Baskak et al. 2007; Avital et al. 2009; Stryjer et al. 2010)(53.3%) and thirteen RCTs (Friis et al. 1983; Adler et al. 1986; Kramer et al. 1988; Pujalte et al. 1994; Poyurovsky et al. 1999, 2006; Fischel et al. 2001; Lerner et al. 2004; Miodownik et al. 2006; Baskak et al. 2007; Avital et al. 2009; Stryjer et al. 2010; Shams-Alizadeh et al. 2020)(86.7%) and unknown in respectively six RCTs (Friis et al. 1983; Kramer et al. 1988; Poyurovsky et al. 2003; Lerner et al. 2004; Miodownik et al. 2006; Shams-Alizadeh et al. 2020)(40.0%) and one RCT(Poyurovsky et al. 2003)(6.7%). Cotreatments with benzodiazepines and anticholinergics were not allowed (exclusion criterion) in respectively four RCTs (Pujalte et al. 1994; Fischel et al. 2001; Baskak et al. 2007; Stryjer et al. 2010)(26.7%) and five RCTs (Friis et al. 1983; Fischel et al. 2001; Baskak et al. 2007; Stryjer et al. 2010; Shams-Alizadeh et al. 2020)(33.3%).

© 2024 Gerolymos C et al. *JAMA Network Open.*

eTable 3. Subnetwork for Gagrat 1978 with treatment effect size estimate and calculation method.

	Experimental	Control	SMD	seSMD	95% CI
**Gagrat 1978**	Diazepam	Diphenhydramine	-0.122	0.450	[-1.0034; 0.7602]

Number of studies	Number of pairwise comparisons	Number of treatments
1	1	2

In Gagrat 1978, SDs were missing. Student's t-tests (df = 19) for independent samples were conducted. We know that the mean differences of score between diazepam and diphenhydramine aren't statistically significant at the different time-points. Using the homoscedasticity hypothesis of Student's test and a not significant p-value, we can estimate the pooled value of SD for the two samples. We simulated results with multiple not significant p-values.

The most likely value of SDs is near studies sharing similar characteristics in our NMA (Friis 1983). Calculations leads to: pooled SD = 0.63; p-value = 0.39. The effect size is compatible with a small effect of diazepam comparing to diphenhydramine in NIA, matching with conclusions of Gagrat et al.

SMD: standardized mean difference; seSMD: standard error of SMD.

© 2024 Gerolymos C et al. *JAMA Network Open.*

eTable 4. Characteristics of the interventions comprised in the network meta-analysis.

Experimental (or control)	Total number of patients across NMA	Total number of studies	Therapeutic class	Hypothetic therapeutic action(s) on NIA
**Diazepam**	9	1	Benzodiazepines (long half-life)	Γ-aminobutyric acid receptor agonist
**Clonazepam**	6	1		
**Diphenhydramine**	11	1	Antihistaminics	5-HT_2 antagonistic activity
**Cyproheptadine**	17	1		
**Propranolol**	77	5	β-adrenergic blocker	Central and peripherical effects of beta-blocking
**Valproate**	15	1	Mood stabilizer / Antiepileptic	Γ-aminobutyric acid mimetic drug
**Biperiden**	30	2	Anticholinergic	Stop increasing acetylcholine turn-over
**Mianserin**	35	2	Antidepressants (α_2-blockers)	5-HT_{2A} antagonistic activity
**Mirtazapine**	43	2		
**Trazodone**	34	2	Antidepressant (5-HT_{2A} blocker)	Post-synaptic antagonism 5-HT_{2A} and 5-HT_{2C}
**Vitamin B6**	33	2	Water-soluble vitamin	Co-factor in decarboxylation from dopa to dopamine, antioxidant, free radical scavenger
**Zolmitriptan**	14	1	Triptan (5-HT_{1D} agonist)	Inhibition of 5-HT_{1D} presynaptic auto-receptor
**Placebo**	168	12	-	-

12 studies have placebo as control group. Three studies have an active treatment as control group: propranolol (n = 2), diphenhydramine (n = 1).

© 2024 Gerolymos C et al. *JAMA Network Open.*

eTable 5. Cross-tabulation of ranking probabilities for the 10 akathisia interventions and placebo.

	Rank 1	Rank 2	Rank 3	Rank 4	Rank 5	Rank 6	Rank 7	Rank 8	Rank 9	Rank 10	Rank 11
**Placebo**	0	0	0	0	0	0	0.001	0.032	0.163	0.45	0.354
**Valproate**	0.003	0.005	0.014	0.02	0.025	0.047	0.065	0.14	0.229	0.199	0.253
**Zolmitriptan**	0.02	0.026	0.028	0.033	0.063	0.066	0.084	0.133	0.183	0.147	0.217
**Clonazepam**	0.097	0.069	0.05	0.074	0.056	0.074	0.085	0.124	0.134	0.102	0.135
**Propranolol**	0.018	0.054	0.093	0.126	0.188	0.187	0.164	0.109	0.054	0.007	0
**Mianserin**	0.041	0.088	0.134	0.127	0.137	0.146	0.147	0.108	0.049	0.018	0.005
**Trazodone**	0.07	0.103	0.117	0.134	0.118	0.125	0.15	0.101	0.059	0.017	0.006
**Cyproheptadine**	0.191	0.116	0.095	0.092	0.107	0.088	0.101	0.088	0.057	0.038	0.027
**Vitamin B6**	0.088	0.129	0.159	0.159	0.124	0.123	0.08	0.077	0.043	0.015	0.003
**Biperiden**	0.138	0.169	0.161	0.132	0.119	0.095	0.082	0.07	0.027	0.007	0
**Mirtazapine**	0.334	0.241	0.149	0.103	0.063	0.049	0.041	0.018	0.002	0	0

© 2024 Gerolymos C et al. *JAMA Network Open.*

eTable 6. Results for Separate indirect from direct evidence (SIDE) using back-calculation method, testing agreement between direct and indirect evidence.

Comparison	k	Prop	NMA	Direct	Indirect	Diff	Z	p-value
Biperiden vs Clonazepam	0	0	-0.4552	.	-0.4552	.	.	.
Biperiden vs Cyproheptadine	0	0	-0.1026	.	-0.1026	.	.	.
Biperiden vs Mianserin	0	0	-0.1993	.	-0.1993	.	.	.
Biperiden vs Mirtazapine	0	0	0.1907	.	0.1907	.	.	.
Biperiden vs Placebo	2	1.00	-1.0129	-1.0129	.	.	.	.
Biperiden vs Propranolol	0	0	-0.2296	.	-0.2296	.	.	.
Biperiden vs Trazodone	0	0	-0.1695	.	-0.1695	.	.	.
Biperiden vs Valproate	1	0.86	-0.8315	-0.9939	0.1385	-1.1324	-0.88	0.38
Biperiden vs Vitamin B6	0	0	-0.0953	.	-0.0953	.	.	.
Biperiden vs Zolmitriptan	0	0	-0.7240	.	-0.7240	.	.	.
Clonazepam vs Cyproheptadine	0	0	0.3526	.	0.3526	.	.	.
Clonazepam vs Mianserin	0	0	0.2559	.	0.2559	.	.	.
Clonazepam vs Mirtazapine	0	0	0.6460	.	0.6460	.	.	.
Clonazepam vs Placebo	1	1.00	-0.5577	-0.5577	.	.	.	.
Clonazepam vs Propranolol	0	0	0.2256	.	0.2256	.	.	.
Clonazepam vs Trazodone	0	0	0.2857	.	0.2857	.	.	.
Clonazepam vs Valproate	0	0	-0.3763	.	-0.3763	.	.	.
Clonazepam vs Vitamin B6	0	0	0.3599	.	0.3599	.	.	.
Clonazepam vs Zolmitriptan	0	0	-0.2688	.	-0.2688	.	.	.
Cyproheptadine vs Mianserin	0	0	-0.0966	.	-0.0966	.	.	.
Cyproheptadine vs Mirtazapine	0	0	0.2934	.	0.2934	.	.	.
Cyproheptadine vs Placebo	0	0	-0.9103	.	-0.9103	.	.	.
Cyproheptadine vs Propranolol	1	1.00	-0.1270	-0.1270	.	.	.	.
Cyproheptadine vs Trazodone	0	0	-0.0668	.	-0.0668	.	.	.
Cyproheptadine vs Valproate	0	0	-0.7289	.	-0.7289	.	.	.
Cyproheptadine vs Vitamin B6	0	0	0.0073	.	0.0073	.	.	.
Cyproheptadine vs Zolmitriptan	0	0	-0.6214	.	-0.6214	.	.	.
Mianserin vs Mirtazapine	0	0	0.3900	.	0.3900	.	.	.
Mianserin vs Placebo	2	0.93	-0.8136	-0.8004	-1.0046	0.2042	0.16	0.87
Mianserin vs Propranolol	0	0	-0.0304	.	-0.0304	.	.	.

© 2024 Gerolymos C et al. *JAMA Network Open.*

**Mianserin vs Trazodone**	0	0	0.0298	.	0.0298	.	.	.
**Mianserin vs Valproate**	0	0	-0.6322	.	-0.6322	.	.	.
**Mianserin vs Vitamin B6**	1	0.75	0.1039	0.0000	0.4076	-0.4076	-0.48	0.63
**Mianserin vs Zolmitriptan**	0	0	-0.5248	.	-0.5248	.	.	.
**Mirtazapine vs Placebo**	2	0.91	-1.2037	-1.3415	0.1799	-1.5214	-1.36	0.17
**Mirtazapine vs Propranolol**	1	0.77	-0.4204	-0.1747	-1.2215	1.0468	1.28	0.20
**Mirtazapine vs Trazodone**	0	0	-0.3602	.	-0.3602	.	.	.
**Mirtazapine vs Valproate**	0	0	-1.0222	.	-1.0222	.	.	.
**Mirtazapine vs Vitamin B6**	0	0	-0.2861	.	-0.2861	.	.	.
**Mirtazapine vs Zolmitriptan**	0	0	-0.9148	.	-0.9148	.	.	.
**Propranolol vs Placebo**	3	0.95	-0.7833	-0.7469	-1.4902	0.7433	0.56	0.58
**Trazodone vs Placebo**	2	1.00	-0.8434	-0.8434	.	.	.	.
**Valproate vs Placebo**	1	0.87	-0.1814	-0.3313	0.8448	-1.1761	-0.88	0.38
**Vitamin B6 vs Placebo**	2	0.91	-0.9176	-0.9780	-0.3258	-0.6522	-0.57	0.57
**Zolmitriptan vs Placebo**	0	0	-0.2889	.	-0.2889	.	.	.
**Propranolol vs Trazodone**	0	0	0.0602	.	0.0602	.	.	.
**Propranolol vs Valproate**	0	0	-0.6019	.	-0.6019	.	.	.
**Propranolol vs Vitamin B6**	0	0	0.1343	.	0.1343	.	.	.
**Propranolol vs Zolmitriptan**	1	1.00	-0.4944	-0.4944	.	.	.	.
**Trazodone vs Valproate**	0	0	-0.6620	.	-0.6620	.	.	.
**Trazodone vs Vitamin B6**	0	0	0.0741	.	0.0741	.	.	.
**Trazodone vs Zolmitriptan**	0	0	-0.5546	.	-0.5546	.	.	.
**Valproate vs Vitamin B6**	0	0	0.7361	.	0.7361	.	.	.
**Valproate vs Zolmitriptan**	0	0	0.1075	.	0.1075	.	.	.
**Vitamin B6 vs Zolmitriptan**	0	0	-0.6287	.	-0.6287	.	.	.

k - Number of studies providing direct evidence ; Prop - Direct evidence proportion ; NMA - Estimated treatment effect (SMD) in network meta-analysis ; Direct - Estimated treatment effect (SMD) derived from direct evidence ; Indirect - Estimated treatment effect (SMD) derived from indirect evidence ; Diff - Difference between direct and indirect treatment estimates ; Z - z-value of test for disagreement (direct versus indirect) ; p-value - p-value of test for disagreement (direct versus indirect).

P-values ≤ 0.10 for SIDE-test means that there is a disagreement between direct and indirect evidence for the considered comparison.

© 2024 Gerolymos C et al. *JAMA Network Open.*

eTable 7. Egger's test details (publication bias assessment).

Egger's test			
**Intercept**	**95% CI**	**t**	**p-value**
-0.546	[-4.97; 3.87]	-0.242	**0.81**

eTable 8. Pustejovsky-Rodgers's corrected test details (for standard error of SMD) (publication bias assessment).

Pustejovsky-Rodger's correction			
**Intercept**	**95% CI**	**t**	**p-value**
-0.241	[-5.72; 5.24]	-0.086	**0.93**

eTable 9. Thompson-Sharp's test details (publication bias assessment).

Thompson-Sharp's test			
**Intercept**	**95% CI**	**t**	**p-value**
-0.5712	[-0.574; -0.569]	0.501	**0.62**

© 2024 Gerolymos C et al. *JAMA Network Open.*

eTable 10. Tolerability outcome details: adverse events for the 10 akathisia interventions and placebo (meta-analysis level)

Treatment	N patients in NMA (without dropouts)	**Number** of patients with **adverse events (%)**						
		Drowsiness	Dizziness	Lightheadedness	Headache	Nausea or vomiting	Hypersalivation	Dry mouth
**Mirtazapine**	34	**11 (32.4%)**	**4 (11.8%)**	0	2 (5.9%)	0	0	5 (14.7%)
**Biperiden**	29	0	0	0	1 (3.4%)	1 (3.4%)	2 (6.9%)	**10 (34.5%)**
**Vitamin B6**	33	0	0	0	0	0	0	0
**Cyproheptadine**	16	0	0	0	0	0	0	0
**Trazodone**	33	**9 (27.3%)**	**5 (15.2%)**	3 (9.1%)	0	0	0	3 (9.1%)
**Mianserin**	31	0	0	0	0	0	0	0
**Propranolol**	61	8 (13.1%)	3 (4.9%)	0	2 (3.3%)	0	0	3 (4.9%)
**Clonazepam**	6	MD	MD	MD	MD	MD	MD	MD
**Zolmitriptan**	8	0	0	0	0	0	0	0
**Valproate**	15	0	0	0	0	0	**5 (33.3%)**	**5 (33.3%)**
**Diazepam**	9	NS*	0	0	0	0	0	0
**Diphenhydramine**	11		0	0	0	0	0	0
**Placebo**	202	13 (6.4%)	1 (0.5%)	6 (3.0%)	0	2 (1.0%)	2 (1.0%)	11 (5.4%)

A frequent reason for dropout is lack of efficacy / persistent or worsening AIA, observed in 9/43 patients (20.9%) for mirtazapine, 27/168 (16.0%) for placebo, 4/35 (11.4%) for mianserin and 6/77 (7.8%) for propranolol. All dropouts for mirtazapine occur between three and five days after the initiation of therapy[21,26]. Overall, severe adverse events occur for only two patients: one patient suffered from severe bradycardia with propranolol and one patient has an allergic reaction to biperiden. Other motives for treatment discontinuation were delusions for zolmitriptan

© 2024 Gerolymos C et al. *JAMA Network Open.*

(n=5) and propranolol (n=4), a switch of antipsychotic for zolmitriptan (n=1), adverse events (without further specification) for zolmitriptan (n=5), moderate sedation for cyproheptadine (n=1), and no reported motives for discontinuation for the other augmentation drugs.

NMA: network meta-analysis;
NS*: non statistically significant difference between diazepam and diphenhydramine, no count reported by authors (Gagrat et al.).

Treatment	N patients in NMA (without dropouts)	**Number** of patients with **adverse events (%)**				
		Perspiration	Sedation	Vision disturbances	Hypotension	Palpitations
**Mirtazapine**	34	0	0	4 (11.8%)	0	2 (5.9%)
**Biperiden**	29	0	**14 (48.3%)**	2 (6.9%)	0	0
**Vitamin B6**	33	0	0	0	0	0
**Cyproheptadine**	16	0	0	0	0	0
**Trazodone**	33	3 (9.1%)	0	0	0	0
**Mianserin**	31	0	0	0	0	0
**Propranolol**	61	0	0	2 (3.3%)	**4 (6.6%)**	3 (4.9%)
**Clonazepam**	6	MD	MD	MD	MD	MD
**Zolmitriptan**	8	0	0	0	0	0
**Valproate**	15	0	**8 (53.3%)**	0	0	0
**Diazepam**	9	0	0	0	0	0
**Diphenhydramine**	11	0	0	0	0	0
**Placebo**	202	1 (0.5%)	10 (5.0%)	5 (2.5%)	0	3 (1.5%)

© 2024 Gerolymos C et al. *JAMA Network Open.*

Treatment	N patients in NMA (without dropouts)	**Number** of patients with **adverse events (%)**				
		Hypokinesia	Increased sexual desire	Transient sedation	Depression	Transient orthostatic hypotension
**Mirtazapine**	34	0	0	0	0	0
**Biperiden**	29	2 (6.9%)	0	0	0	2 (6.9%)
**Vitamin B6**	33	0	0	0	0	0
**Cyproheptadine**	16	0	0	0	0	0
**Trazodone**	33	0	0	0	0	0
**Mianserin**	31	0	0	**8 (25.8%)**	0	1 (3.2%)
**Propranolol**	61	0	0	0	0	0
**Clonazepam**	6	MD	MD	MD	MD	MD
**Zolmitriptan**	8	0	0	0	0	0
**Valproate**	15	0	0	0	**4 (26.7%)**	0
**Diazepam**	9	0	0	0	0	0
**Diphenhydramine**	11	0	0	0	0	0
**Placebo**	202	1 (0.5%)	1 (0.5%)	3 (1.5%)	2 (1.0%)	2 (1.0%)

© 2024 Gerolymos C et al. *JAMA Network Open.*

eTable 11. Acceptability outcome details: dropouts for the 10 akathisia interventions and placebo (meta-analysis level)

Treatment	N patients in NMA	Number of dropout (%)	Motive(s) for dropout
**Mirtazapine**	43	9 (20.9%)	Intolerance to persistant or worsening NIA
**Biperiden**	30	1 (3.3%)	Allergic reaction
**Vitamin B6**	33	0	N/A
**Cyproheptadine**	17	1 (5.9%)	Moderate sedation
**Trazodone**	34	1 (2.9%)	Missing data
**Mianserin**	35	4 (11.4%)	Intolerance to persistant NIA
**Propranolol**	77	16 (20.8%)	Intolerance to persistant NIA (n = 6), delusions (n = 4), severe bradycardia (n = 1), adverse events (n = 5)
**Clonazepam**	6	0	N/A
**Zolmitriptan**	14	6 (42.9%)	Delusions (n = 5), switch of neuroleptic treatment (n = 1)
**Valproate**	15	0	N/A
**Diazepam**	9	0	N/A
**Diphenhydramine**	11	0	N/A
**Placebo**	168	28 (12.2%)	Intolerance to persistant or worsening NIA (n = 27), missing data (n = 1)

© 2024 Gerolymos C et al. *JAMA Network Open.*

3. Transitivity assumption

To explore the transitivity assumption, we present below, clustered by intervention arm:

- Boxplots (for qualitive parameters).
- Error bars (for continuous parameters), that represent mean of the parameter considered plus or minus its standard deviation.

For boxplots: When boxplots show ratios, a ratio of 1.0 indicates 100% for the parameter considered (men ratio, schizophrenic patients, patients treated with first-generation antipsychotics).

For error bars: They show mean ± standard deviation (SD) and simply display the variance of the continuous parameter considered.

For anticholinergics and benzodiazepines data: They present data of participants treated by cotreatment drugs, not used as active treatment and only for the trials allowing their use. These cotreatment drugs have been initiated before the start of the relevant trials.

Regarding demographic characteristics, the patients participating in the NMA do not substantially differ in terms of sex ratio and age, except for two outlier studies. One trial had a sample consisting of 100% men, testing propranolol(Kramer et al. 1988) and one trial with the lowest proportion of men of 40% in the placebo group (Baskak et al. 2007). The diagnosis of schizophrenia was present in 60% or more of the participants, except for the vitamin B6 arms. Only biperiden shows a wide range of treatment duration, ranging from one day to 14 days. The daily treatment dose ranges from 2 to 12 mg for biperiden, from 20 to 120 mg for propranolol, from 50 to 100 mg for trazodone, and from 600 to 1200 mg for vitamin B6. At baseline, the psychotic severity measured by PANSS score in the experimental group ranged between 50 and 100 points, with the exception of three extremes: one biperiden arm (112 pts), one mirtazapine arm (42 pts), and one propranolol arm (38 pts). More than 60% of the participants were treated with first-generation antipsychotics, except for the trazodone arm (only 34.6%). The chlorpromazine equivalent dose was distributed below 1000 mg/day, except for two propranolol arms (1354 and 1047 mg/day) and the clonazepam arm (1416 mg/day).

© 2024 Gerolymos C et al. *JAMA Network Open.*

eFigure 6. Boxplot showing men ratio clustered by intervention.

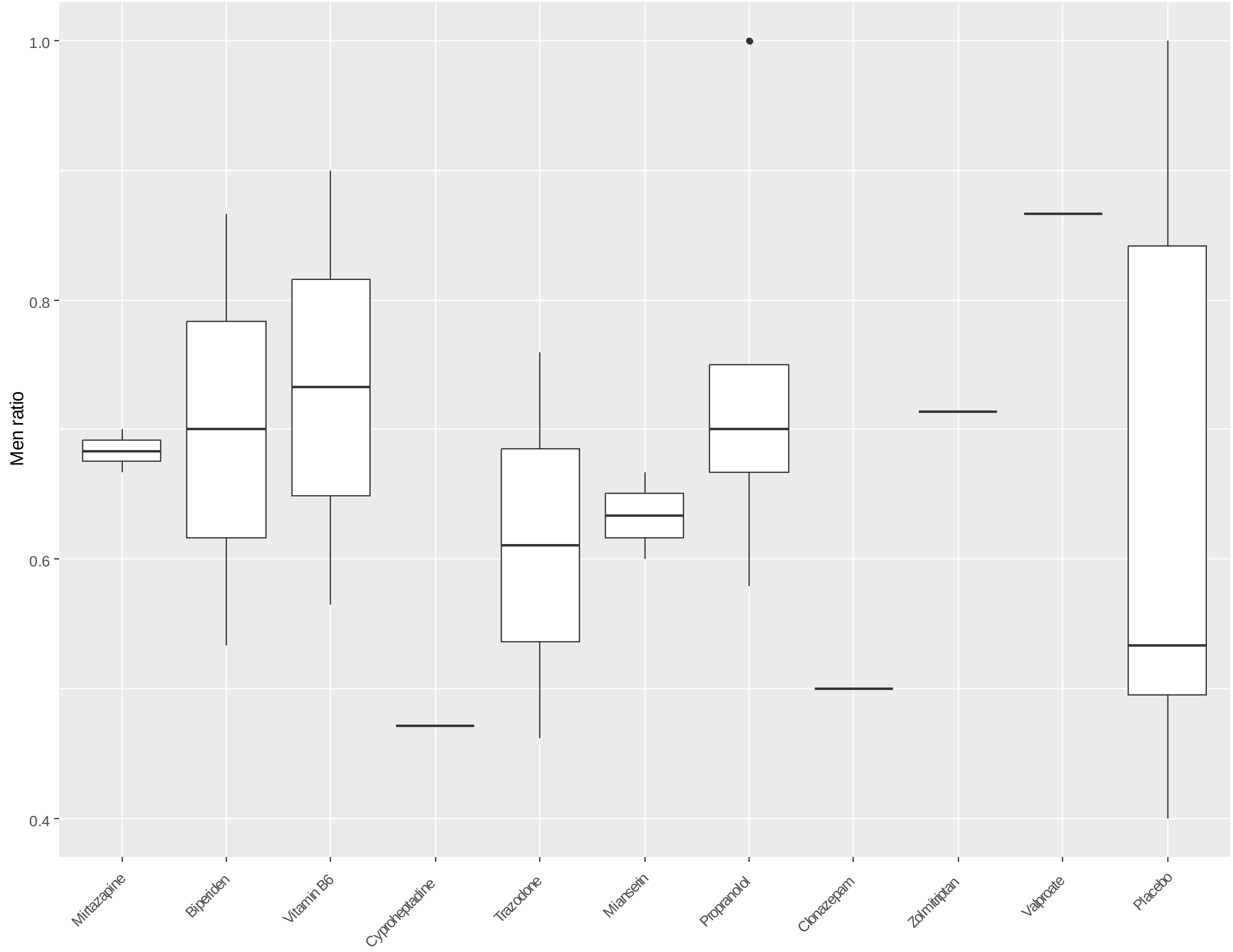

© 2024 Gerolymos C et al. *JAMA Network Open.*

eFigure 7. Boxplot showing age (mean) clustered by intervention.

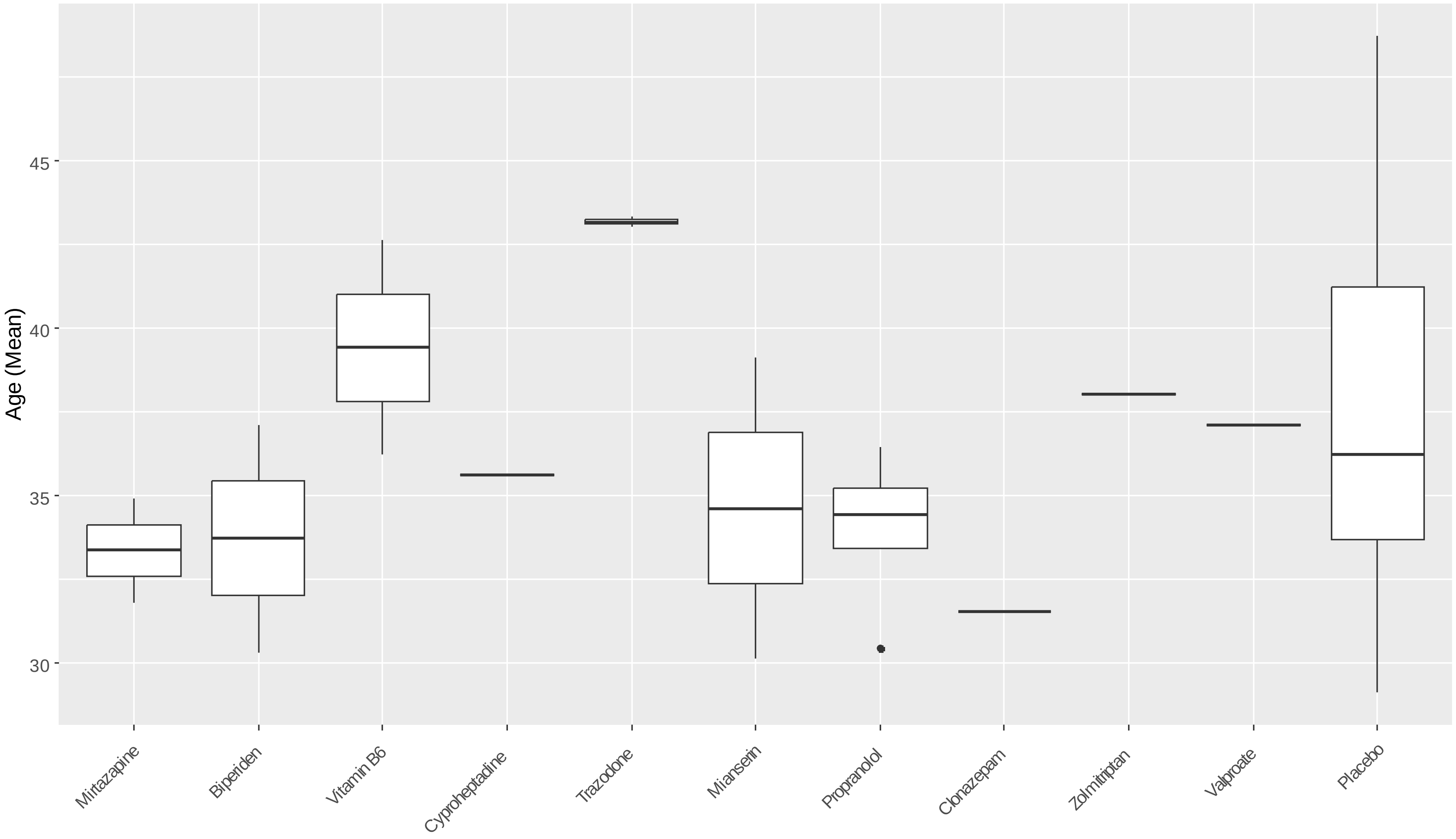

© 2024 Gerolymos C et al. *JAMA Network Open.*

eFigure 8. Error bars showing mean age ± standard deviation clustered by intervention.

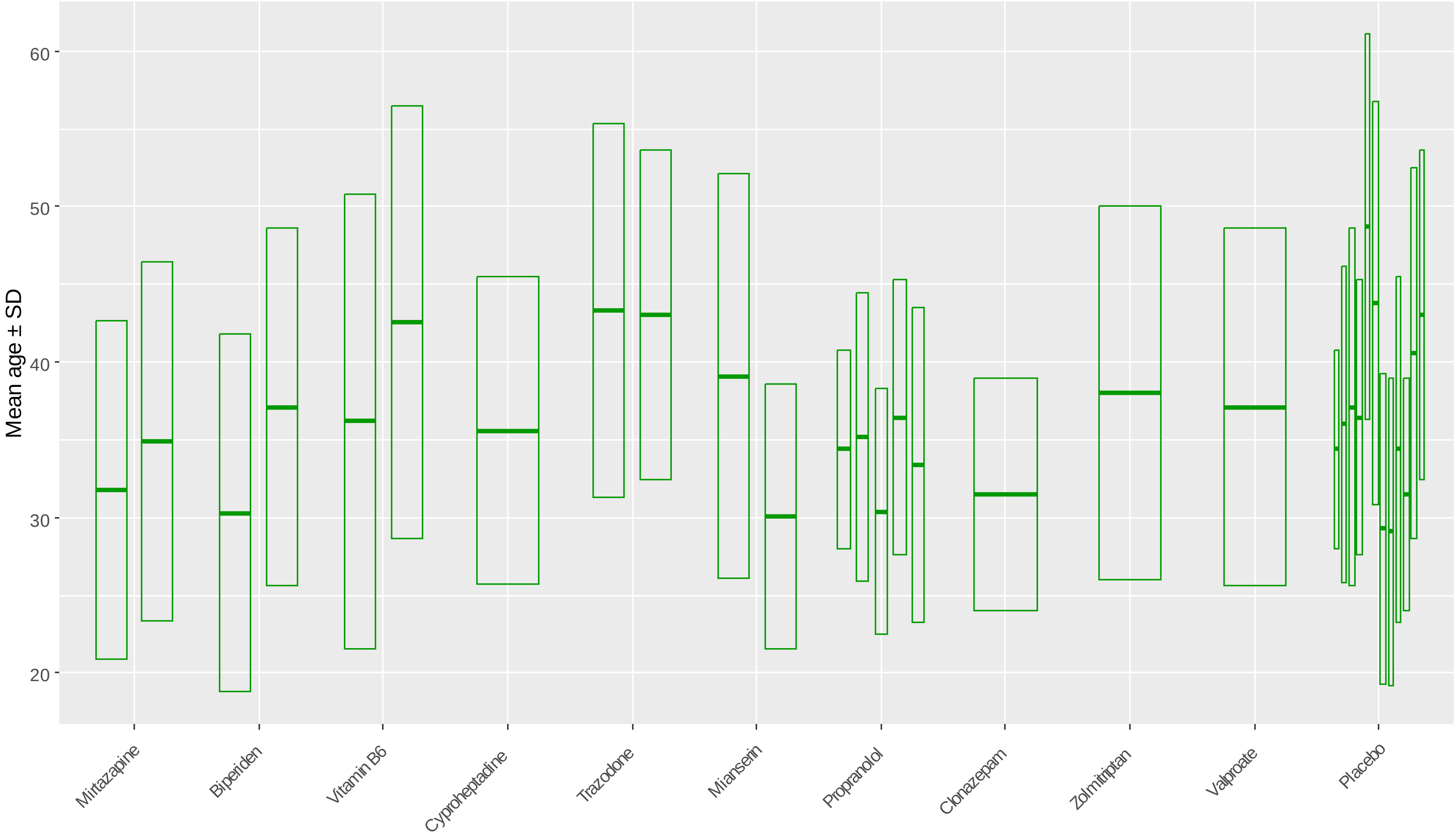

© 2024 Gerolymos C et al. *JAMA Network Open.*

eFigure 9. Boxplot showing schizophrenia ratio (DSM-5 diagnosis) in participants clustered by intervention.

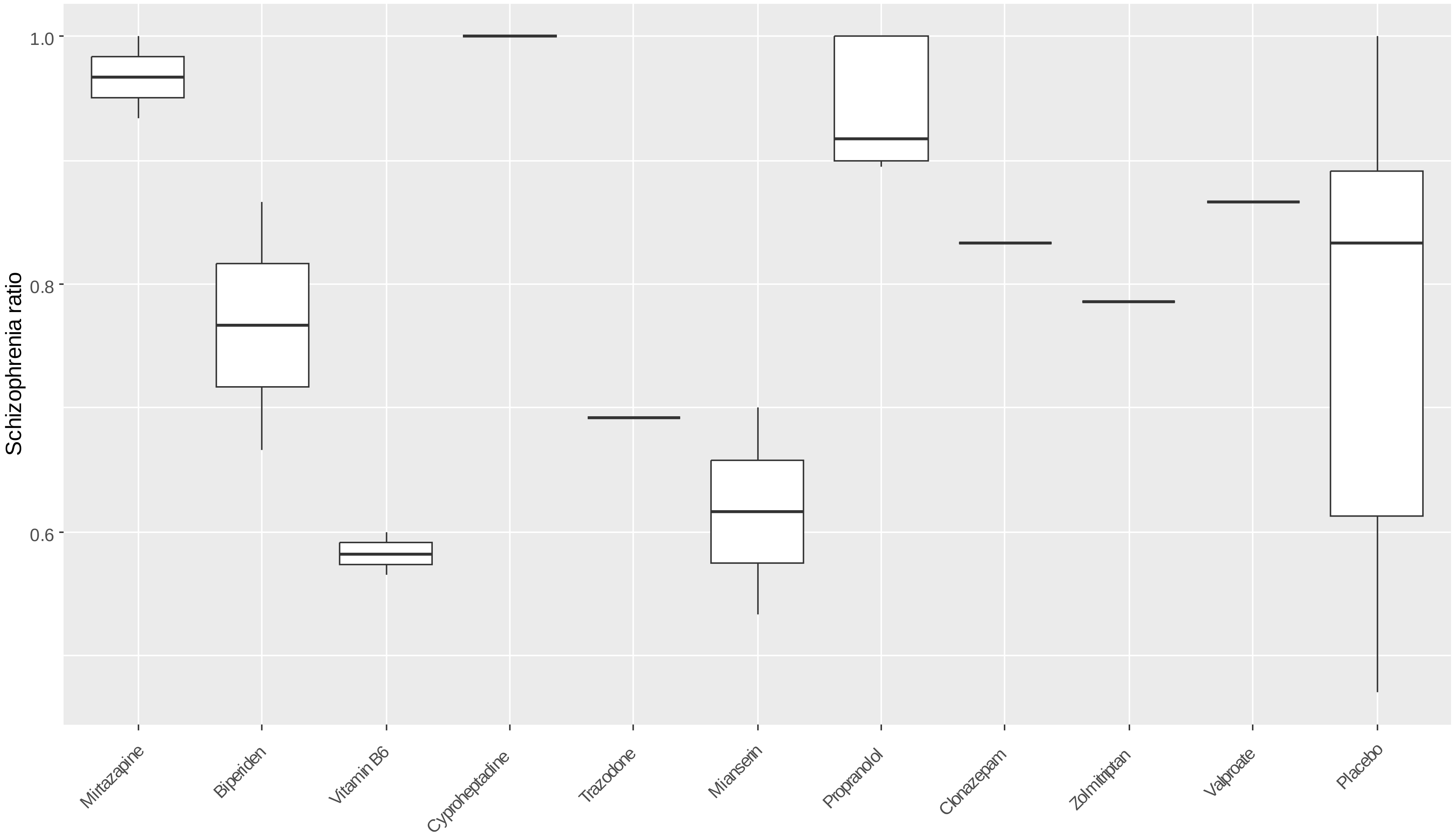

© 2024 Gerolymos C et al. *JAMA Network Open.*

eFigure 10. **Boxplot showing akathisia treatment duration (days) clustered by intervention.**

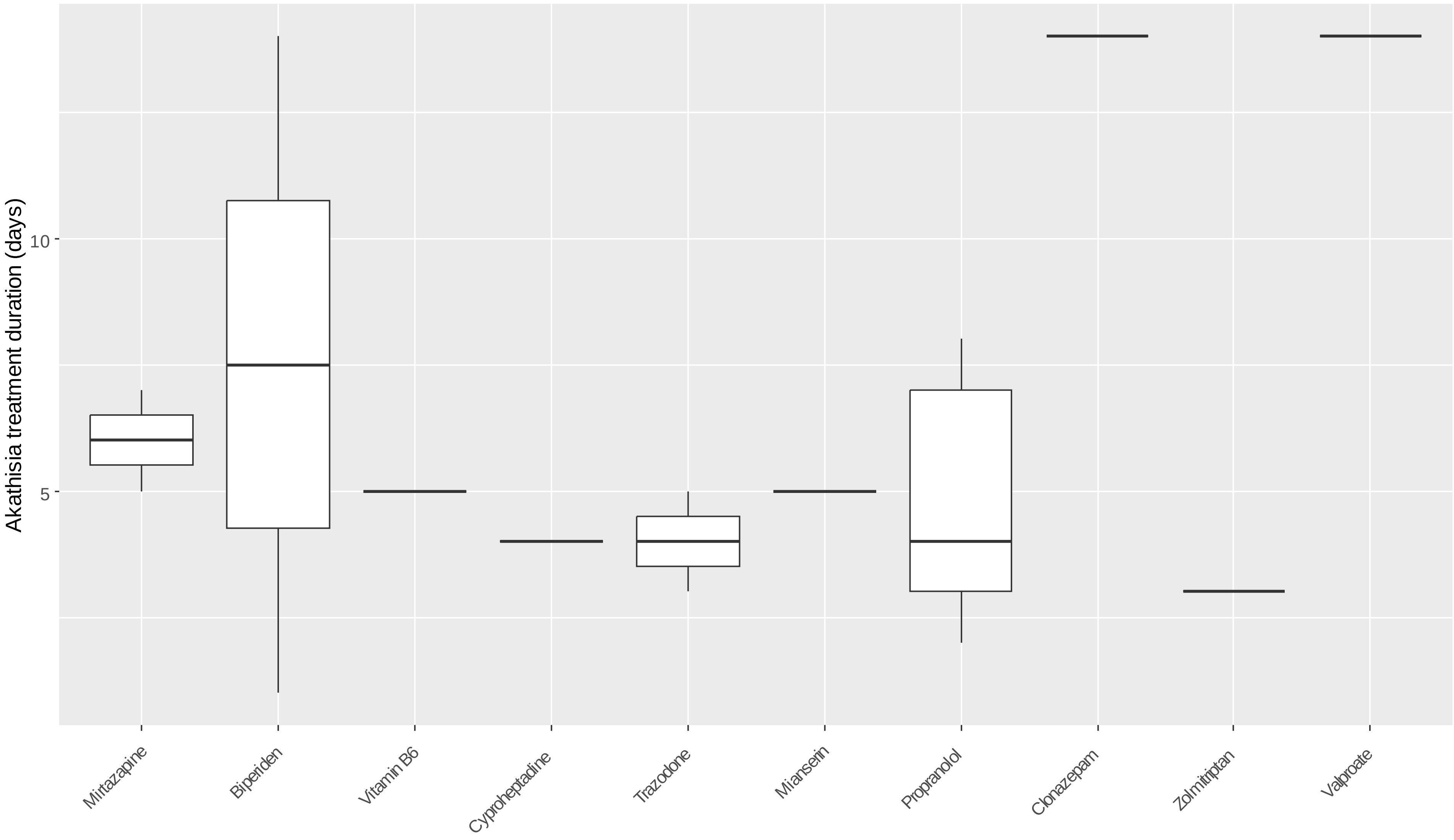

© 2024 Gerolymos C et al. *JAMA Network Open.*

eFigure 11. **Boxplot showing akathisia treatment dose (mg/day), sort by dose range (A – low dose) for each intervention.**

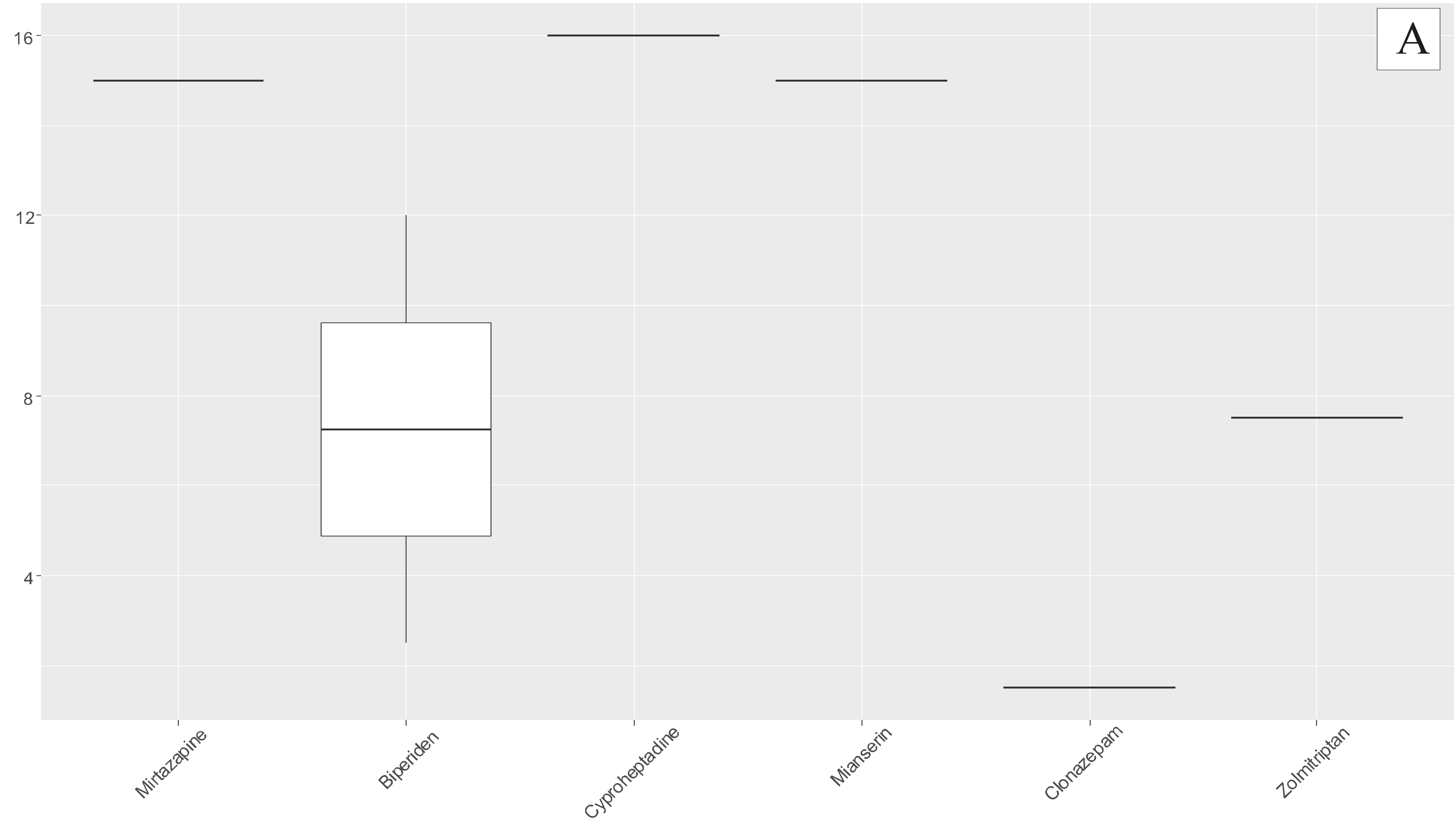

© 2024 Gerolymos C et al. *JAMA Network Open.*

eFigure 12. Boxplots showing akathisia treatment dose (mg/day), sort by dose range (B – intermediate dose, C – high) for each intervention.

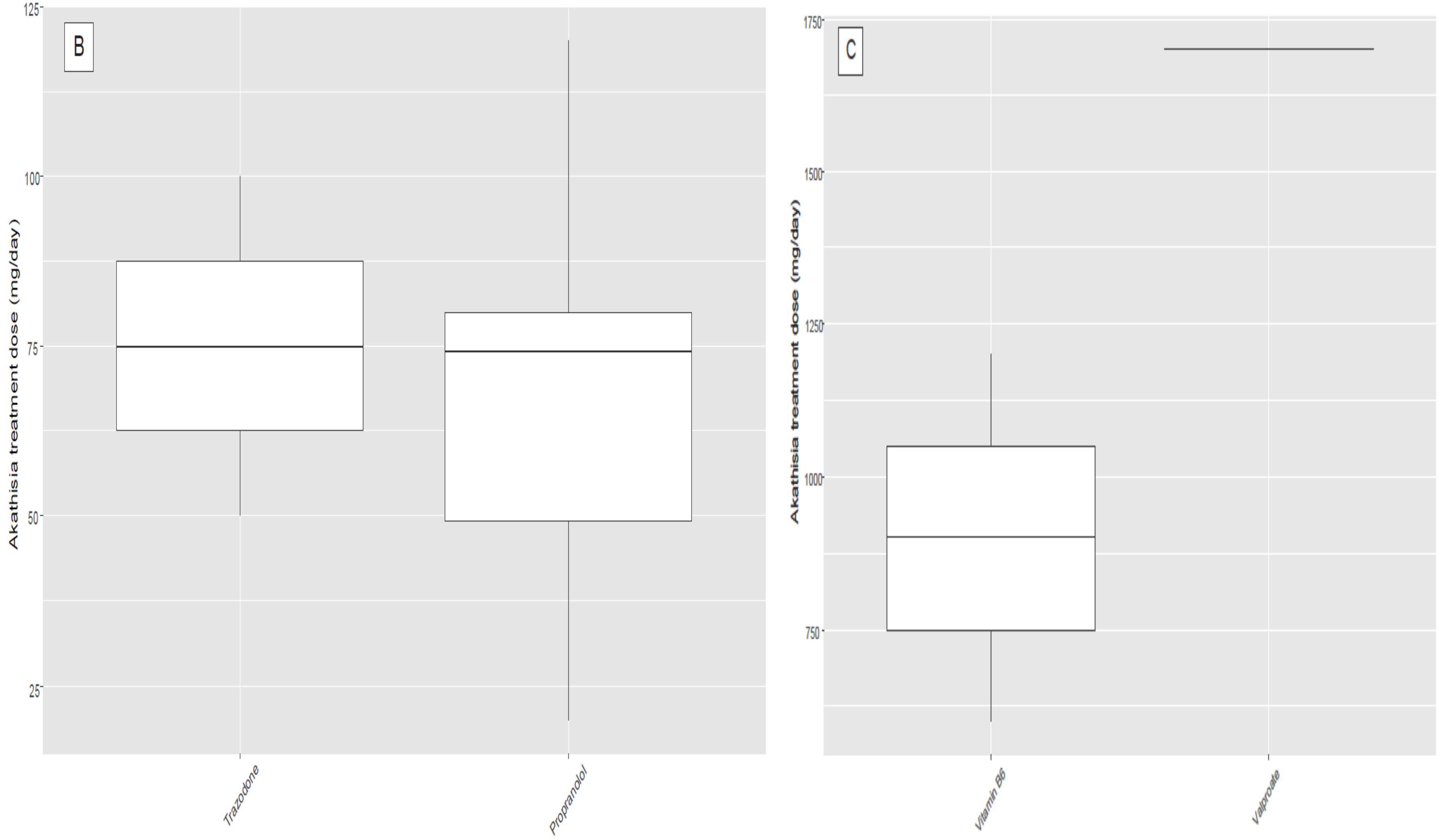

© 2024 Gerolymos C et al. *JAMA Network Open.*

eFigure 13. **Boxplot showing PANSS severity score at baseline (means) for each intervention.**

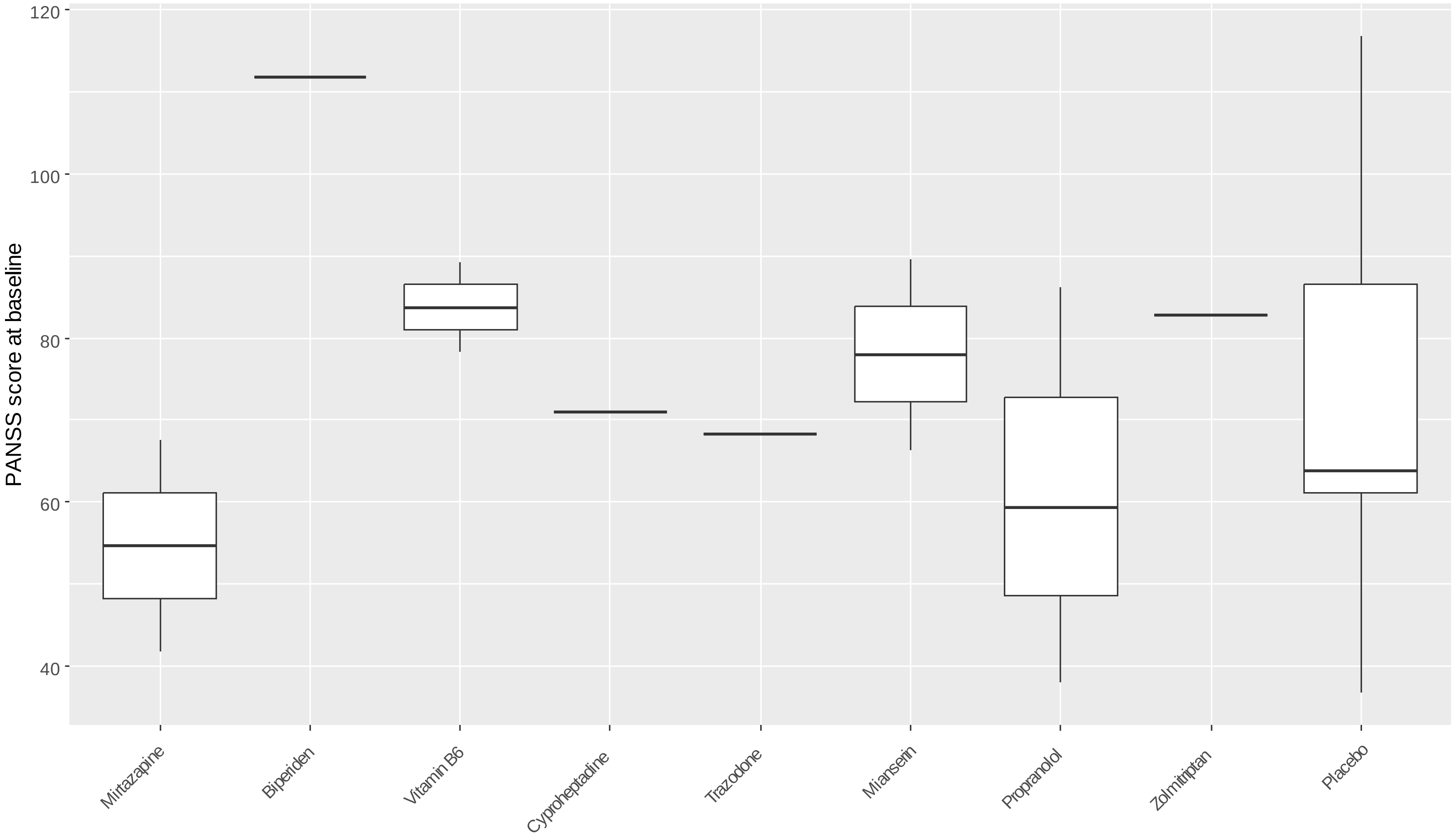

© 2024 Gerolymos C et al. *JAMA Network Open.*

eFigure 14. **Error bars showing the mean PANSS scores at baseline ± standard deviation for each intervention.**

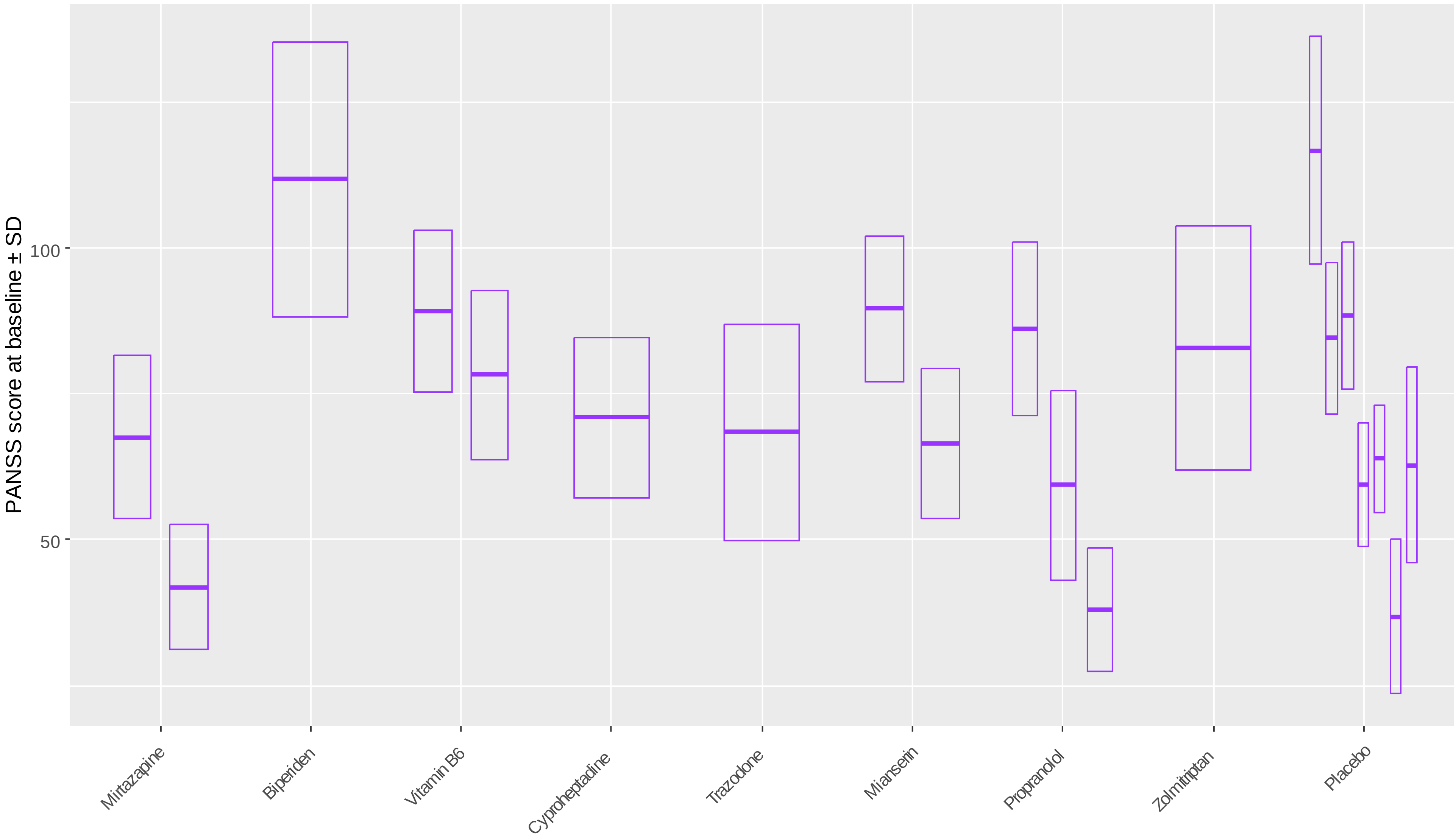

© 2024 Gerolymos C et al. *JAMA Network Open.*

eFigure 15. **Boxplot showing first-generation (typical) antipsychotic (FGA) ratio for each intervention.**

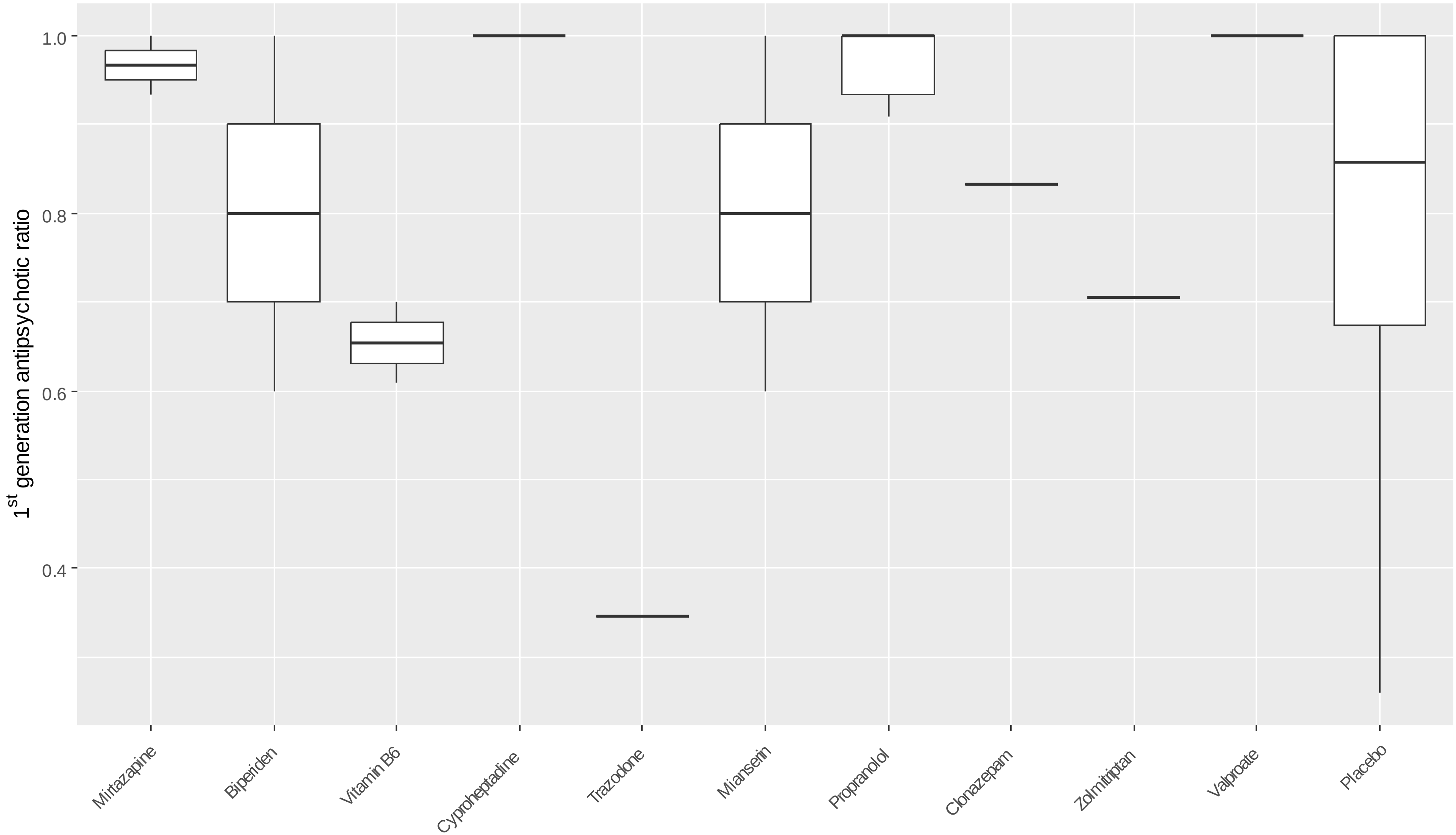

© 2024 Gerolymos C et al. *JAMA Network Open.*

eFigure 16. Boxplot showing chlorpromazine equivalent dose (mg/day) for each intervention.

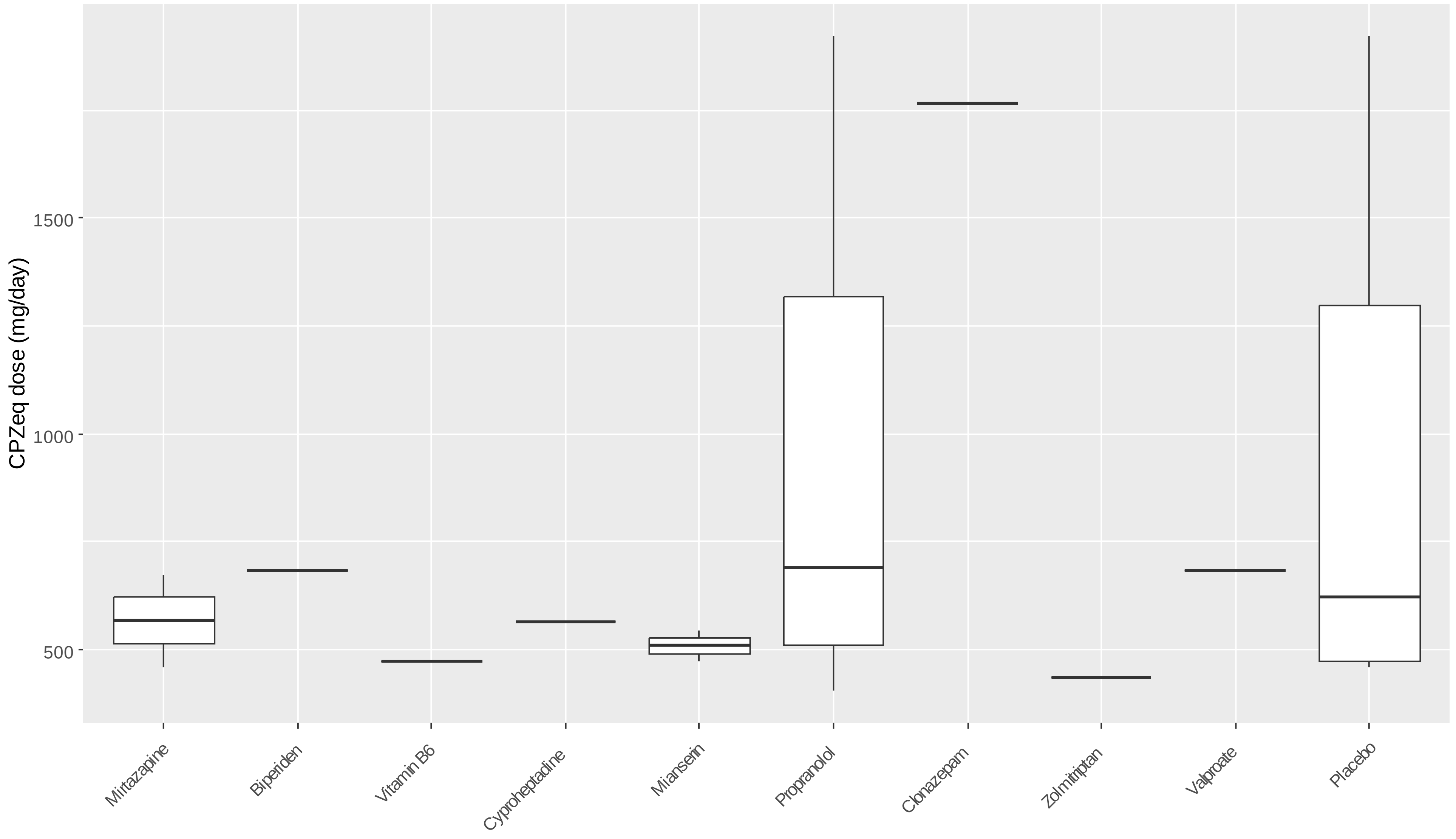

© 2024 Gerolymos C et al. *JAMA Network Open.*

eFigure 17. **Error bars showing mean chlorpromazine equivalent dose (mg/day) ± standard deviation for each intervention.**

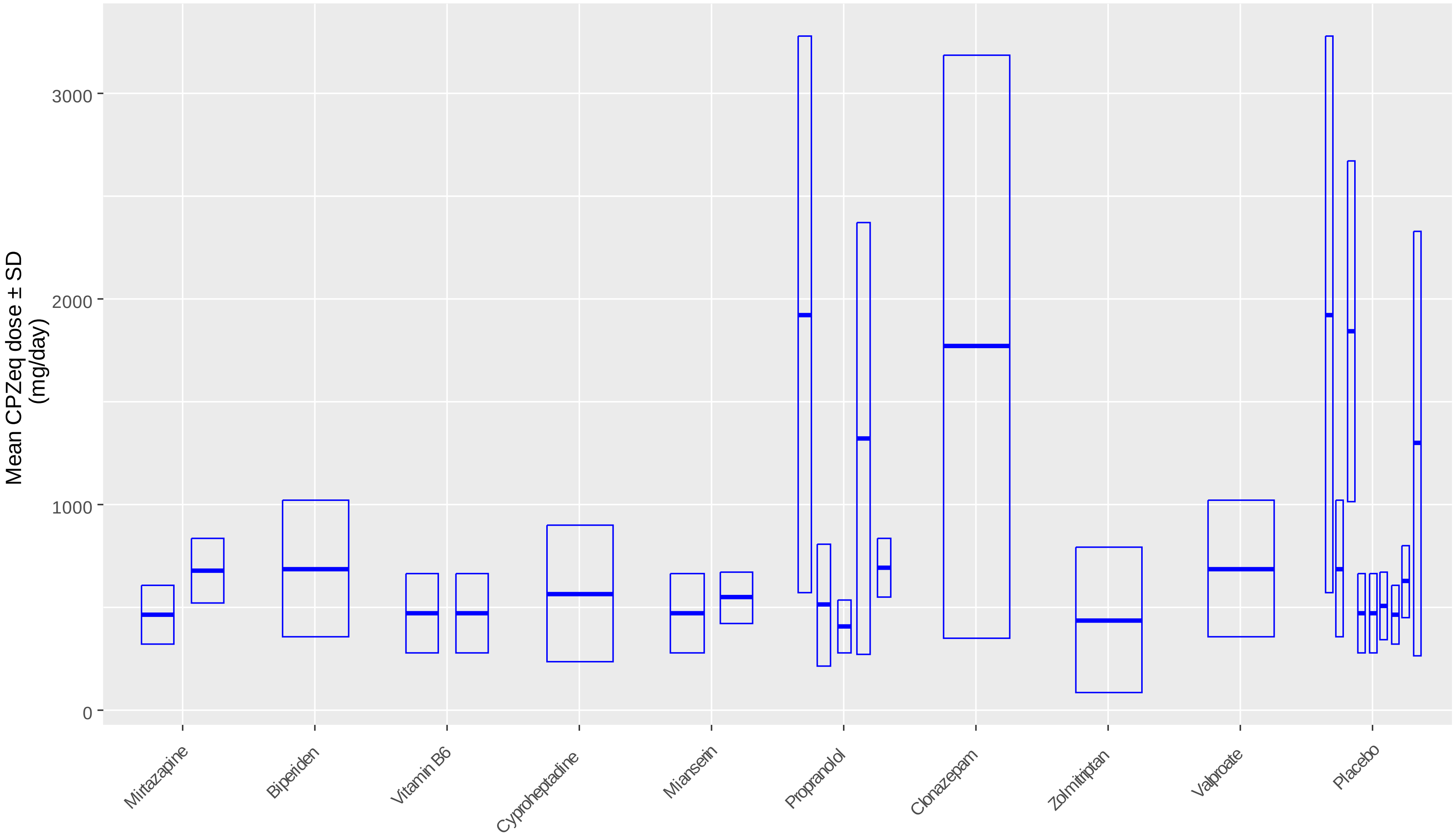

© 2024 Gerolymos C et al. *JAMA Network Open.*

eFigure 18. Boxplot showing ratio of patients treated with anticholinergic as cotreatment for each intervention.

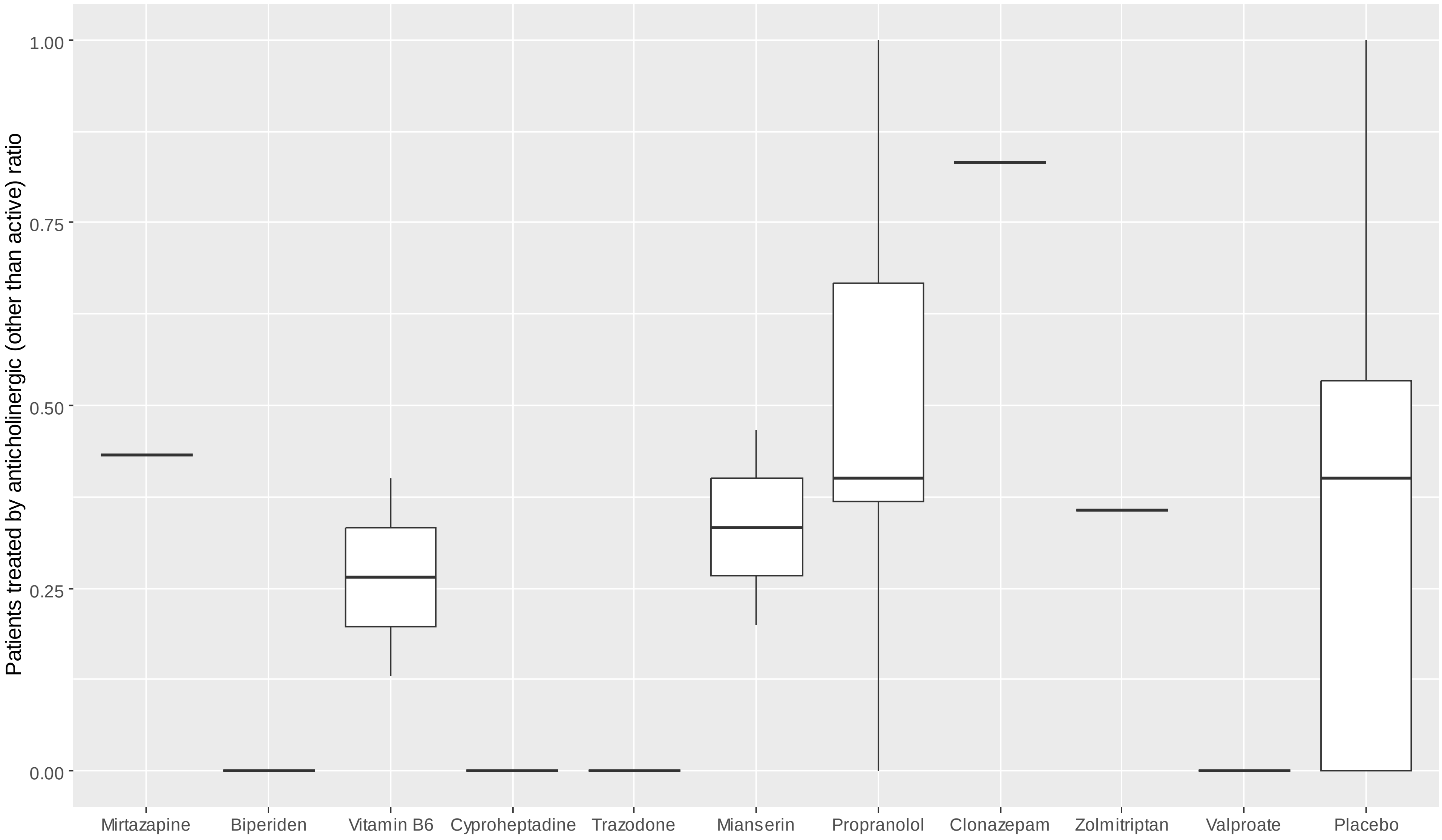

© 2024 Gerolymos C et al. *JAMA Network Open.*

eFigure 19. Error bars showing mean biperiden dose (cotreatment drug) (mg/day) ± standard deviation for each intervention.

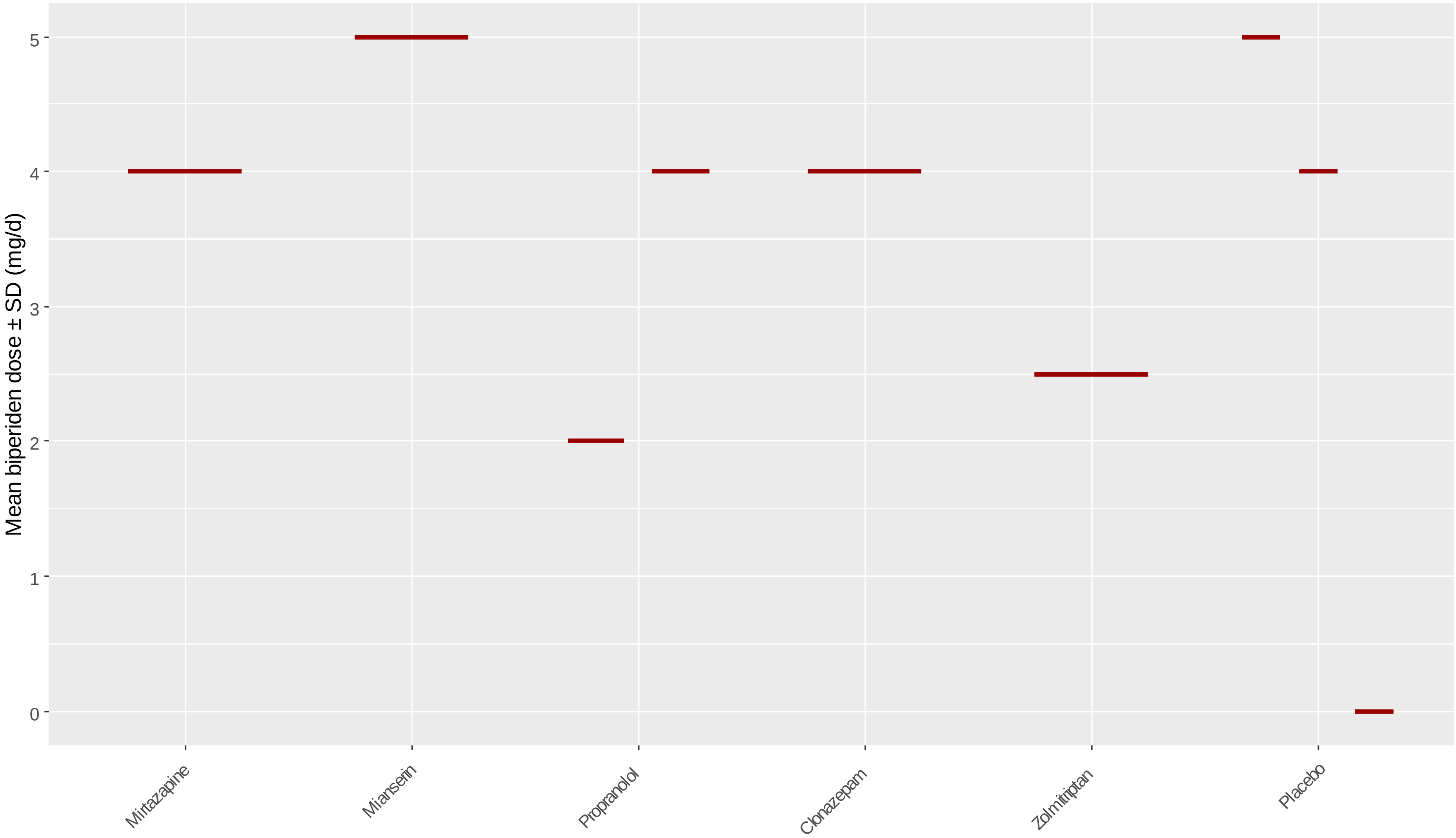

© 2024 Gerolymos C et al. *JAMA Network Open.*

eFigure 20. **Error bars showing mean trihexyphenidyl dose (mg/day) ± standard deviation for each intervention.**

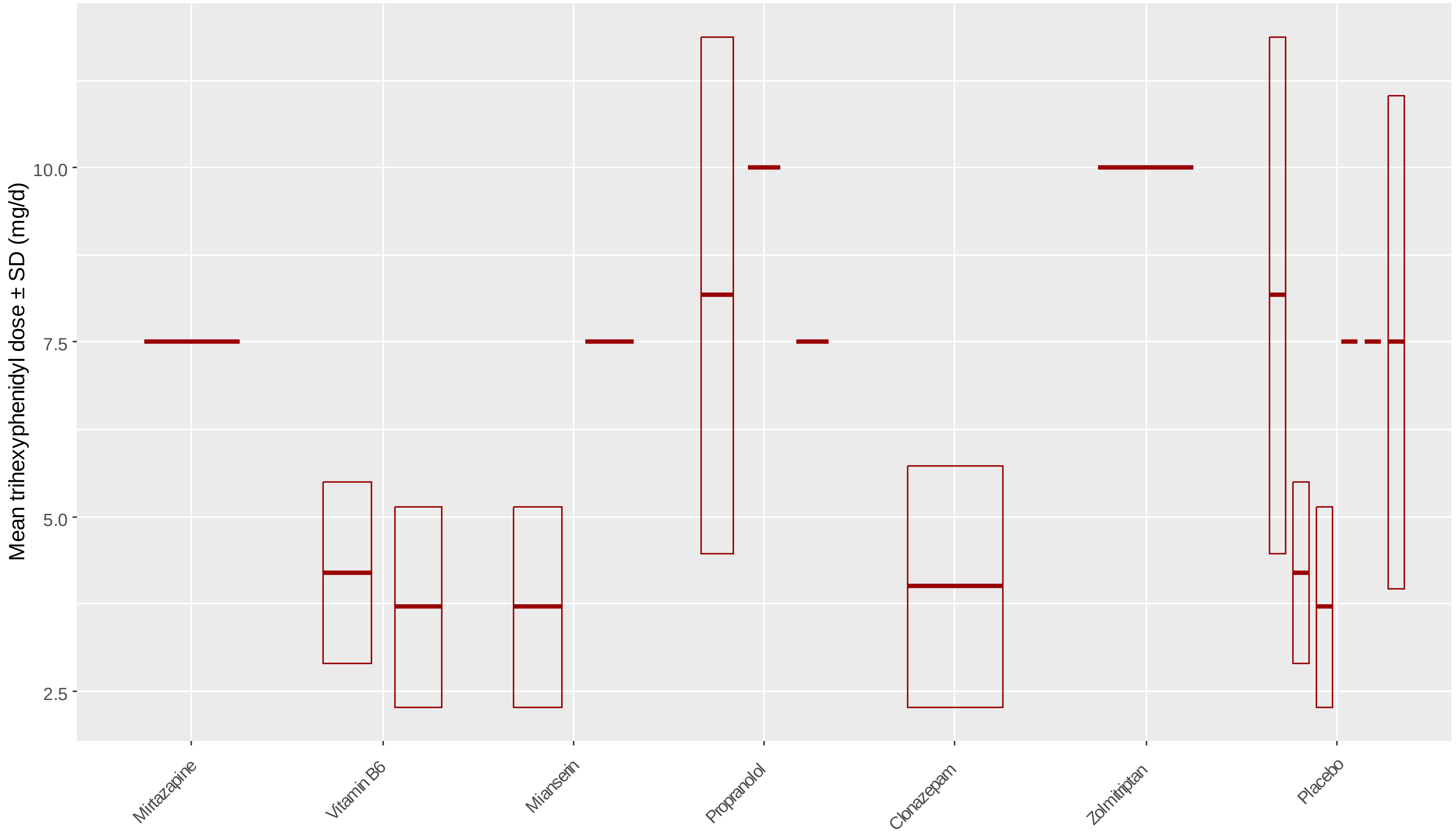

© 2024 Gerolymos C et al. *JAMA Network Open.*

eFigure 21. **Error bars showing mean benztropine dose (A), procyclidine dose (B) and tropatepine dose (C) (mg/day) ± standard deviation for each intervention.**

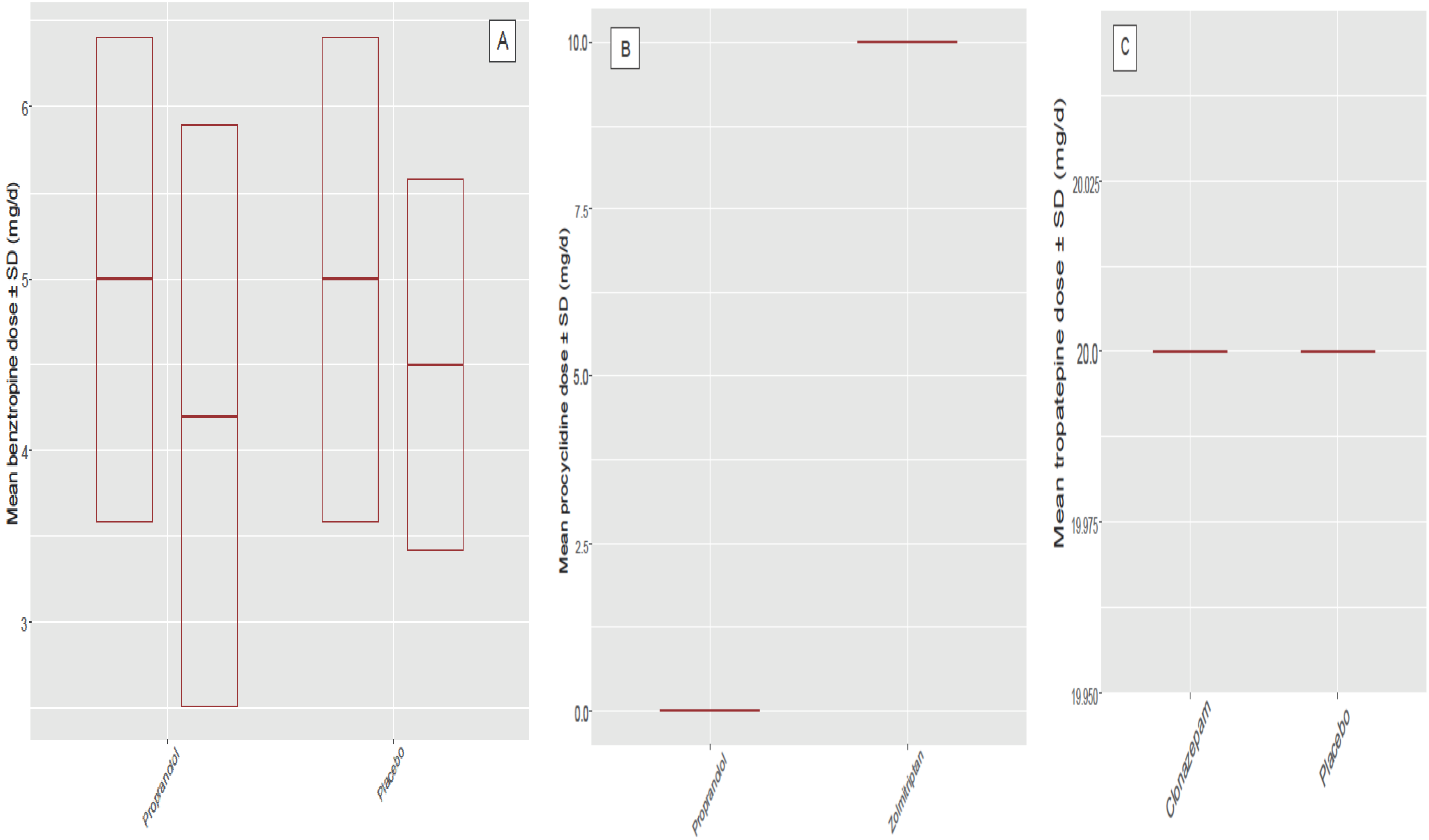

© 2024 Gerolymos C et al. *JAMA Network Open.*

eFigure 22. **Boxplot showing ratio of patients treated with benzodiazepine (cotreatment drug) for each intervention.**

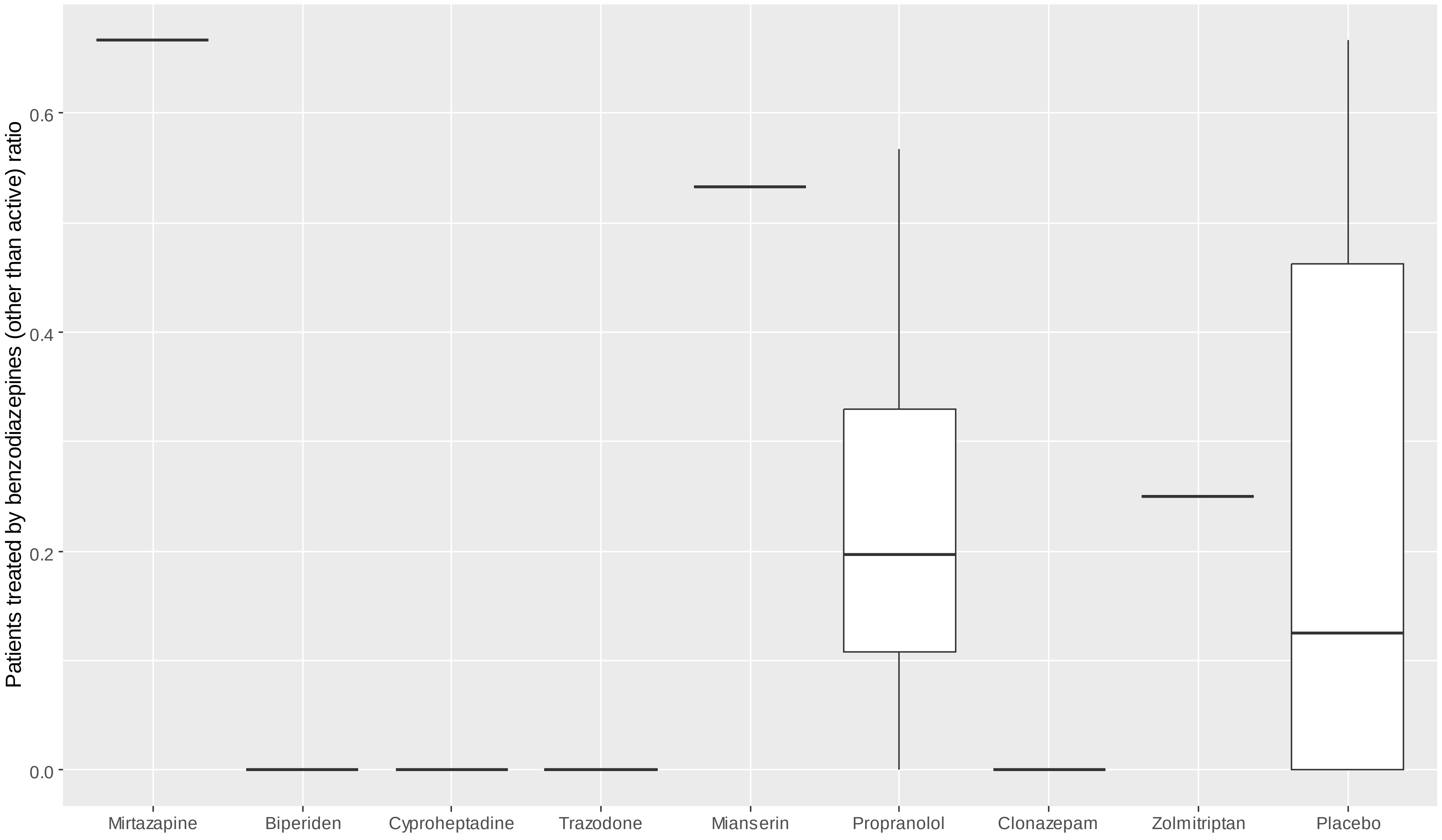

© 2024 Gerolymos C et al. *JAMA Network Open.*

eFigure 23. Error bars showing mean diazepam dose (cotreatment drug) (mg/day) ± standard deviation for each intervention.

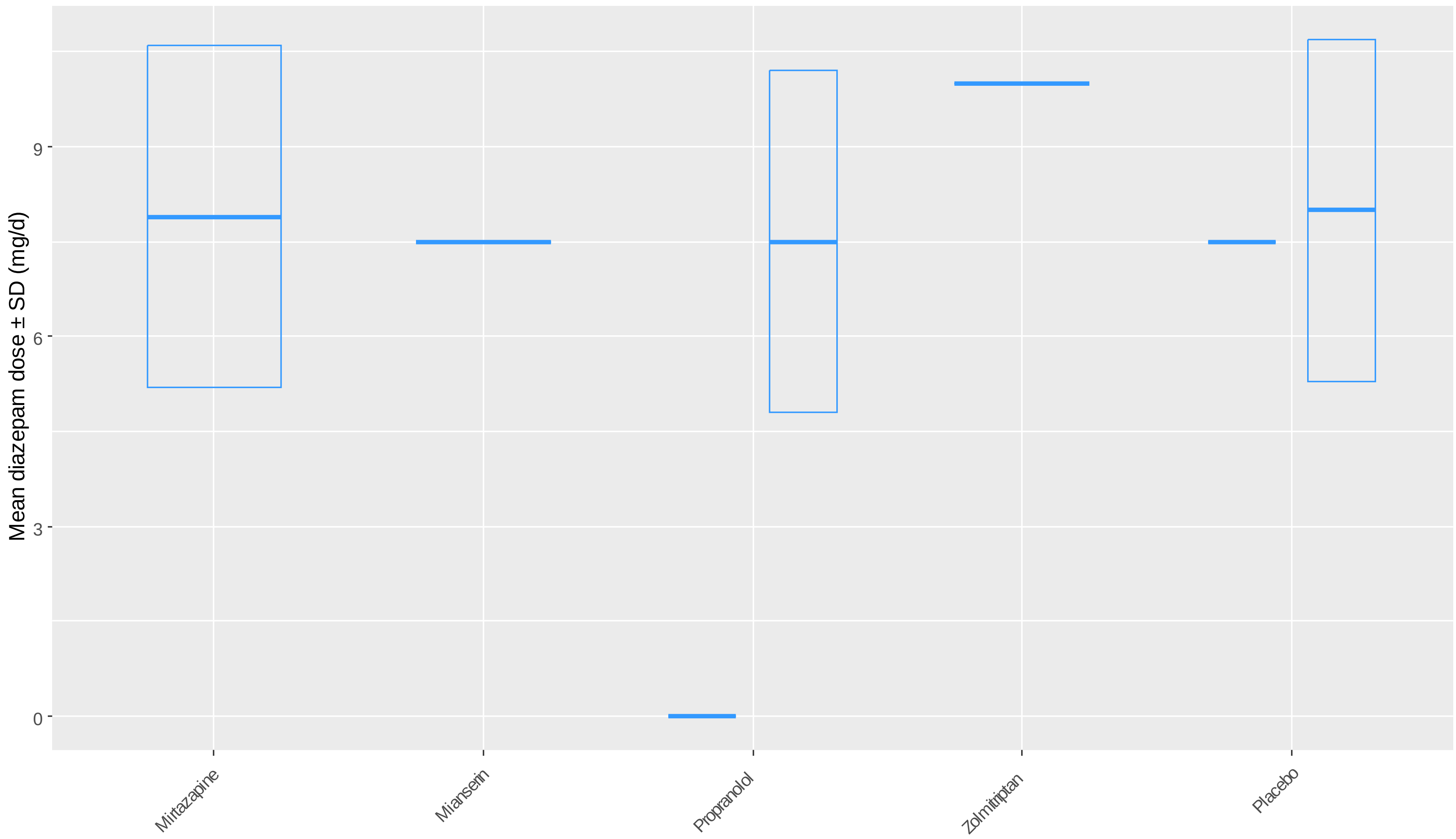

© 2024 Gerolymos C et al. *JAMA Network Open.*

eFigure 24. **Error bars showing mean lorazepam dose (mg/day) ± standard deviation for each intervention.**

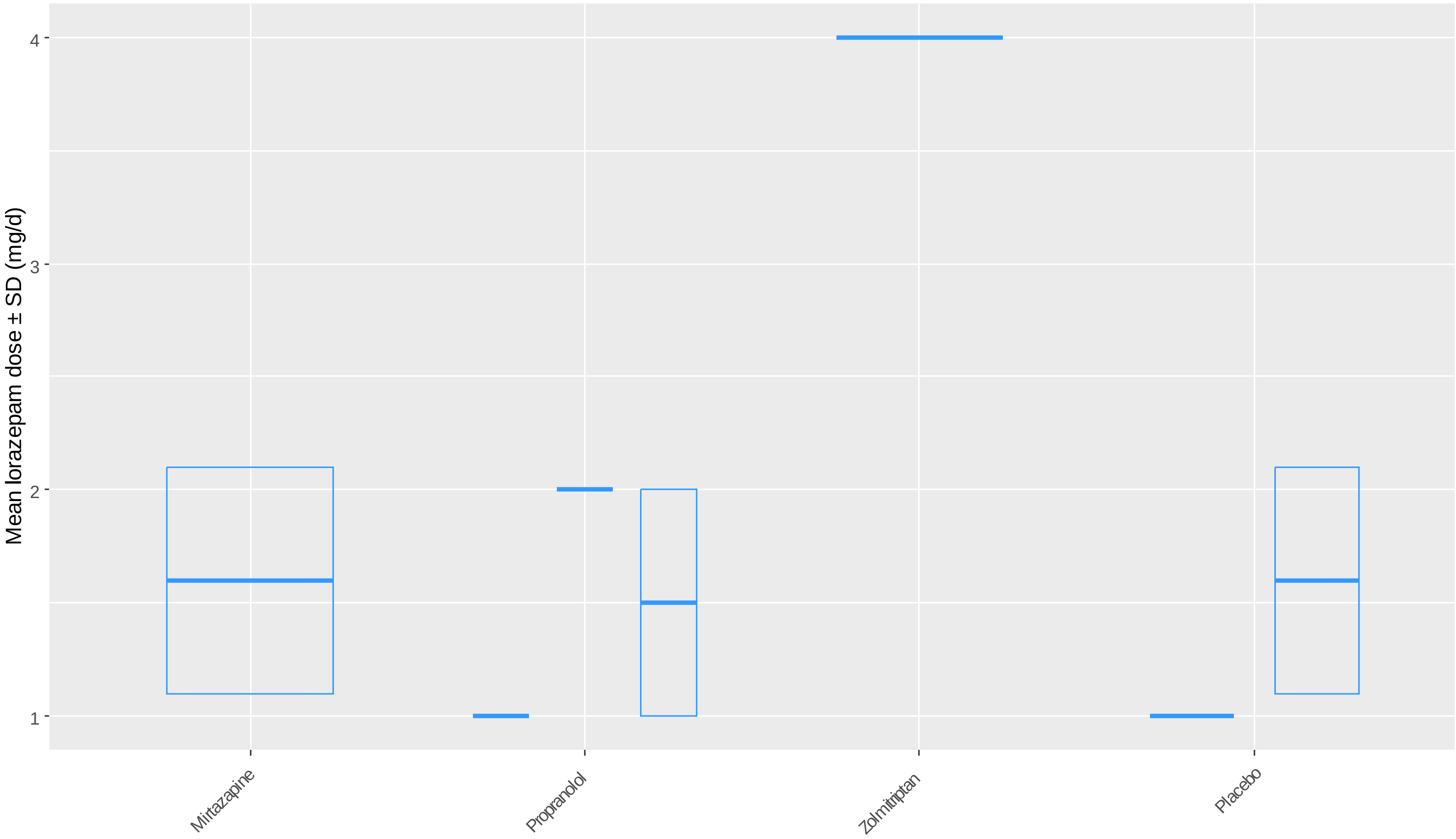

© 2024 Gerolymos C et al. *JAMA Network Open.*

eFigure 25. **Error bars showing mean nitrazepam dose (A), clonazepam dose (B) (cotreatment drug) and temazepam dose (C) (mg/day) ± standard deviation for each intervention.**

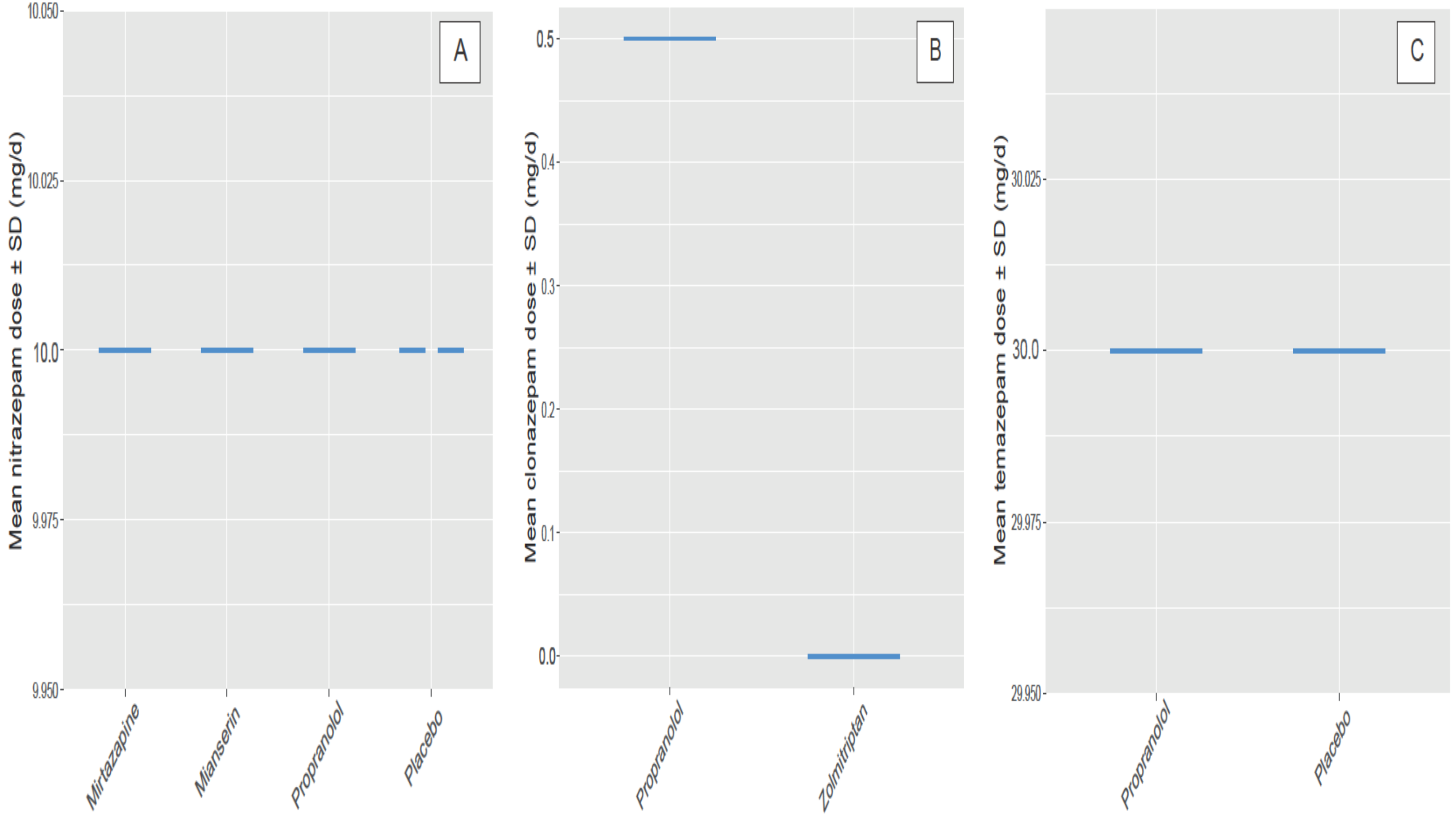

© 2024 Gerolymos C et al. *JAMA Network Open.*

eFigure 26. **Boxplot showing mean Simpson Angus Scale (SAS) scores at baseline for each intervention.**

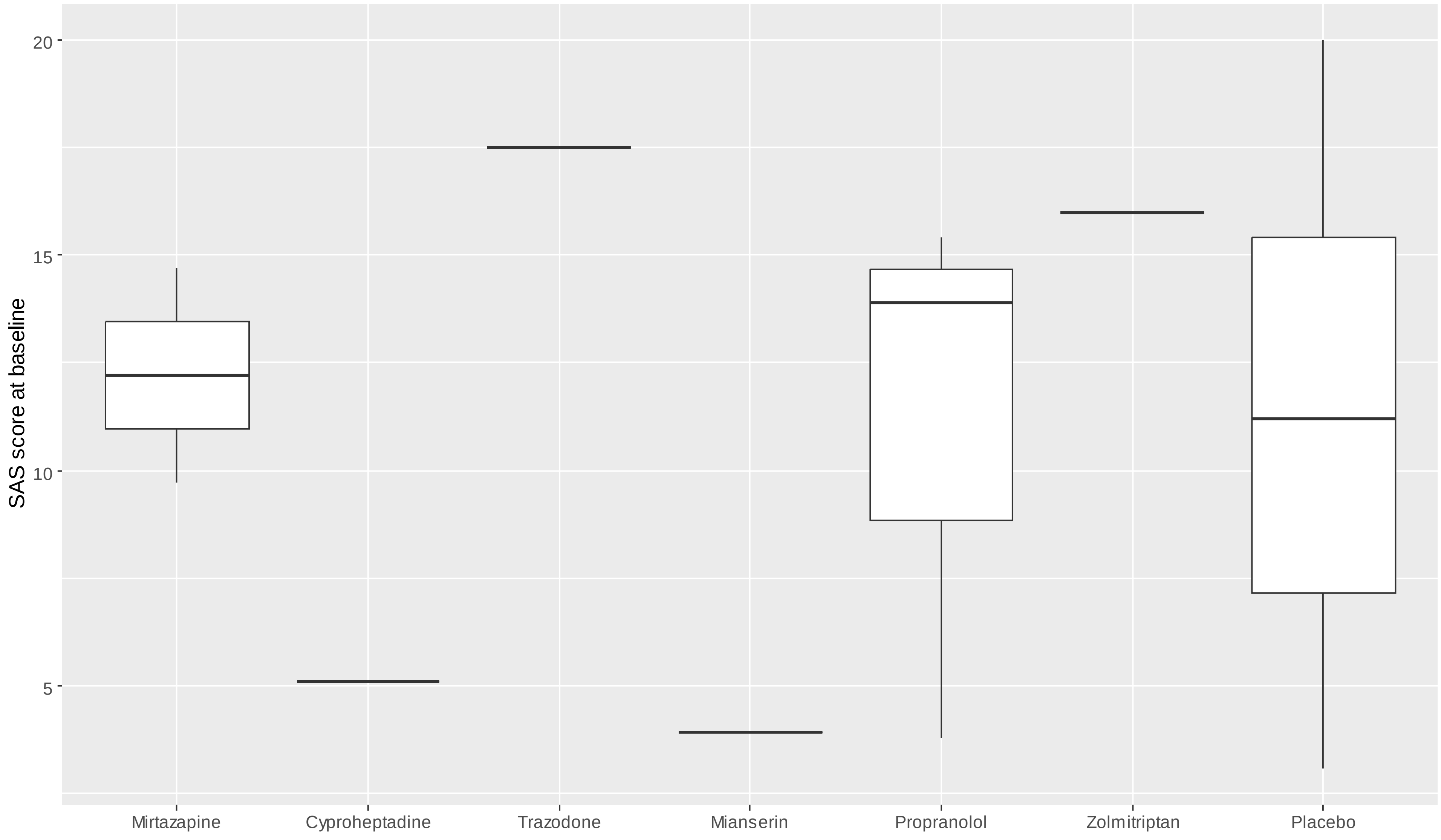

© 2024 Gerolymos C et al. *JAMA Network Open.*

eFigure 27. **Error bars showing mean SAS scores at baseline ± standard deviation for each intervention.**

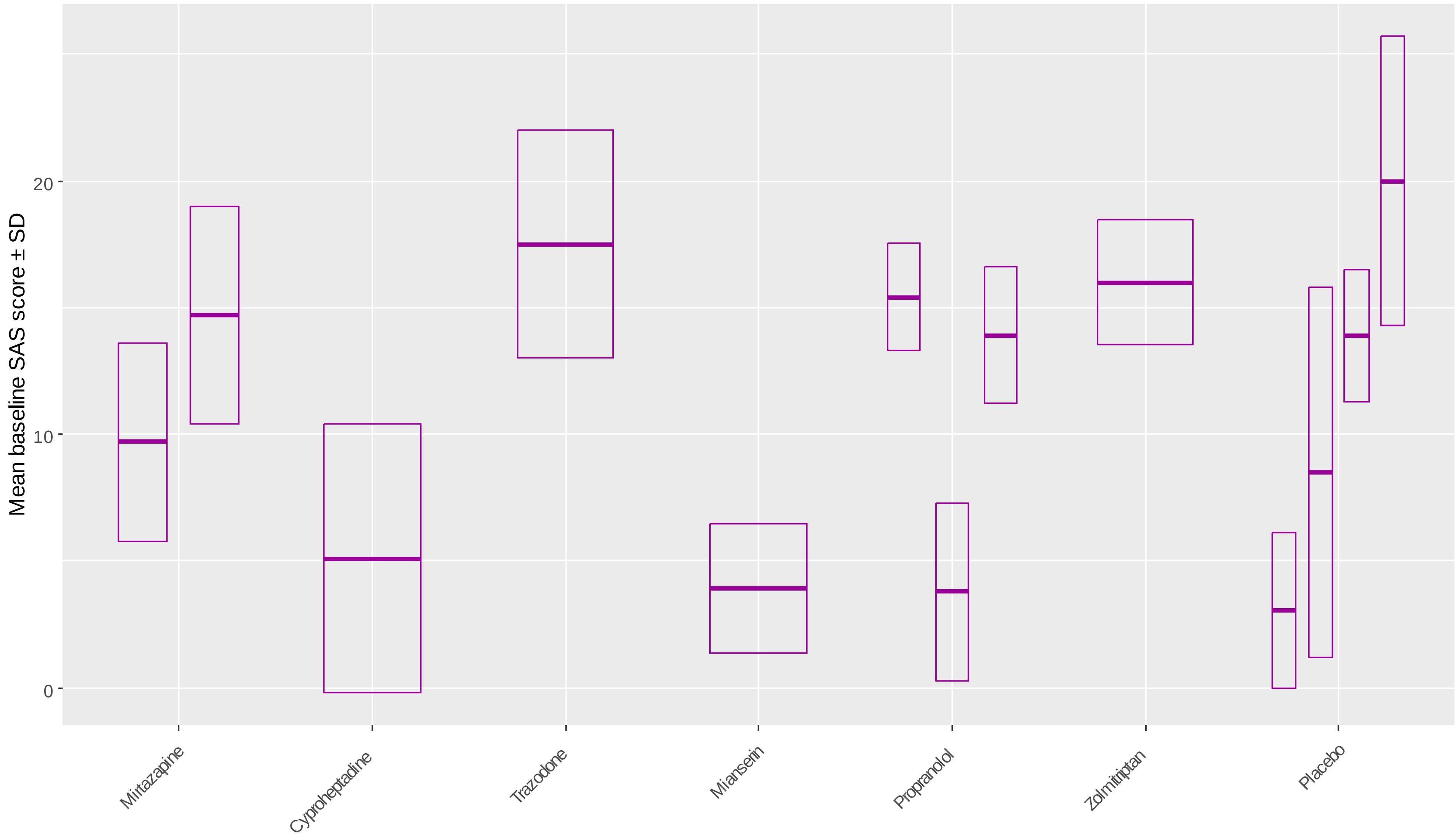

© 2024 Gerolymos C et al. *JAMA Network Open.*

eFigure 28. **Boxplot showing mean Hamilton Depression Rating Scale (HAM-D) scores at baseline for each intervention.**

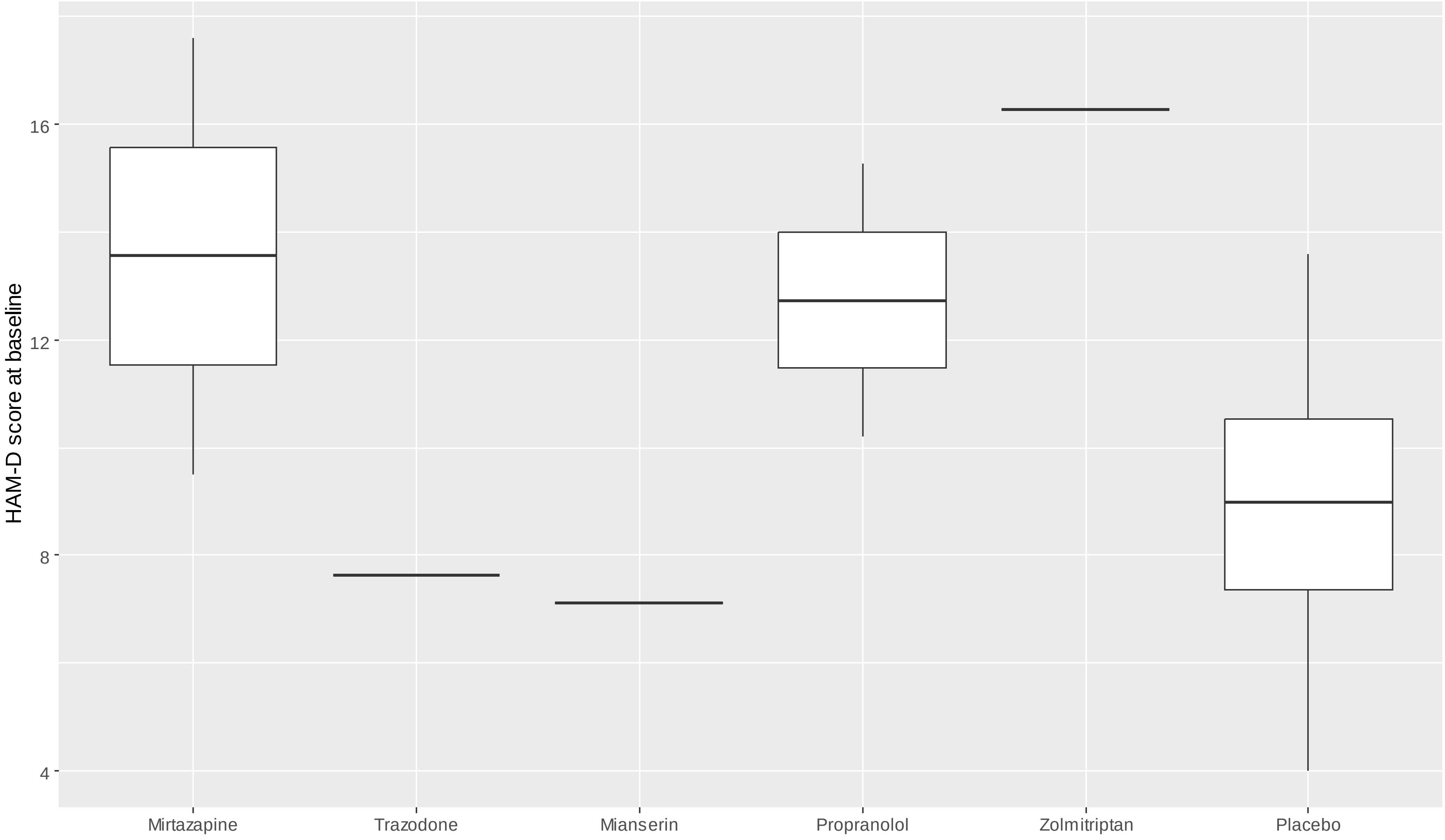

© 2024 Gerolymos C et al. *JAMA Network Open.*

eFigure 29. **Error bars showing mean HAM-D scores at baseline ± standard deviation for each intervention.**

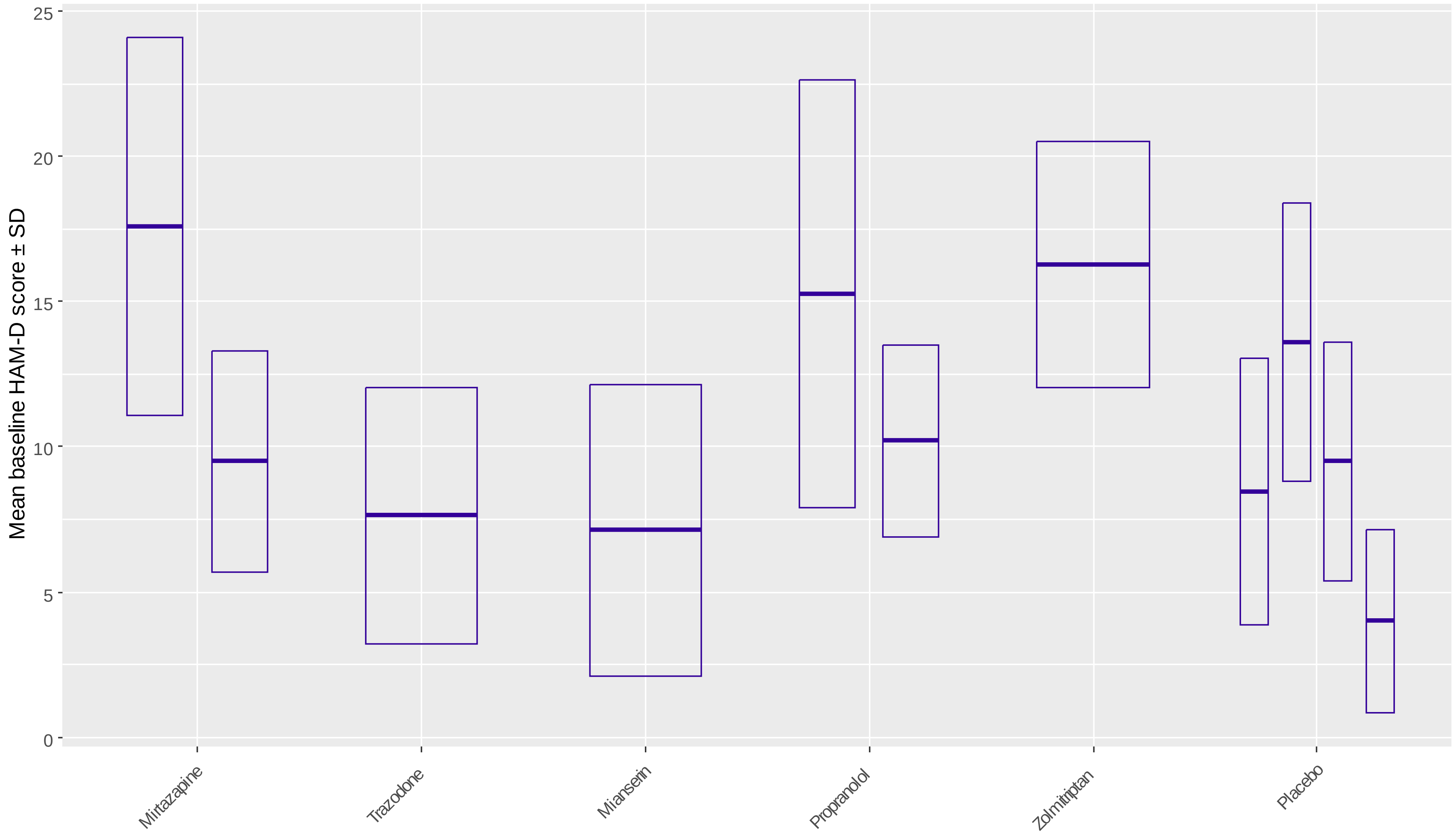

© 2024 Gerolymos C et al. *JAMA Network Open.*

4. Sensitivity analyses

3.1. Effect size modifiers analyses

To examine the existence of potential effect size modifiers, we present below error bars, representing standardized mean differences (SMD) ± standard error of standardized mean differences (seSMD), colored according to the parameter explored.

A gradient scale supports each figure to visualize whether there is an impact of the parameter.

When the comparison considered does not use placebo as control group, a legend precise the active chosen as reference group.

For akathisia treatment dose: the interventions are separated in three categories to clearly visualize respective doses.

Effect size modifiers analyses

We investigated potential effect size modifiers. The efficacy of biperiden and propranolol increased with treatment duration. The efficacy of biperiden, propranolol, and trazodone increased with treatment dose. In one randomized controlled trial, co-treatment with biperiden 4 mg/day was administered in the clonazepam group for one patient(Pujalte et al. 1994) and not in placebo group. Otherwise, no relationship was found between the dose of benzodiazepines and anticholinergics as cotreatment and the significance of SMDs. No significant association was found between effect sizes and psychotic severity (measured using PANSS score). There was no relationship between baseline SAS score, baseline HAM-D score and significance of effect sizes. Higher chlorpromazine equivalent doses were found in the clonazepam arm, whose effect size wasn't significant, and in one propranolol arm, which effect size remained significant.

© 2024 Gerolymos C et al. *JAMA Network Open.*

eFigure 30. Error bars showing the different standardized mean differences ± standard deviation for each intervention according to akathisia treatment duration (in days).

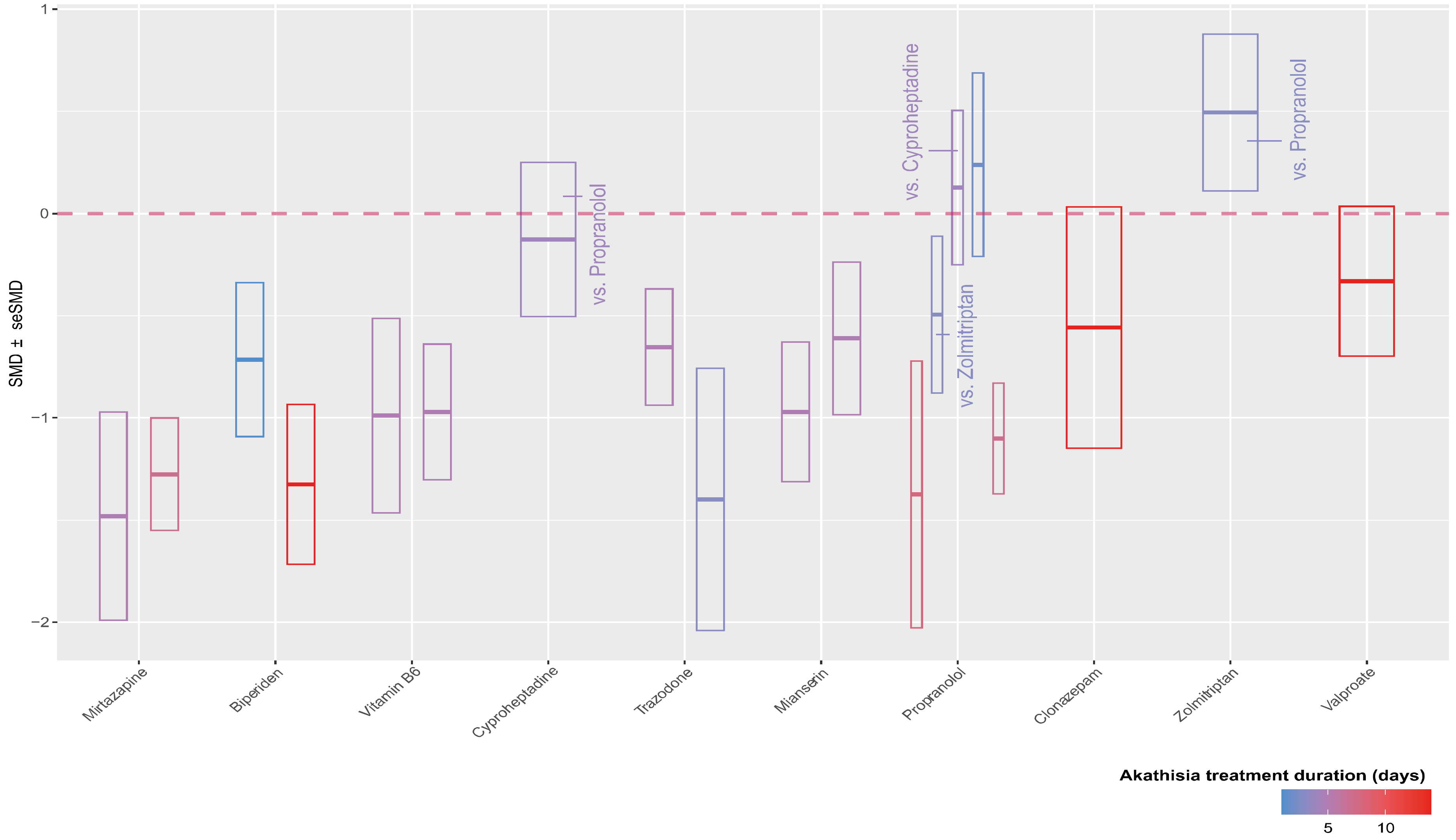

© 2024 Gerolymos C et al. *JAMA Network Open.*

eFigure 31. Error bars showing the different standardized mean differences ± standard deviation for each intervention, sort by dose range (A – low dose), according to akathisia treatment dose (mg/day).

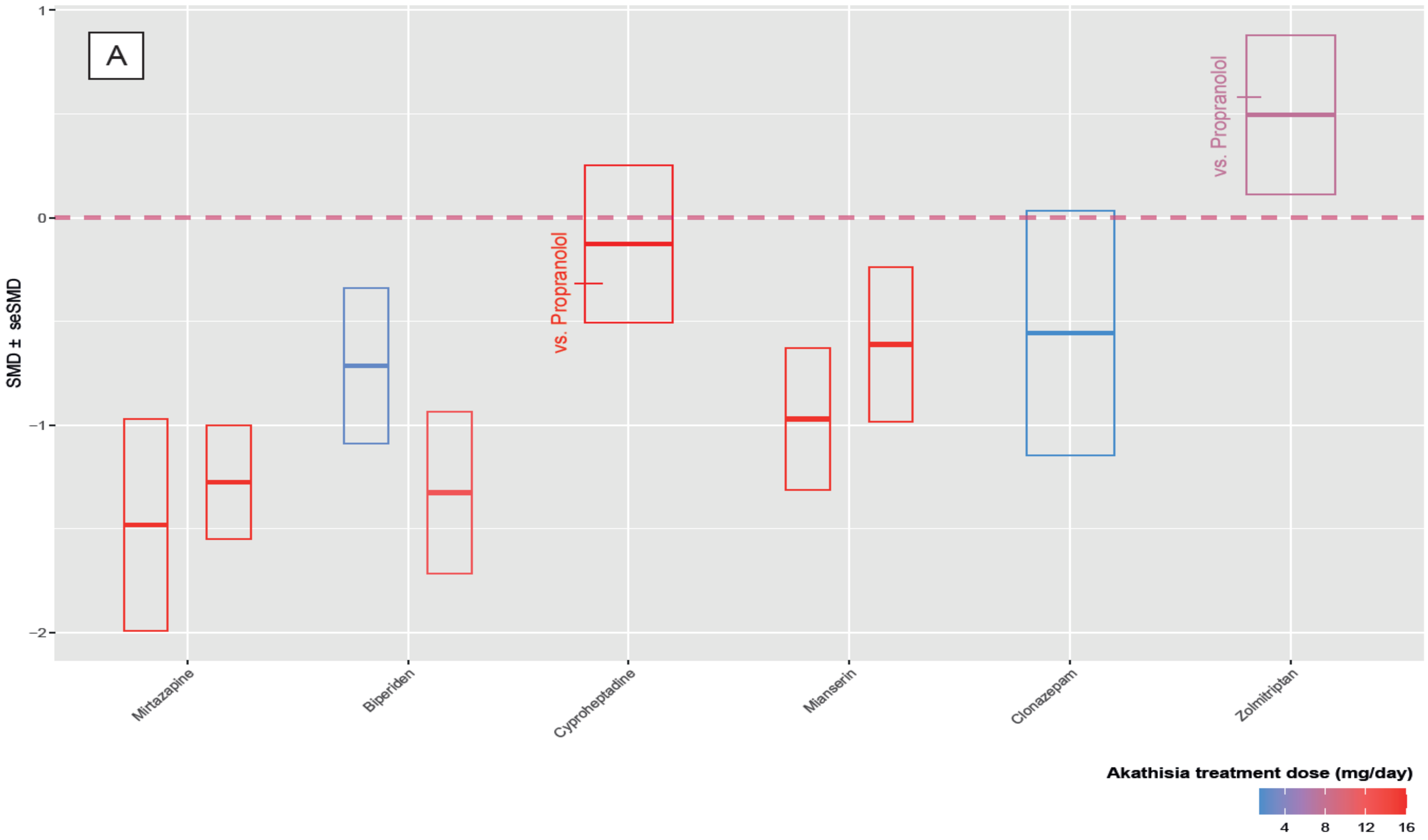

© 2024 Gerolymos C et al. *JAMA Network Open.*

eFigure 32. Error bars showing the different standardized mean differences ± standard deviation for each intervention, sort by dose range (B – intermediate dose, C – high dose), according to akathisia treatment dose (mg/day).

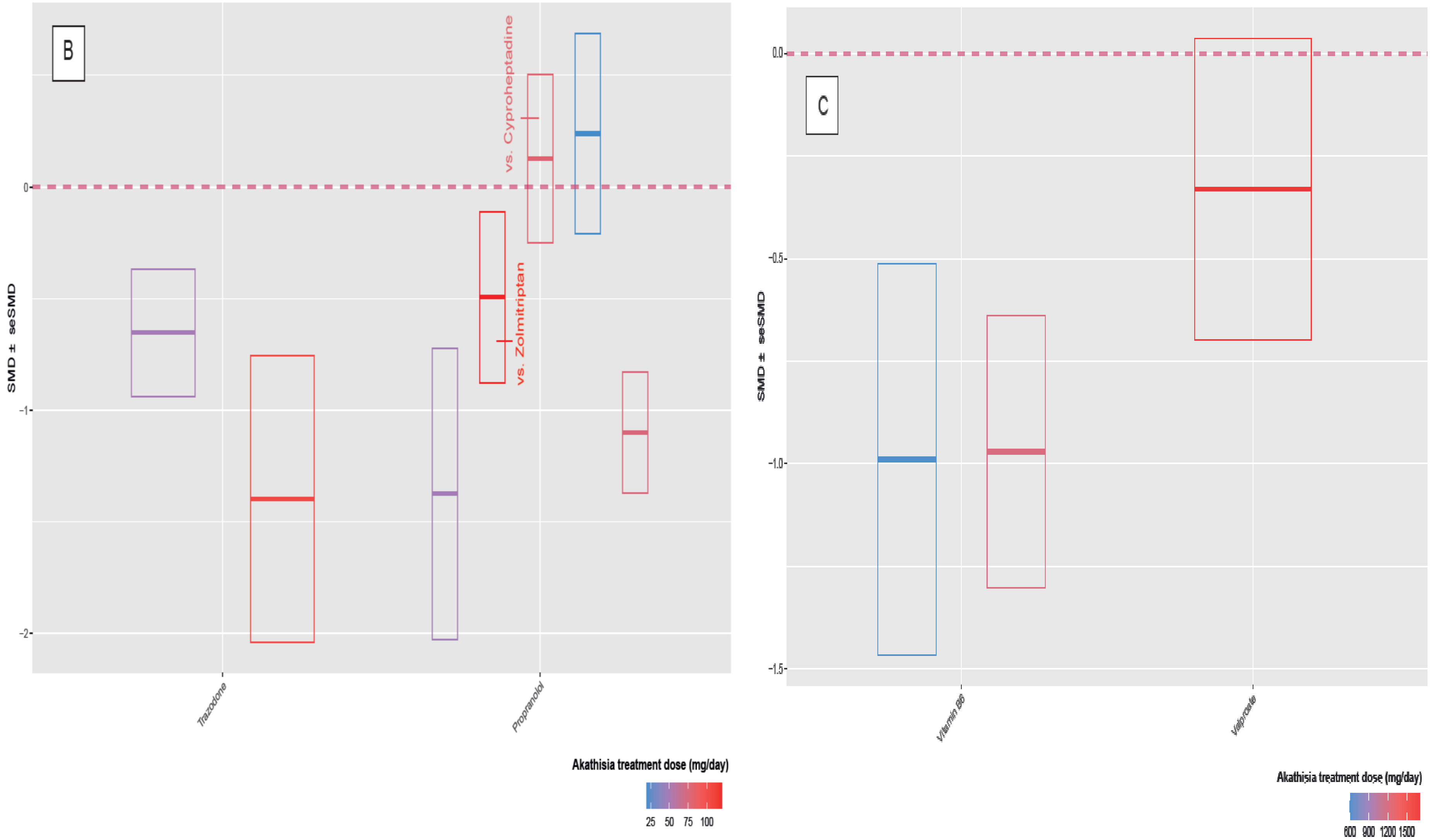

© 2024 Gerolymos C et al. *JAMA Network Open.*

eFigure 33. **Error bars showing the different standardized mean differences ± standard deviation for each intervention according to PANSS score (mean).**

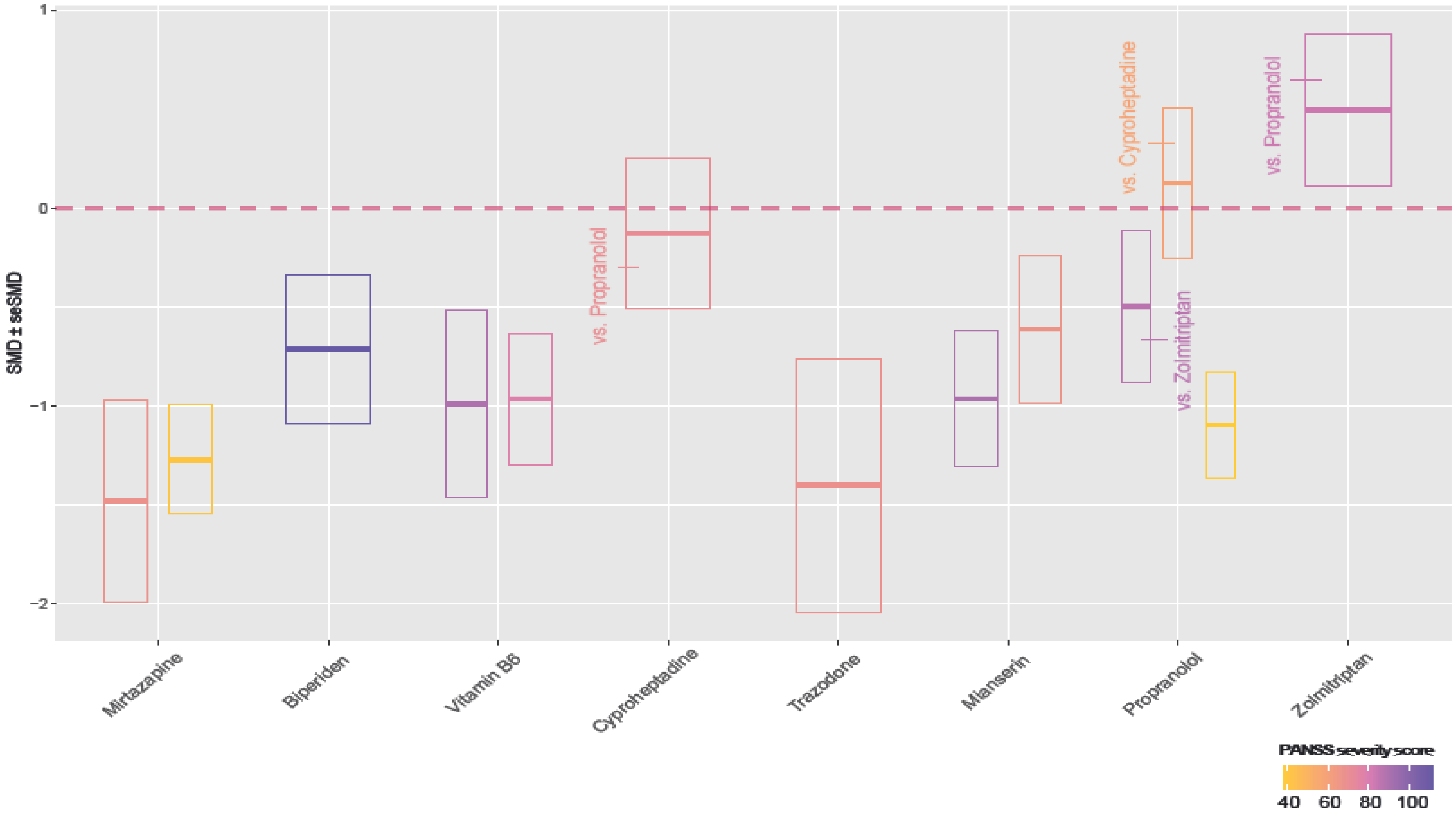

© 2024 Gerolymos C et al. *JAMA Network Open.*

eFigure 34. Error bars showing the different standardized mean differences ± standard deviation for each intervention according to chlorpromazine equivalent dose (mg/day).

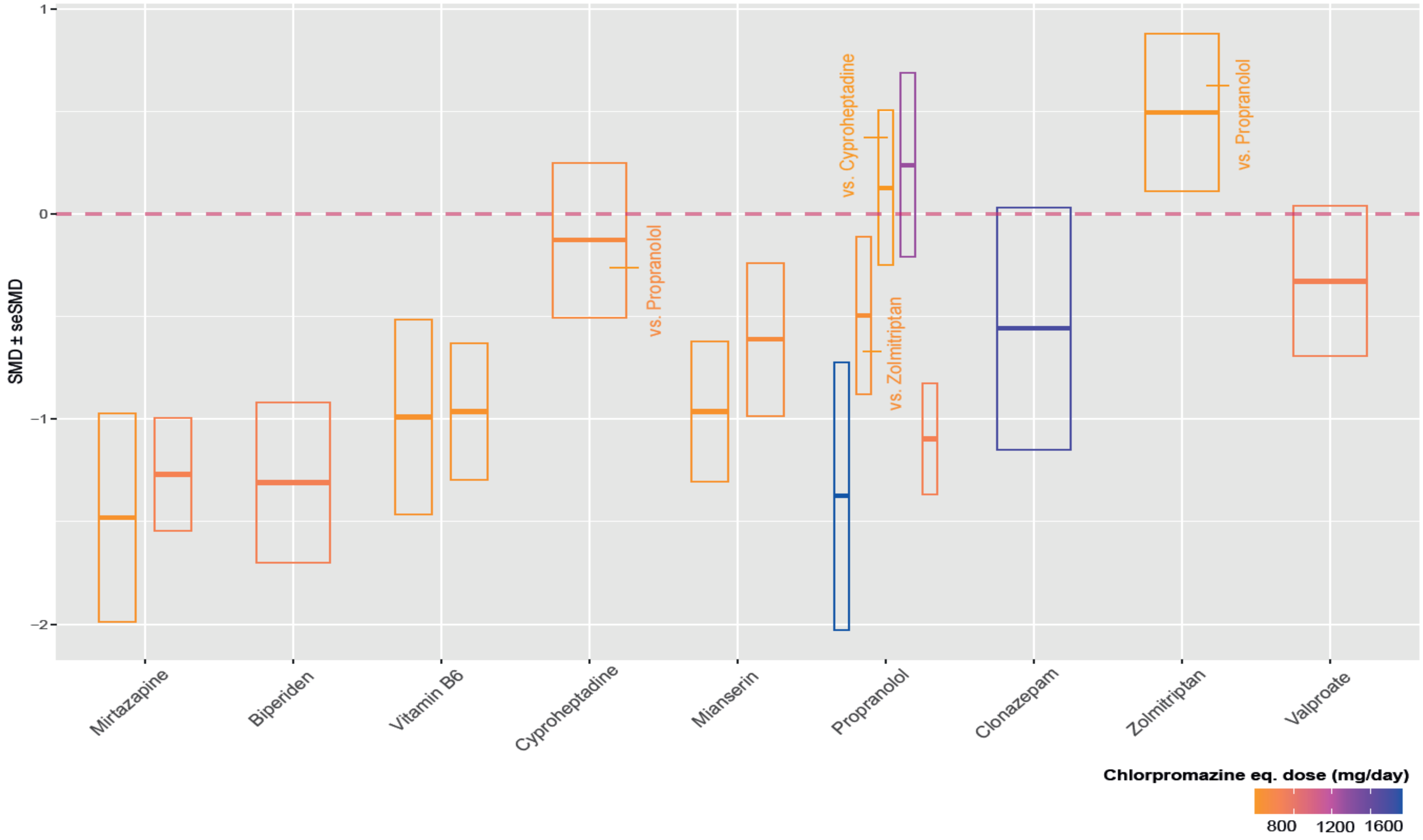

© 2024 Gerolymos C et al. *JAMA Network Open.*

eFigure 35. **Error bars showing the different standardized mean differences ± standard deviation for each intervention according to biperiden mean dose (mg/day), used as cotreatment drug.**

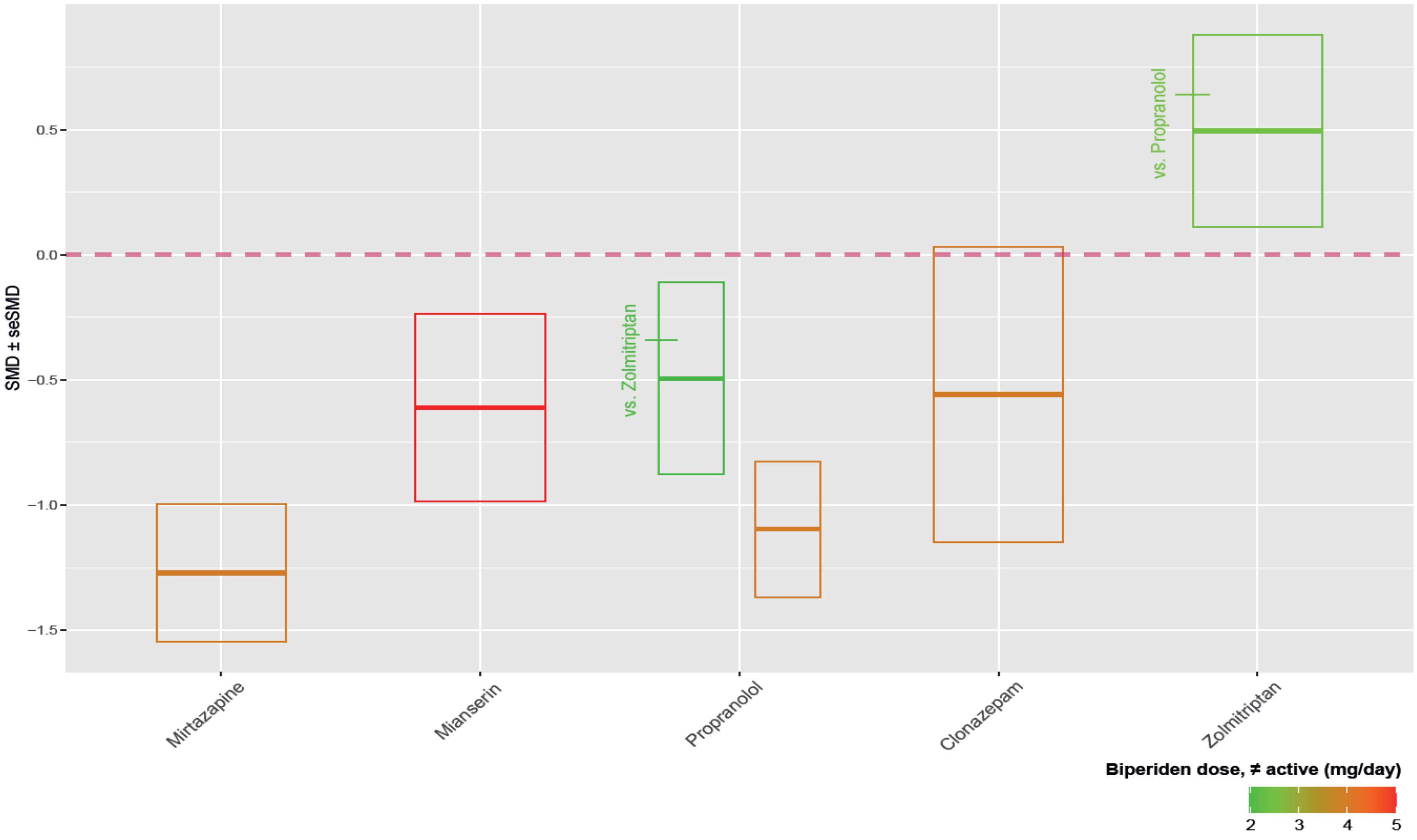

© 2024 Gerolymos C et al. *JAMA Network Open.*

eFigure 36. Error bars showing the different standardized mean differences ± standard deviation for each intervention according to trihexyphenidyl mean dose (mg/day).

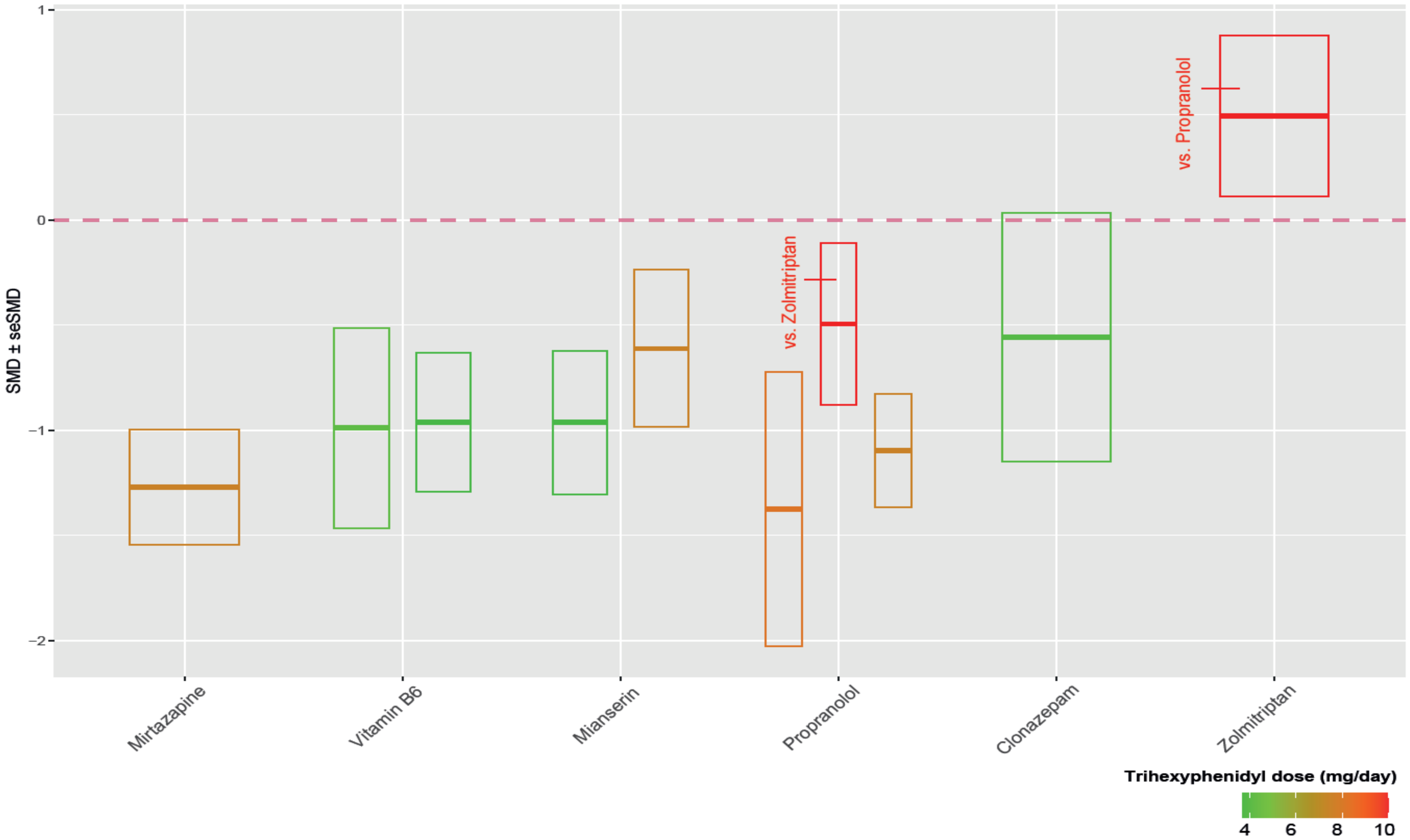

© 2024 Gerolymos C et al. *JAMA Network Open.*

eFigure 37. **Error bars showing the different standardized mean differences ± standard deviation for each intervention according to benztropine mean dose (mg/day).**

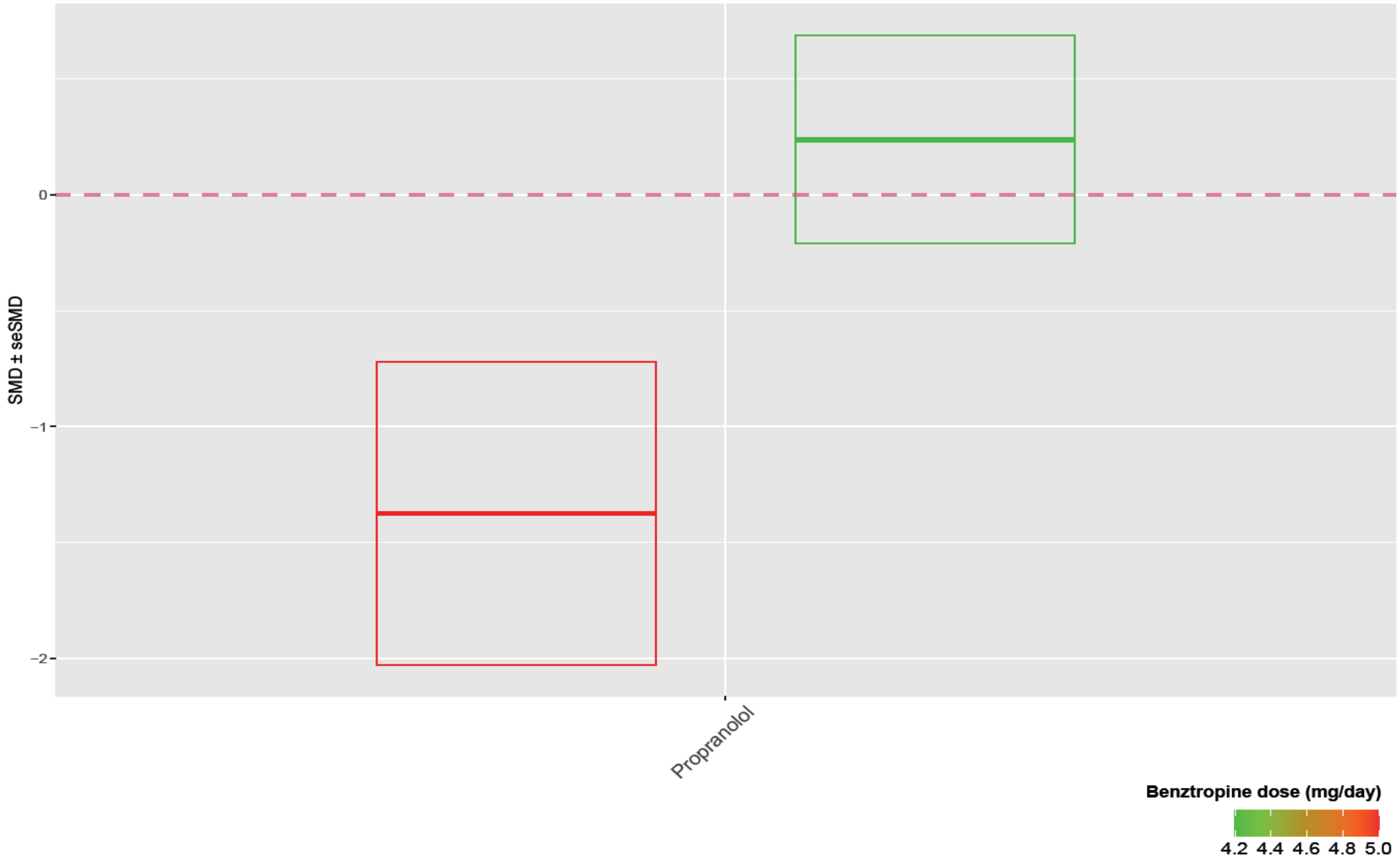

© 2024 Gerolymos C et al. *JAMA Network Open.*

eFigure 38. **Error bars showing the different standardized mean differences ± standard deviation for each intervention according to diazepam mean dose (mg/day), used as cotreatment drug.**

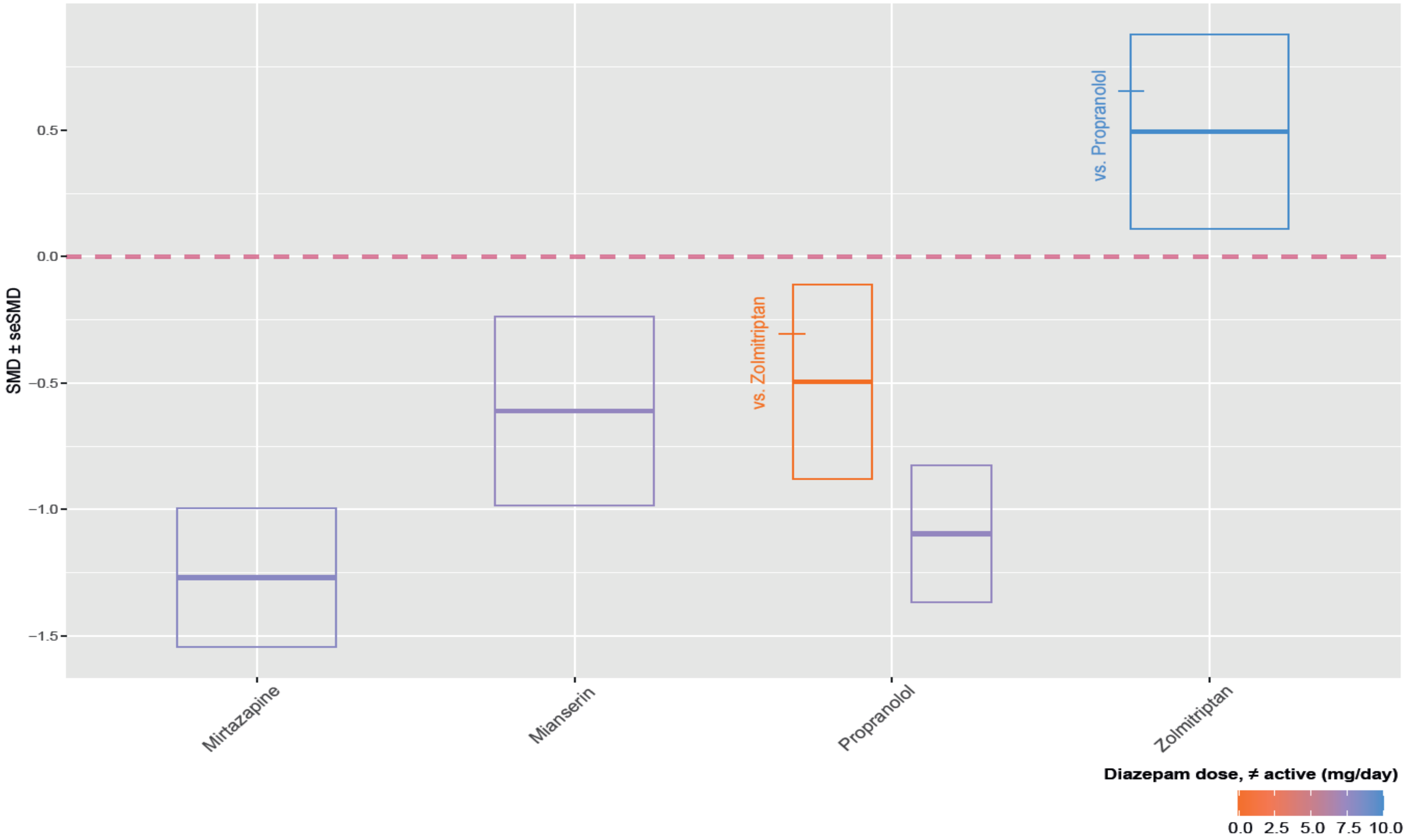

© 2024 Gerolymos C et al. *JAMA Network Open.*

eFigure 39. **Error bars showing the different standardized mean differences ± standard deviation for each intervention according to lorazepam mean dose (mg/day).**

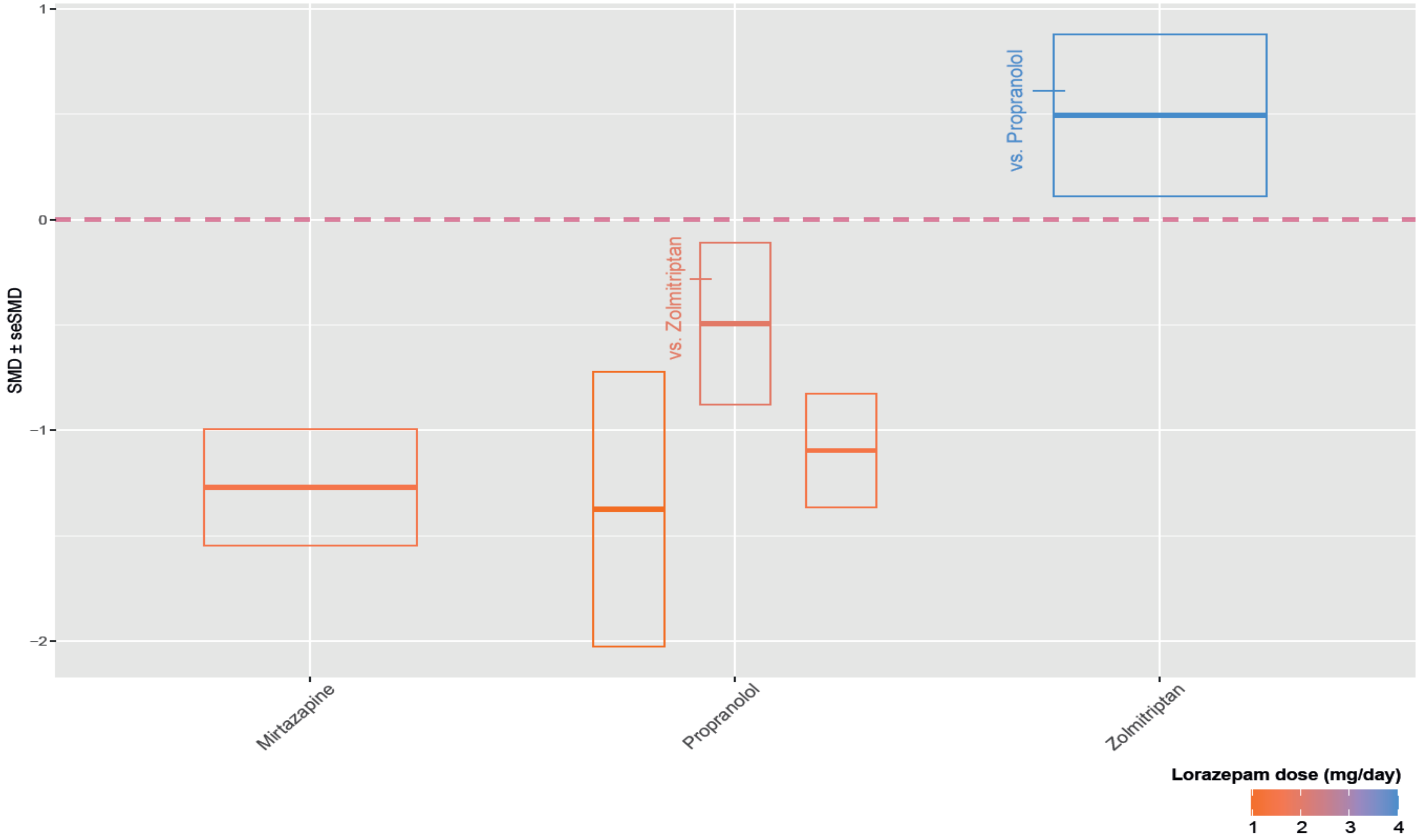

© 2024 Gerolymos C et al. *JAMA Network Open.*

eFigure 40. Error bars showing the different standardized mean differences ± standard deviation for each intervention according to nitrazepam mean dose (mg/day).

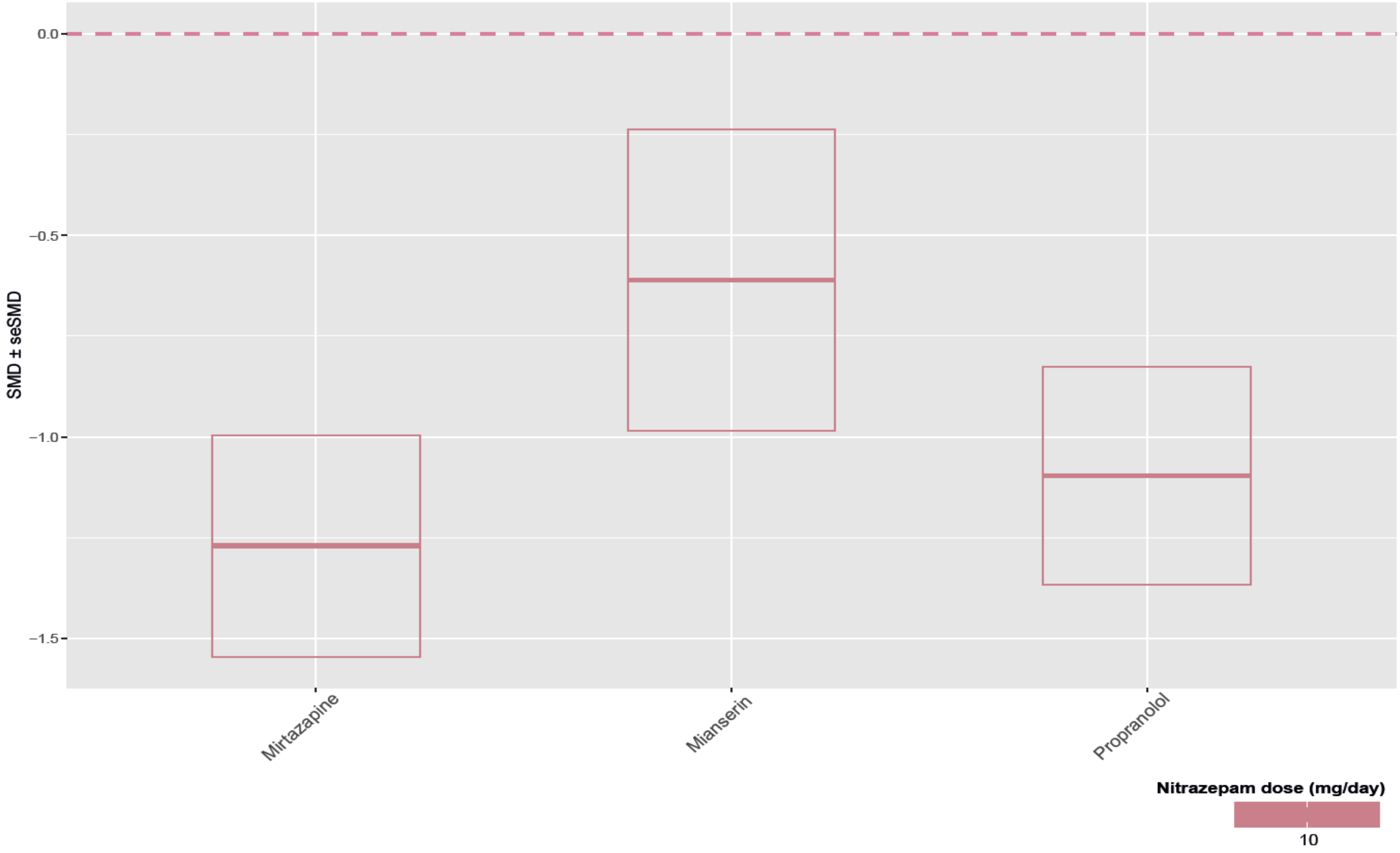

© 2024 Gerolymos C et al. *JAMA Network Open.*

eFigure 41. **Error bars showing the different standardized mean differences ± standard deviation for each intervention according to Simpson Angus Scale score (mean).**

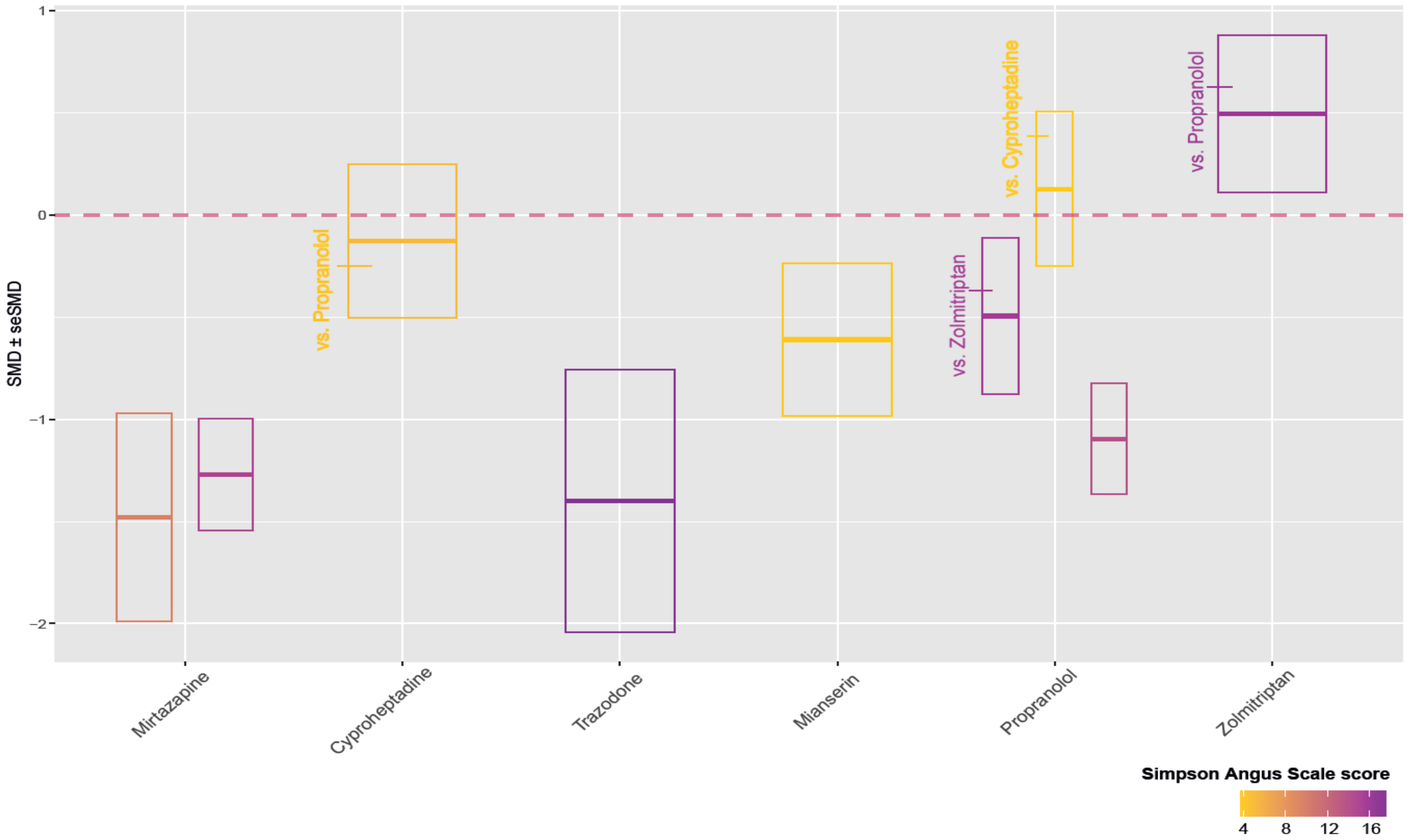

© 2024 Gerolymos C et al. *JAMA Network Open.*

eFigure 42. Error bars showing the different standardized mean differences ± standard deviation for each intervention according to Hamilton Depression Rating Scale (mean).

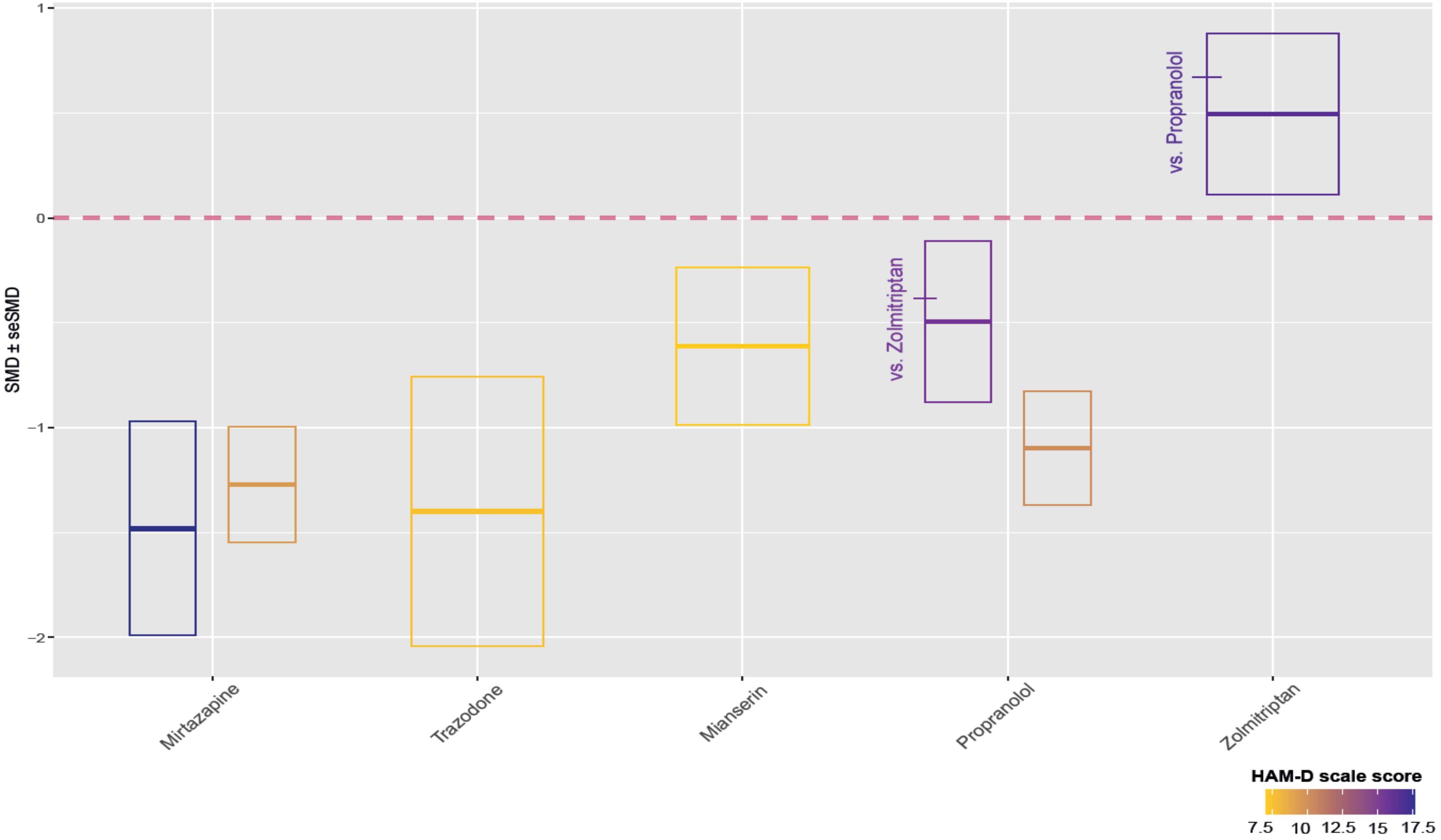

© 2024 Gerolymos C et al. *JAMA Network Open.*

3.2. Subgroup analyses

For the primary outcome, reduction of score in akathisia scale, we present below:

- Network graph
- Forest plot of the results of the network meta-analysis (reference: placebo)
- League table of the results for the network meta-analysis

Legend for network graphs: Node size is proportional to the total number of patients for each intervention. Line thickness is proportional to weight, from random effect model. The number overlying lines is equal to the number of studies corresponding to the comparison. Colored triangles represent comparisons for multi-arm studies.

Legend for forest plots: Effect sizes are from the random effect model of network-meta-analysis. The type of effect size measure (SMD, standardized mean differences) can be seen on top right.
Order of treatments is decreasing order of rank according to the surface under the cumulative ranking score (SUCRA). Reference is placebo.
Effect estimates to the left are in favor of experimental treatment. The more negative the value of SMD is, the higher is the reduction in akathisia scale.

Legend for league tables: Drugs are reported by decreasing rank order. Data are SMDs (95% CI). Data in bold are statistically significant.
Comparisons should be read from left to right.

For the lower triangle that reports network estimates, column-defining treatment is compared to row-defining treatment.

For the upper triangle that reports direct treatment estimates, row-defining treatment is compared to column-defining treatment.

SMDs above 0 favors the column-defining treatment in the lower triangle, whereas it favors the row-defining treatment in the upper triangle.
SMD, standardized mean difference; CI, confidence interval.

© 2024 Gerolymos C et al. *JAMA Network Open.*

In the first subgroup analysis, which only included studies with a low risk of bias, the network graph exhibited poor connectivity, resulting in only eight remaining studies (Friis et al. 1983; Kramer et al. 1988; Pujalte et al. 1994; Fischel et al. 2001; Poyurovsky et al. 2003; Lerner et al. 2004; Miodownik et al. 2006; Shams-Alizadeh et al. 2020). The results remained consistent with the main analysis for the top three ranked molecules. However, propranolol dropped to the last position, and its overall effect size was not statistically different from placebo (standardized mean difference [SMD] 0.24, 95% confidence interval [CI] [-0.64; 1.12]). Mianserin surpassed trazodone, securing the fourth position. According to network estimates, mirtazapine demonstrated statistically superior efficacy compared to cyproheptadine (SMD -1.48, 95% CI [-2.48; -0.48] and SMD 0.11, 95% CI [-1.04; 1.26], respectively). Biperiden also exhibited statistically superior efficacy compared to valproate and cyproheptadine (SMD -1.33, 95% CI [-2.09; -0.56], SMD -0.33, 95% CI [-1.05; -0.39] and SMD 0.11, 95% CI [-1.04; 1.26] respectively).

In the second subgroup analysis, restricted to trials with an AIA treatment duration of more than two days (Friis et al. 1983; Adler et al. 1986; Pujalte et al. 1994; Poyurovsky et al. 1999, 2003, 2006; Fischel et al. 2001; Lerner et al. 2004; Miodownik et al. 2006; Avital et al. 2009; Stryjer et al. 2010; Shams-Alizadeh et al. 2020), cyproheptadine claimed the third position and showed statistical superiority over placebo (SMD -1.29, 95% CI [-2.17; -0.41]). Additionally, the efficacy of propranolol improved compared to the main results, placing it fourth among the interventions (SMD -1.16, 95% CI [-1.64; -0.68]).

In the third subgroup analysis, which focused on studies utilizing the BARS scale for akathisia measurements (Pujalte et al. 1994; Poyurovsky et al. 1999, 2003, 2006; Fischel et al. 2001; Lerner et al. 2004; Miodownik et al. 2006; Baskak et al. 2007; Avital et al. 2009; Stryjer et al. 2010; Shams-Alizadeh et al. 2020), mirtazapine retained its position as the top-ranked molecule (SMD -1.32, 95% CI [-1.80; -0.85]). Cyproheptadine also exhibited statistical efficacy compared to placebo (SMD -1.25, 95% CI [-2.15; -0.35]), as did propranolol (SMD -1.13, 95% CI [-1.64; -0.61]). Individually, cyproheptadine and propranolol secured the second and third positions, respectively.

In the fourth and fifth subgroup analyses, including all trials and gathering SMDs by therapeutic classes, benzodiazepines (fourth and fifth analyses respectively: SMD -0.76, 95% CI [-1.72; 0.19] and SMD -0.74, 95% CI [-1.65; 0.17]) and antihistaminics (SMD -0.78, 95% CI [-1.66; 0.10] and SMD -0.73 95% CI [-1.56; 0.09]) didn't showed statistical superiority over placebo.

© 2024 Gerolymos C et al. *JAMA Network Open.*

eFigure 43. **Low-risk bias assessment, subgroup 1: network graph (random-effects model).**

Number of studies	Number of pairwise comparisons	Number of treatments	Number of designs
8	12	10	8

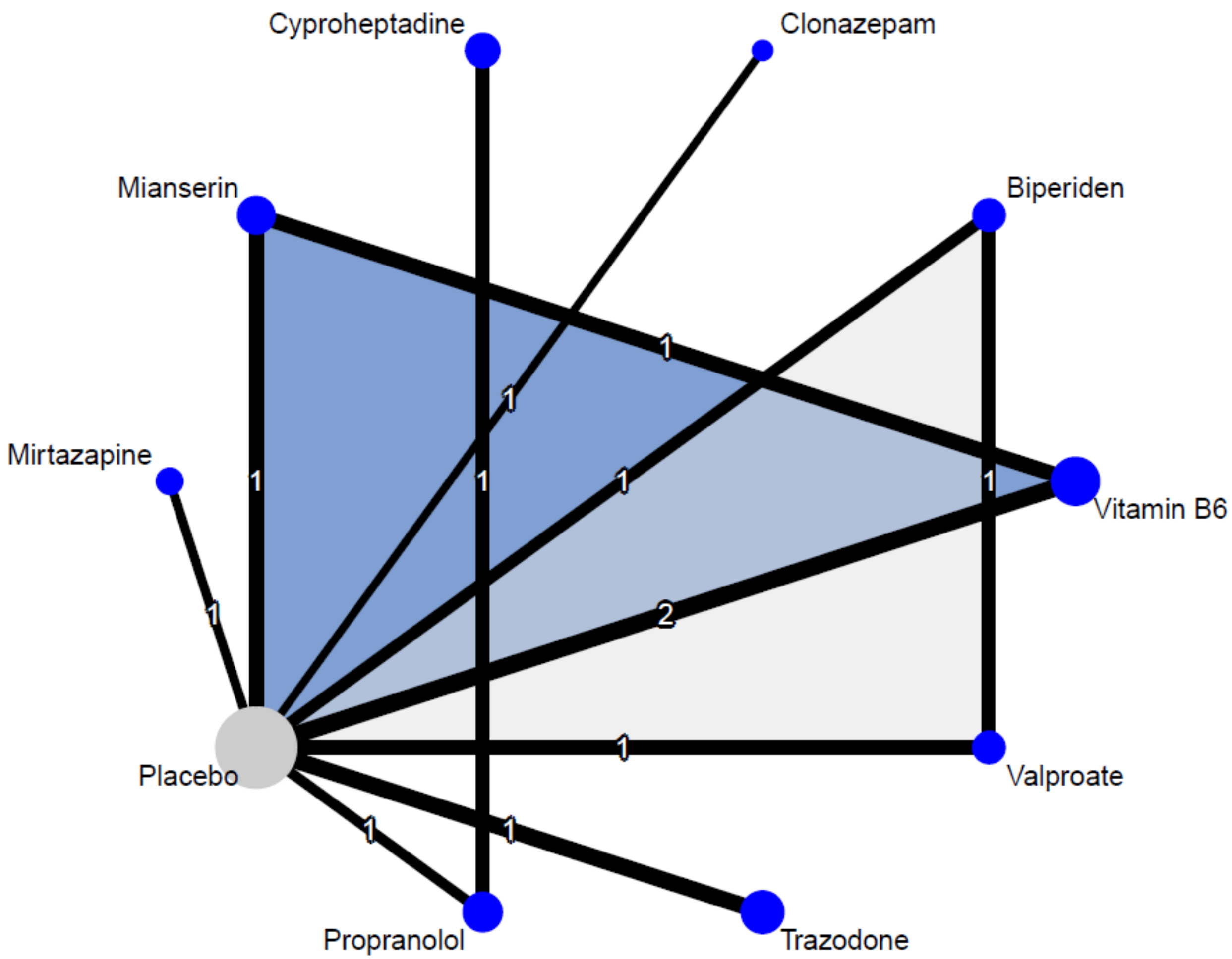

© 2024 Gerolymos C et al. *JAMA Network Open.*

eFigure 44. **Low-risk bias assessment, subgroup 1: forest plot (random-effects model).**

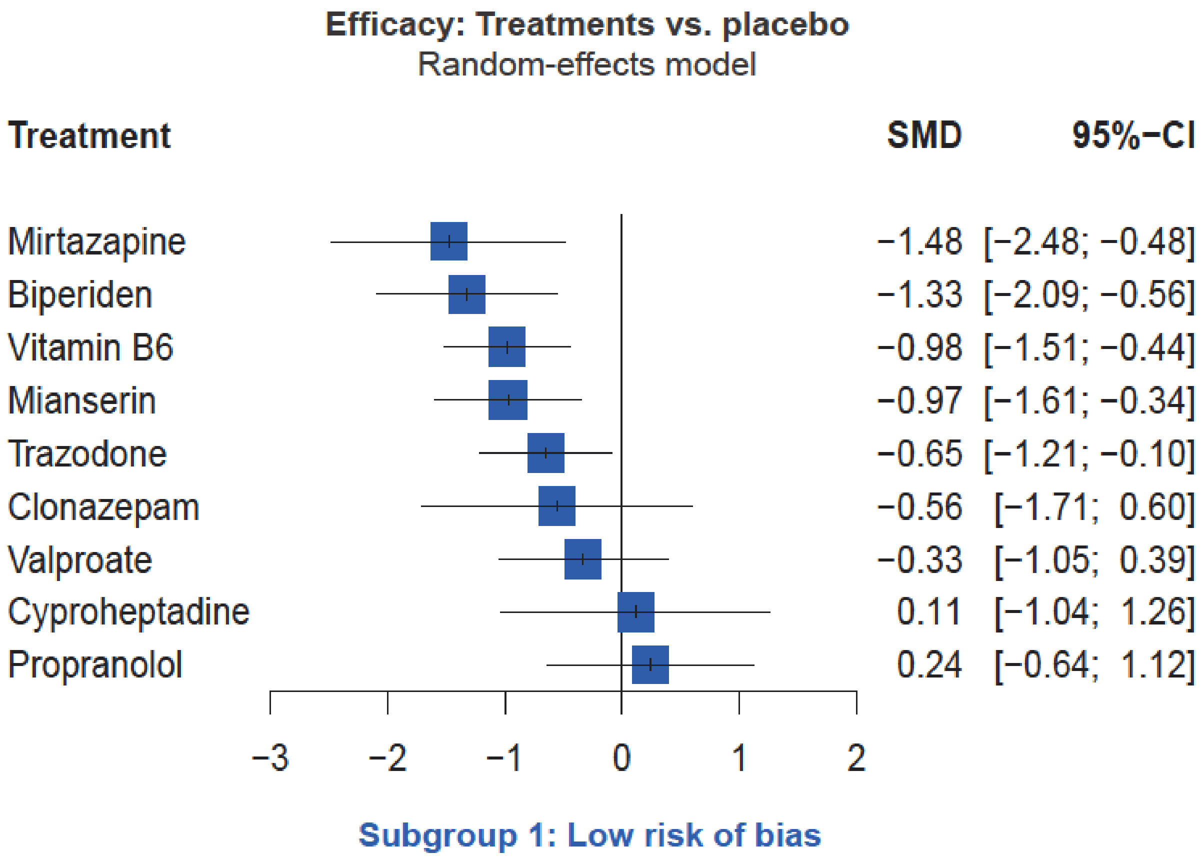

Heterogeneity (τ^2)	P-value for inconsistency (between designs)	Inconsistent comparisons of separable comparisons (SIDE-test p < 0.1)
0.0	0.97	0 / 2 (0%)

© 2024 Gerolymos C et al. *JAMA Network Open.*

eFigure 45. Low-risk bias assessment, subgroup 1: league table (random-effects model).

**Mirtazapine**	NA	NA	NA	NA	NA	NA	NA	**-1.48 [-2.48;-0.48]**	NA
-0.16 [-1.41; 1.10]	**Biperiden**	NA	NA	NA	NA	**-0.99 [-1.74;-0.25]**	NA	**-1.33 [-2.09;-0.56]**	NA
-0.50 [-1.64; 0.63]	-0.35 [-1.28; 0.59]	**Vitamin B6**	-0.00 [-0.60; 0.60]	NA	NA	NA	NA	**-0.98 [-1.51;-0.44]**	NA
-0.51 [-1.69; 0.68]	-0.35 [-1.34; 0.64]	-0.00 [-0.58; 0.58]	**Mianserin**	NA	NA	NA	NA	**-0.97 [-1.64;-0.30]**	NA
-0.83 [-1.97; 0.32]	-0.67 [-1.62; 0.28]	-0.32 [-1.10; 0.45]	-0.32 [-1.16; 0.52]	**Trazodone**	NA	NA	NA	**-0.65 [-1.21;-0.10]**	NA
-0.92 [-2.45; 0.61]	-0.77 [-2.16; 0.62]	-0.42 [-1.69; 0.86]	-0.42 [-1.73; 0.90]	-0.10 [-1.38; 1.19]	**Clonazepam**	NA	NA	-0.56 [-1.71; 0.60]	NA
-1.15 [-2.38; 0.08]	**-0.99 [-1.74;-0.25]**	-0.65 [-1.54; 0.25]	-0.64 [-1.60; 0.31]	-0.32 [-1.23; 0.59]	-0.23 [-1.59; 1.13]	**Valproate**	NA	-0.33 [-1.05; 0.39]	NA
**-1.59 [-3.12;-0.07]**	**-1.44 [-2.82;-0.06]**	-1.09 [-2.36; 0.18]	-1.09 [-2.40; 0.22]	-0.77 [-2.04; 0.51]	-0.67 [-2.30; 0.96]	-0.44 [-1.80; 0.91]	**Cyproheptadine**	NA	-0.13 [-0.87; 0.61]
**-1.48 [-2.48;-0.48]**	**-1.33 [-2.09;-0.56]**	**-0.98 [-1.51;-0.44]**	**-0.97 [-1.61;-0.34]**	**-0.65 [-1.21;-0.10]**	-0.56 [-1.71; 0.60]	-0.33 [-1.05; 0.39]	0.11 [-1.04; 1.26]	**Placebo**	-0.24 [-1.12; 0.64]
**-1.72 [-3.05;-0.39]**	**-1.56 [-2.73;-0.40]**	**-1.22 [-2.24;-0.19]**	**-1.21 [-2.30;-0.13]**	-0.89 [-1.93; 0.15]	-0.80 [-2.25; 0.66]	-0.57 [-1.71; 0.57]	-0.13 [-0.87; 0.61]	-0.24 [-1.12; 0.64]	**Propranolol**

© 2024 Gerolymos C et al. *JAMA Network Open.*

eFigure 46. Akathisia treatment duration > two days, subgroup 2: network graph (random-effects model).

Number of studies	Number of pairwise comparisons	Number of treatments	Number of designs
12	18	11	11

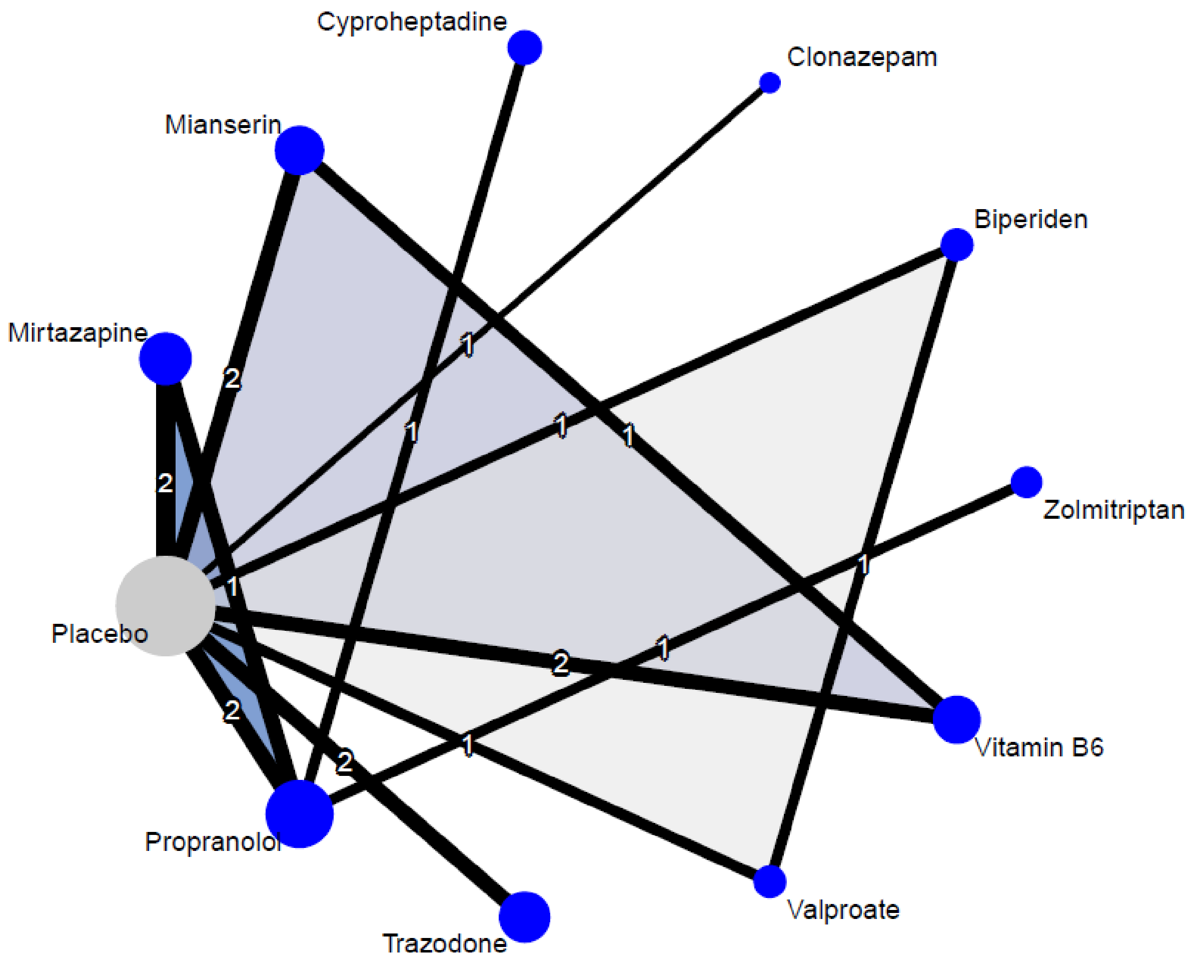

© 2024 Gerolymos C et al. *JAMA Network Open.*

eFigure 47. **Akathisia treatment duration > two days, subgroup 2: forest plot (random-effects model).**

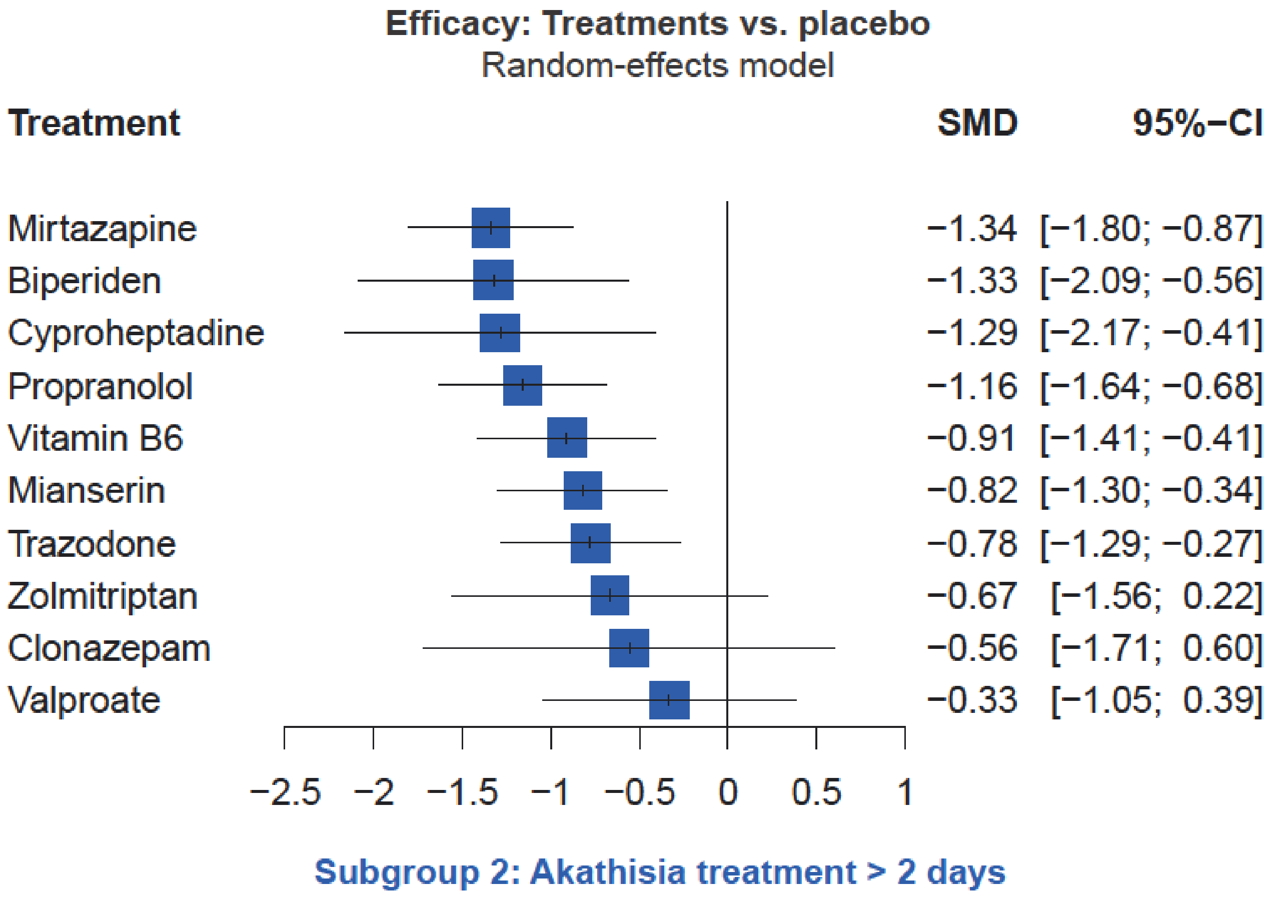

Heterogeneity (τ^2)	P-value for inconsistency (between designs)	Inconsistent comparisons of separable comparisons (SIDE-test p < 0.1)
0.0	0.94	0 / 6 (0%)

© 2024 Gerolymos C et al. *JAMA Network Open.*

eFigure 48. **Akathisia treatment duration > two days, subgroup 2: league table (random-effects model).**

**Mirtazapine**	NA	NA	-0.17<br>[-0.68; 0.33]	NA	NA	NA	NA	NA	NA	**-1.32<br>[-1.80;-0.85]**
-0.01<br>[-0.91; 0.88]	**Biperiden**	NA	NA	NA	NA	NA	NA	NA	**-0.99<br>[-1.74;-0.25]**	**-1.33<br>[-2.09;-0.56]**
-0.05<br>[-0.93; 0.83]	-0.04<br>[-1.20; 1.13]	**Cyproheptadine**	-0.13<br>[-0.87; 0.61]	NA	NA	NA	NA	NA	NA	NA
-0.18<br>[-0.66; 0.30]	-0.16<br>[-1.07; 0.74]	-0.13<br>[-0.87; 0.61]	**Propranolol**	NA	NA	NA	-0.49<br>[-1.25; 0.26]	NA	NA	**-1.14<br>[-1.63;-0.65]**
-0.43<br>[-1.11; 0.26]	-0.42<br>[-1.33; 0.50]	-0.38<br>[-1.39; 0.64]	-0.25<br>[-0.94; 0.44]	**Vitamin B6**	-0.00<br>[-0.60; 0.60]	NA	NA	NA	NA	**-0.98<br>[-1.51;-0.44]**
-0.52<br>[-1.19; 0.15]	-0.51<br>[-1.41; 0.40]	-0.47<br>[-1.47; 0.53]	-0.34<br>[-1.01; 0.33]	-0.09<br>[-0.62; 0.44]	**Mianserin**	NA	NA	NA	NA	**-0.81<br>[-1.30;-0.31]**
-0.56<br>[-1.25; 0.13]	-0.55<br>[-1.47; 0.37]	-0.51<br>[-1.53; 0.51]	-0.38<br>[-1.08; 0.31]	-0.13<br>[-0.85; 0.58]	-0.04<br>[-0.74; 0.66]	**Trazodone**	NA	NA	NA	**-0.78<br>[-1.29;-0.27]**
-0.67<br>[-1.57; 0.22]	-0.66<br>[-1.83; 0.52]	-0.62<br>[-1.68; 0.43]	-0.49<br>[-1.25; 0.26]	-0.24<br>[-1.27; 0.78]	-0.15<br>[-1.16; 0.86]	-0.11<br>[-1.14; 0.91]	**Zolmitriptan**	NA	NA	NA
-0.78<br>[-2.03; 0.47]	-0.77<br>[-2.16; 0.62]	-0.73<br>[-2.18; 0.72]	-0.60<br>[-1.85; 0.65]	-0.35<br>[-1.61; 0.91]	-0.26<br>[-1.51; 0.99]	-0.22<br>[-1.48; 1.05]	-0.11<br>[-1.57; 1.35]	**Clonazepam**	NA	-0.56<br>[-1.71; 0.60]
**-1.01<br>[-1.86;-0.15]**	**-0.99<br>[-1.74;-0.25]**	-0.96<br>[-2.09; 0.18]	-0.83<br>[-1.69; 0.03]	-0.58<br>[-1.46; 0.30]	-0.49<br>[-1.35; 0.37]	-0.45<br>[-1.33; 0.43]	-0.33<br>[-1.48; 0.81]	-0.23<br>[-1.59; 1.14]	**Valproate**	-0.33<br>[-1.05; 0.39]
**-1.34<br>[-1.80;-0.87]**	**-1.33<br>[-2.09;-0.56]**	**-1.29<br>[-2.17;-0.41]**	**-1.16<br>[-1.64;-0.68]**	**-0.91<br>[-1.41;-0.41]**	**-0.82<br>[-1.30;-0.34]**	**-0.78<br>[-1.29;-0.27]**	-0.67<br>[-1.56; 0.22]	-0.56<br>[-1.71; 0.60]	-0.33<br>[-1.05; 0.39]	**Placebo**

© 2024 Gerolymos C et al. *JAMA Network Open.*

eFigure 49. BARS as akathisia scale, subgroup 3: network graph (random-effects model).

Number of studies	Number of pairwise comparisons	Number of treatments	Number of designs
11	15	10	10

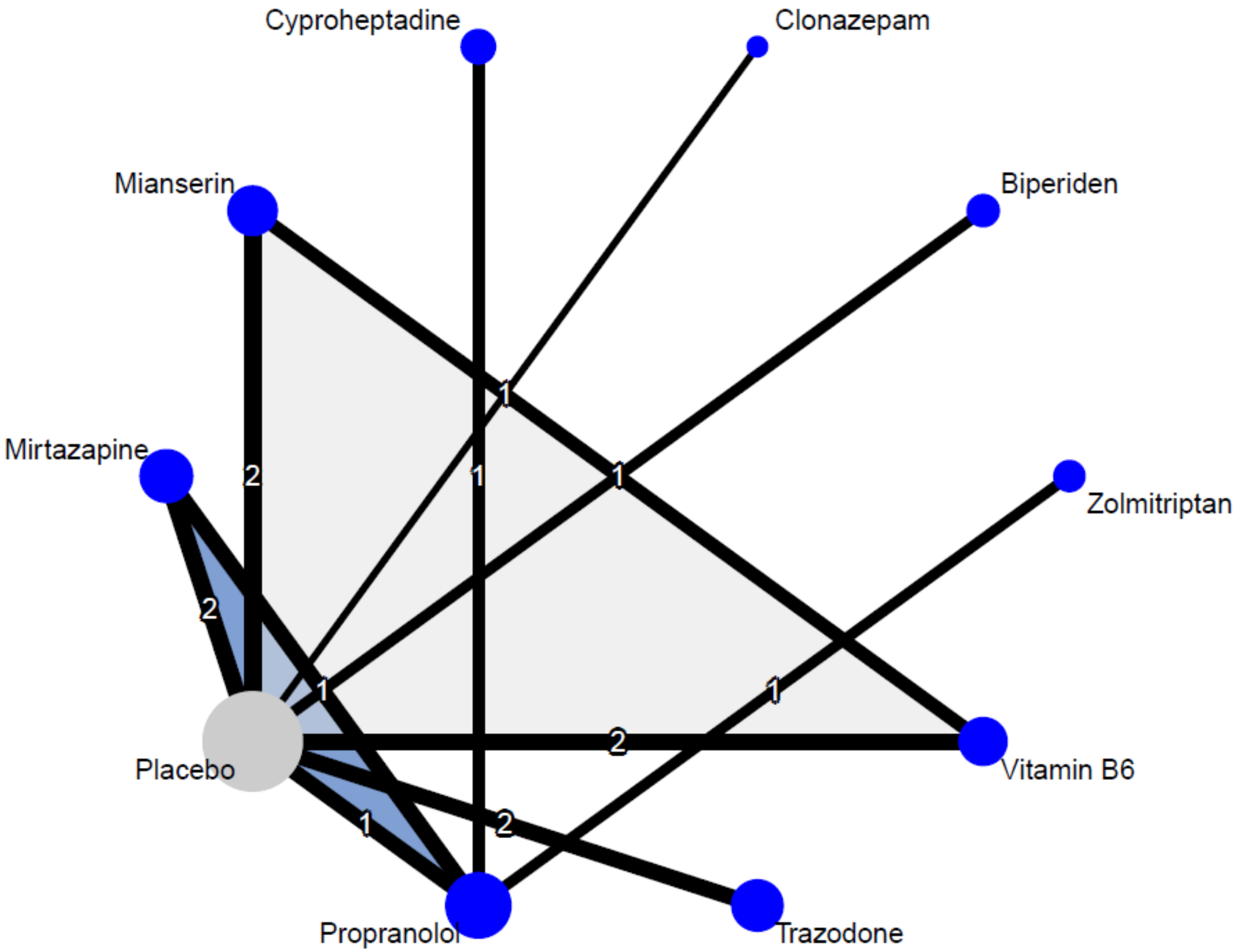

© 2024 Gerolymos C et al. *JAMA Network Open.*

eFigure 50. **BARS as akathisia scale, subgroup 3: forest plot (random-effects model).**

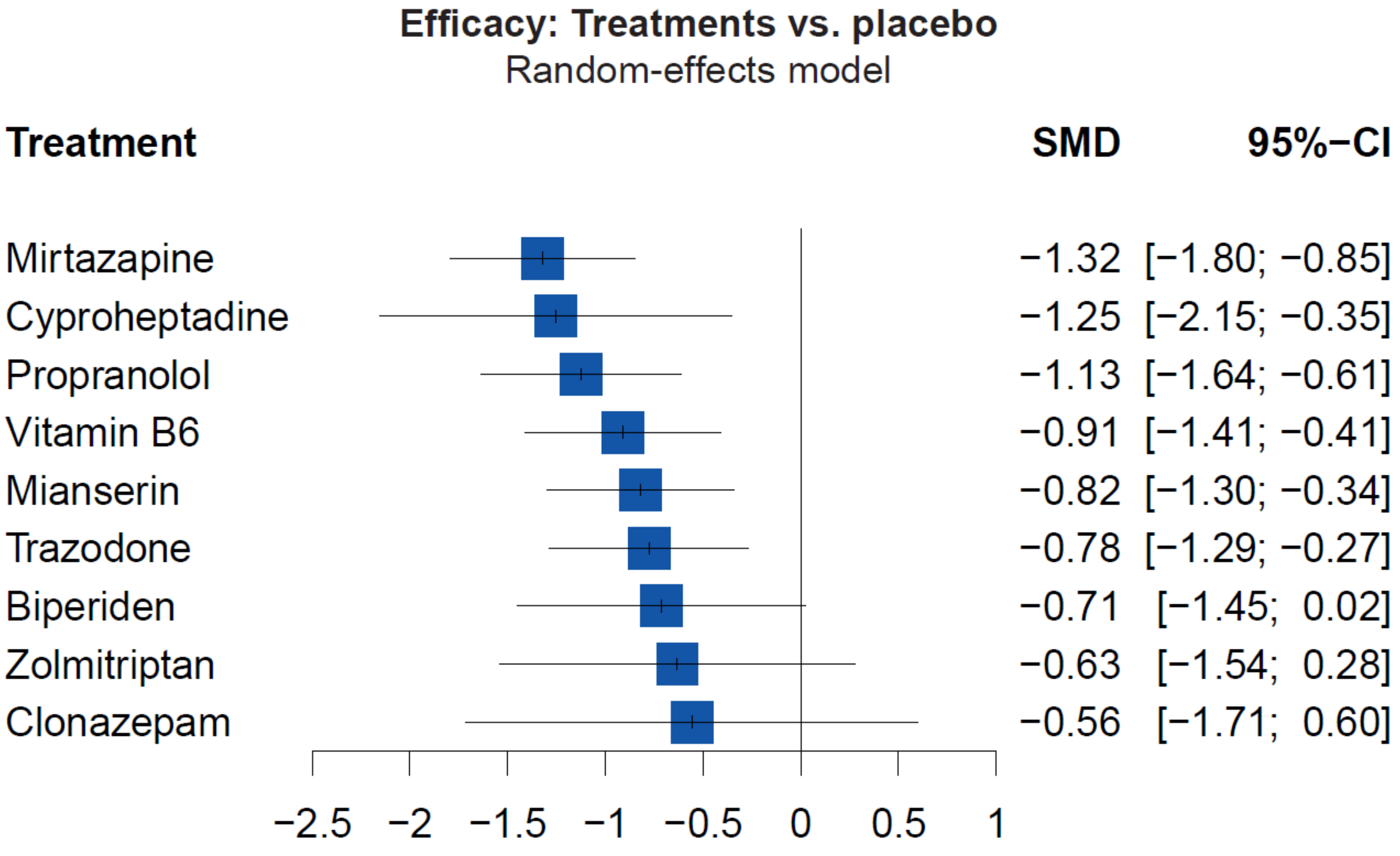

Subgroup 3: BARS scale

Heterogeneity (τ^2)	P-value for inconsistency (between designs)	Inconsistent comparisons of separable comparisons (SIDE-test p < 0.1)
0.0	0.88	0 / 5 (0%)

© 2024 Gerolymos C et al. *JAMA Network Open.*

eFigure 51. **BARS as akathisia scale, subgroup 3: league table (random-effects model).**

**Mirtazapine**	NA	-0.17 [-0.68; 0.33]	NA	NA	NA	NA	NA	NA	**-1.32 [-1.80;-0.85]**
-0.07 [-0.96; 0.82]	**Cyproheptadine**	-0.13 [-0.87; 0.61]	NA	NA	NA	NA	NA	NA	NA
-0.20 [-0.69; 0.30]	-0.13 [-0.87; 0.61]	**Propranolol**	NA	NA	NA	NA	-0.49 [-1.25; 0.26]	NA	**-1.10 [-1.63;-0.57]**
-0.41 [-1.10; 0.28]	-0.34 [-1.37; 0.69]	-0.22 [-0.93; 0.50]	**Vitamin B6**	-0.00 [-0.60; 0.60]	NA	NA	NA	NA	**-0.98 [-1.51;-0.44]**
-0.50 [-1.18; 0.17]	-0.43 [-1.45; 0.59]	-0.31 [-1.01; 0.39]	-0.09 [-0.62; 0.44]	**Mianserin**	NA	NA	NA	NA	**-0.81 [-1.30;-0.31]**
-0.55 [-1.24; 0.15]	-0.48 [-1.51; 0.56]	-0.35 [-1.07; 0.37]	-0.13 [-0.85; 0.59]	-0.04 [-0.74; 0.66]	**Trazodone**	NA	NA	NA	**-0.78 [-1.29;-0.27]**
-0.61 [-1.48; 0.27]	-0.54 [-1.70; 0.63]	-0.41 [-1.31; 0.49]	-0.19 [-1.09; 0.70]	-0.10 [-0.98; 0.77]	-0.06 [-0.96; 0.84]	**Biperiden**	NA	NA	-0.71 [-1.45; 0.02]
-0.69 [-1.59; 0.21]	-0.62 [-1.68; 0.43]	-0.49 [-1.25; 0.26]	-0.28 [-1.32; 0.76]	-0.19 [-1.22; 0.84]	-0.14 [-1.19; 0.90]	-0.08 [-1.25; 1.09]	**Zolmitriptan**	NA	NA
-0.76 [-2.01; 0.49]	-0.70 [-2.16; 0.77]	-0.57 [-1.83; 0.70]	-0.35 [-1.61; 0.91]	-0.26 [-1.51; 0.99]	-0.22 [-1.48; 1.05]	-0.16 [-1.53; 1.22]	-0.07 [-1.55; 1.40]	**Clonazepam**	-0.56 [-1.71; 0.60]
**-1.32 [-1.80;-0.85]**	**-1.25 [-2.15;-0.35]**	**-1.13 [-1.64;-0.61]**	**-0.91 [-1.41;-0.41]**	**-0.82 [-1.30;-0.34]**	**-0.78 [-1.29;-0.27]**	-0.71 [-1.45; 0.02]	-0.63 [-1.54; 0.28]	-0.56 [-1.71; 0.60]	**Placebo**

© 2024 Gerolymos C et al. *JAMA Network Open.*

eFigure 52. Benzodiazepines, antihistaminics and other actives vs. placebo, subgroup 4: network graph (random-effects model).

Number of studies	Number of pairwise comparisons	Number of treatments	Number of designs
15	21	11	13

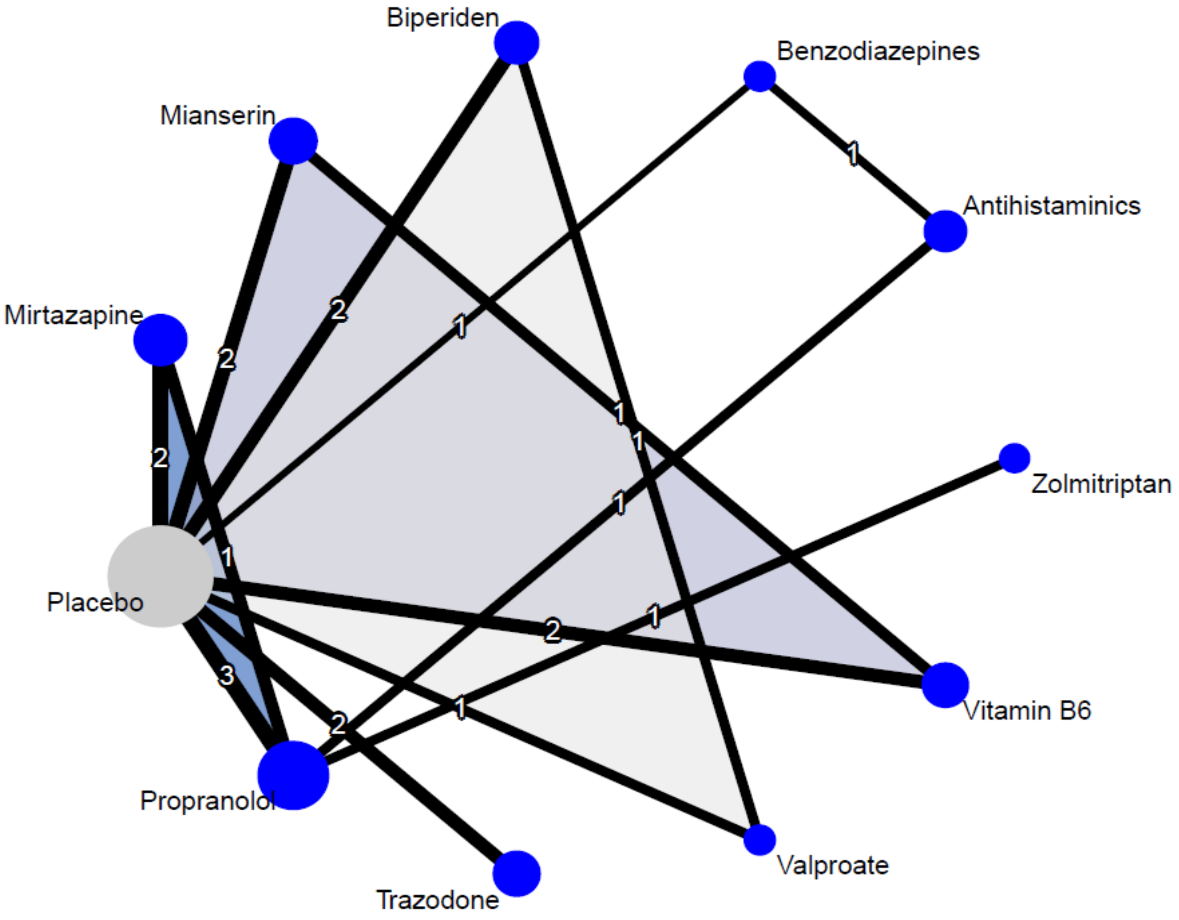

Description of treatment groups. The following groups gather the standardized mean differences from the drugs listed below:

- "**Benzodiazepines**": diazepam, diphenhydramine.
- "**Antihistaminics**" (first-generation): diphenhydramine, cyproheptadine.

© 2024 Gerolymos C et al. *JAMA Network Open.*

eFigure 53. **Benzodiazepines, antihistaminics and other actives vs. placebo, subgroup 4: forest plot (random-effects model).**

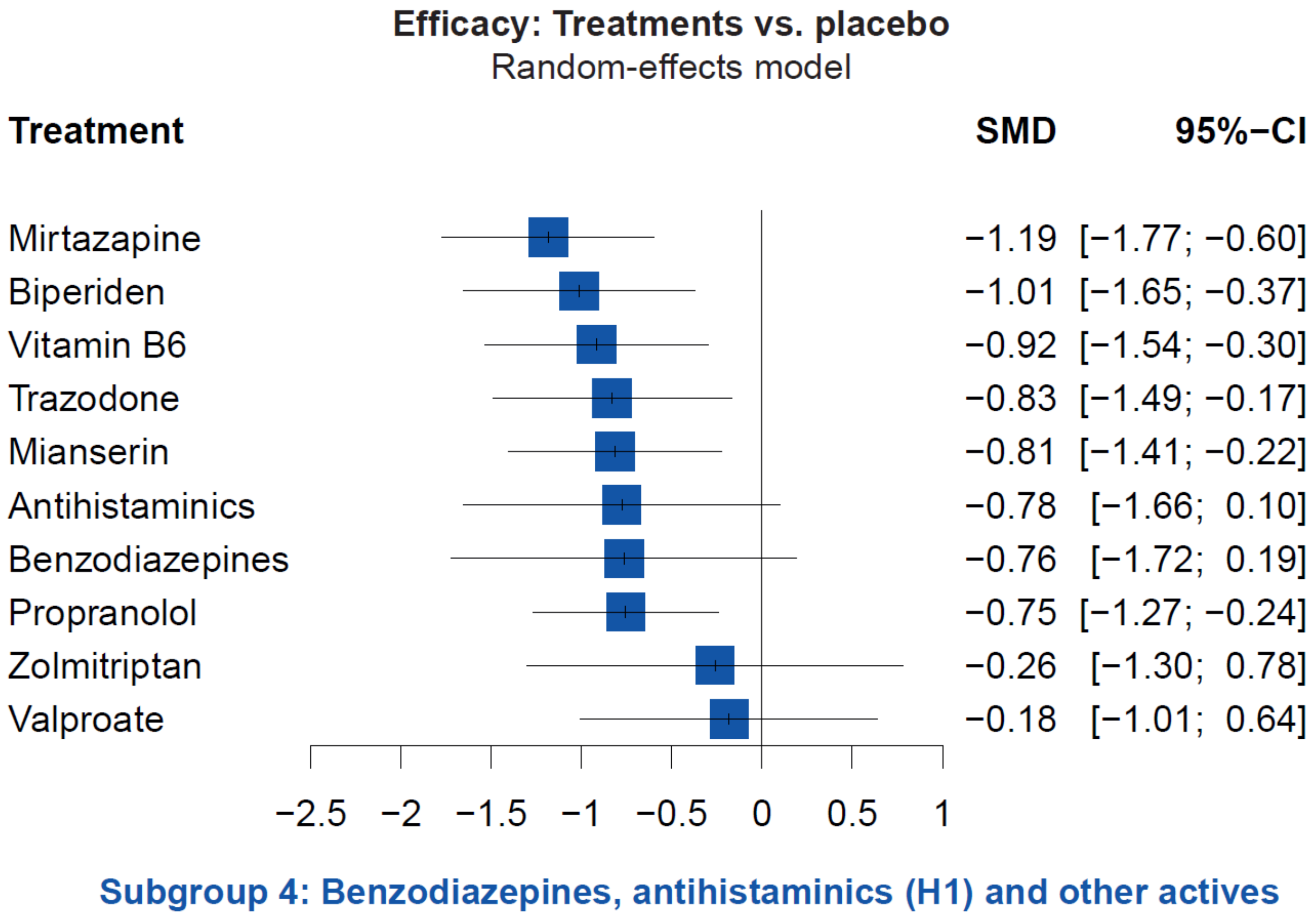

Heterogeneity (τ^2)	P-value for inconsistency (between designs)	Inconsistent comparisons of separable comparisons (SIDE-test p < 0.1)
0.0679	0.44	0 / 11 (0%)

© 2024 Gerolymos C et al. *JAMA Network Open.*

eFigure 54. Benzodiazepines, antihistaminics and other actives vs. placebo, subgroup 4: league table (random-effects model).

**Mirtazapine**	NA	NA	NA	NA	NA	NA	-0.17 [-0.89; 0.54]	NA	NA	**-1.34 [-1.96;-0.72]**
-0.17 [-1.04; 0.70]	**Biperiden**	NA	NA	NA	NA	NA	NA	NA	**-0.99 [-1.90;-0.09]**	**-1.01 [-1.65;-0.37]**
-0.27 [-1.12; 0.59]	-0.10 [-0.99; 0.80]	**Vitamin B6**	NA	-0.00 [-0.79; 0.79]	NA	NA	NA	NA	NA	**-0.98 [-1.63;-0.32]**
-0.35 [-1.24; 0.53]	-0.18 [-1.10; 0.74]	-0.09 [-0.99; 0.82]	**Trazodone**	NA	NA	NA	NA	NA	NA	**-0.83 [-1.49;-0.17]**
-0.37 [-1.20; 0.46]	-0.20 [-1.07; 0.68]	-0.10 [-0.78; 0.58]	-0.02 [-0.90; 0.87]	**Mianserin**	NA	NA	NA	NA	NA	**-0.80 [-1.41;-0.19]**
-0.41 [-1.39; 0.57]	-0.23 [-1.32; 0.85]	-0.14 [-1.22; 0.94]	-0.05 [-1.15; 1.05]	-0.04 [-1.10; 1.02]	**Antihistaminics**	0.12 [-0.90; 1.14]	-0.13 [-1.03; 0.77]	NA	NA	NA
-0.42 [-1.50; 0.66]	-0.25 [-1.40; 0.90]	-0.15 [-1.29; 0.99]	-0.07 [-1.23; 1.10]	-0.05 [-1.17; 1.07]	-0.01 [-0.88; 0.85]	**Benzodiazepines**	NA	NA	NA	-0.56 [-1.82; 0.71]
-0.43 [-1.06; 0.20]	-0.26 [-1.08; 0.57]	-0.16 [-0.97; 0.64]	-0.08 [-0.91; 0.76]	-0.06 [-0.84; 0.72]	-0.02 [-0.82; 0.77]	-0.01 [-0.98; 0.96]	**Propranolol**	-0.49 [-1.40; 0.41]	NA	-0.76 [-1.30;-0.21]
-0.92 [-2.03; 0.18]	-0.75 [-1.98; 0.47]	-0.66 [-1.87; 0.56]	-0.57 [-1.81; 0.67]	-0.55 [-1.75; 0.65]	-0.52 [-1.72; 0.69]	-0.50 [-1.83; 0.82]	-0.49 [-1.40; 0.41]	**Zolmitriptan**	NA	NA
-1.00 [-2.02; 0.01]	-0.83 [-1.67; 0.00]	-0.73 [-1.77; 0.30]	-0.65 [-1.70; 0.41]	-0.63 [-1.65; 0.38]	-0.60 [-1.80; 0.61]	-0.58 [-1.84; 0.68]	-0.57 [-1.54; 0.40]	-0.08 [-1.41; 1.25]	**Valproate**	-0.33 [-1.21; 0.55]
**-1.19 [-1.77;-0.60]**	**-1.01 [-1.65;-0.37]**	**-0.92 [-1.54;-0.30]**	**-0.83 [-1.49;-0.17]**	**-0.81 [-1.41;-0.22]**	-0.78 [-1.66; 0.10]	-0.76 [-1.72; 0.19]	**-0.75 [-1.27;-0.24]**	-0.26 [-1.30; 0.78]	-0.18 [-1.01; 0.64]	**Placebo**

© 2024 Gerolymos C et al. *JAMA Network Open.*

eFigure 55. **Therapeutic classes in akathisia vs. placebo, subgroup 5: network graph (random-effects model).**

Number of studies	Number of pairwise comparisons	Number of treatments	Number of designs
15	21	9	11

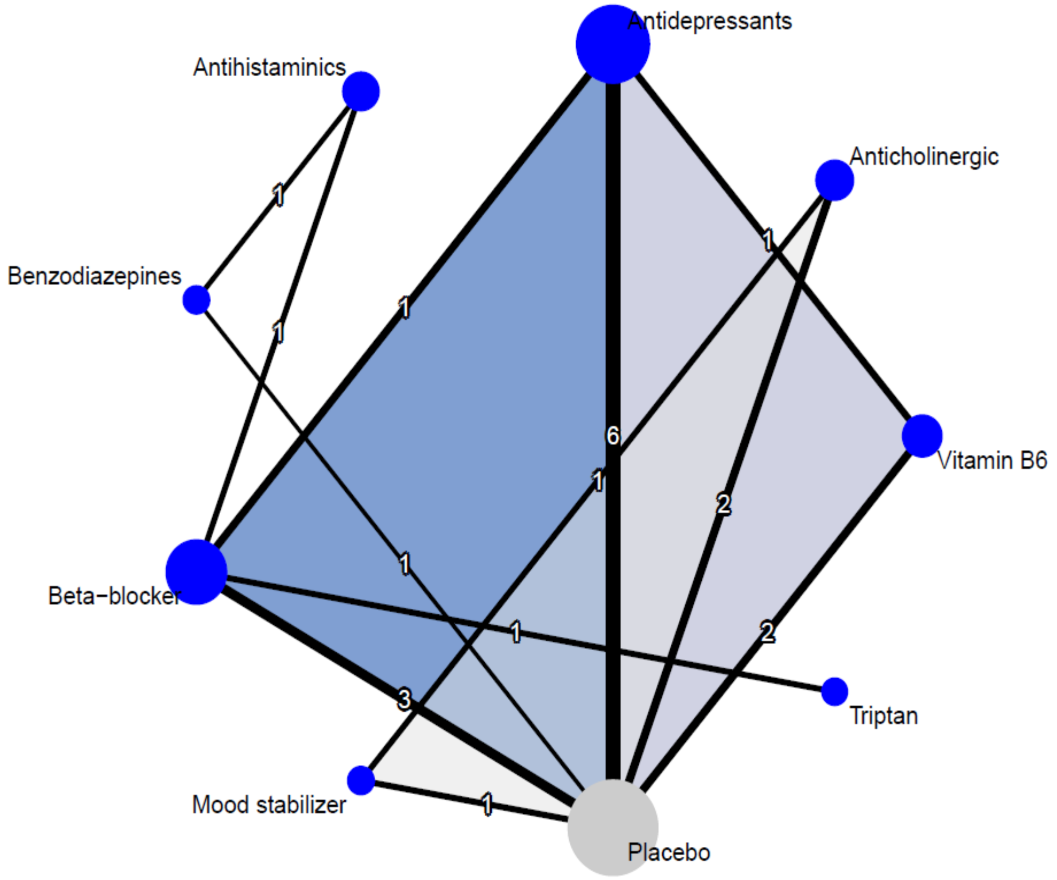

Description of treatment groups. The following groups gather the standardized mean differences from the drugs listed below:

- "**Benzodiazepines**": diazepam, diphenhydramine.
- "**Antihistaminics**" (first-generation): diphenhydramine, cyproheptadine.
- "**Antidepressants**": mirtazapine, mianserin, trazodone.
- "**Beta-blocker**": propranolol.
- "**Triptan**": zolmitriptan.
- "**Mood stabilizer**": valproate.

© 2024 Gerolymos C et al. *JAMA Network Open.*

eFigure 56. **Therapeutic classes in akathisia vs. placebo, subgroup 5: forest plot (random-effects model).**

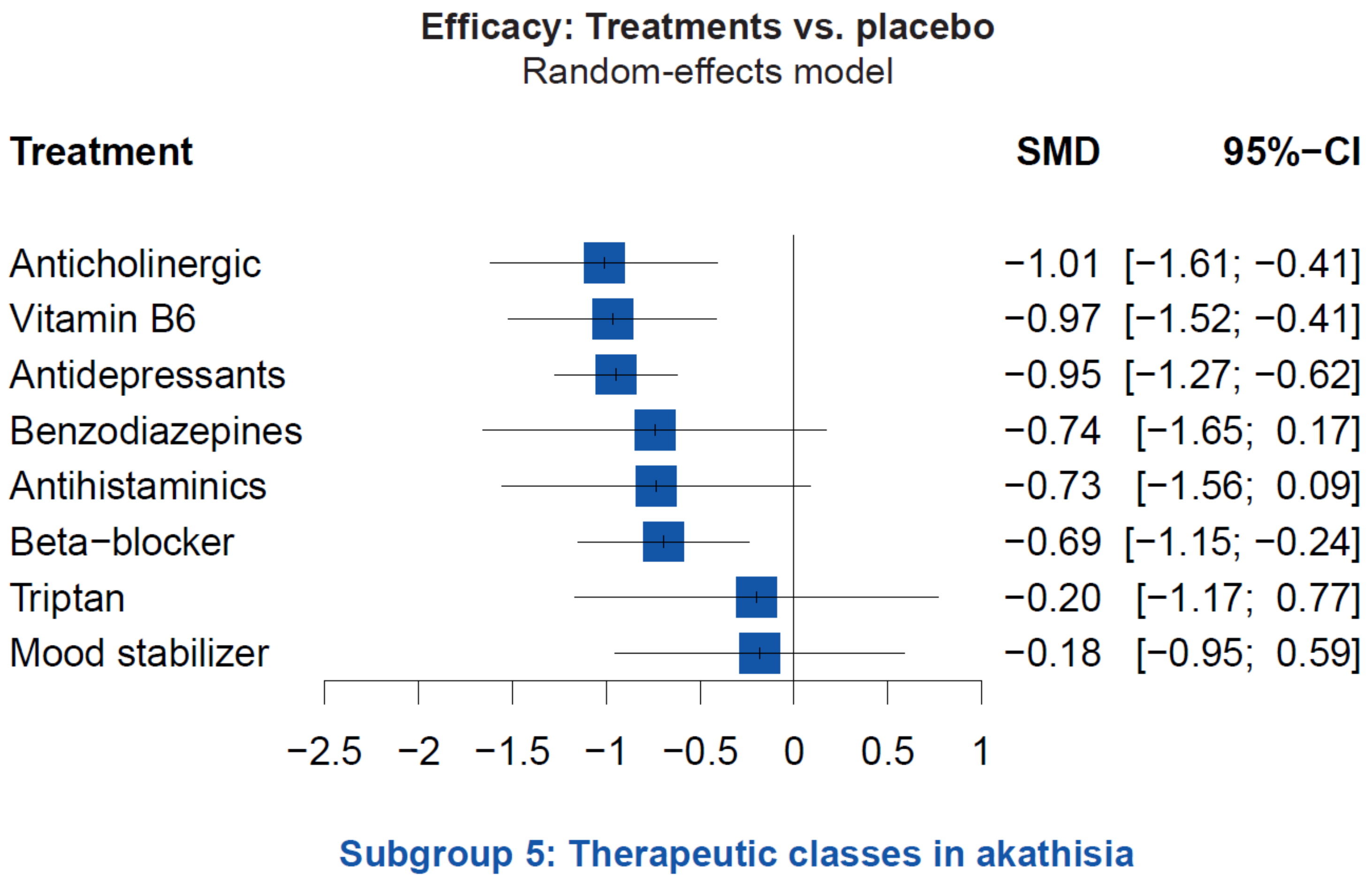

Heterogeneity (τ^2)	P-value for inconsistency (between designs)	Inconsistent comparisons of separable comparisons (SIDE-test p < 0.1)
0.0425	0.48	0 / 10 (0%)

© 2024 Gerolymos C et al. *JAMA Network Open.*

eFigure 57. Therapeutic classes in akathisia vs. placebo, subgroup 5: league table (random-effects model).

**Anticholinergic**	NA	NA	NA	NA	NA	NA	**-0.99<br>[-1.84;-0.15]**	**-1.00<br>[-1.61;-0.41]**
-0.06<br>[-0.75; 0.62]	**Antidepressants**	0.00<br>[-0.72; 0.72]	NA	NA	**-0.17<br>[-0.82; 0.47]**	NA	NA	**-0.99<br>[-1.33;-0.65]**
-0.04<br>[-0.86; 0.77]	0.02<br>[-0.55; 0.59]	**Vitamin B6**	NA	NA	NA	NA	NA	**-0.98<br>[-1.59;-0.37]**
-0.27<br>[-1.37; 0.82]	-0.21<br>[-1.16; 0.75]	-0.23<br>[-1.29; 0.83]	**Benzodiazepines**	-0.12<br>[-1.09; 0.85]	NA	NA	NA	**-0.56<br>[-1.78; 0.67]**
-0.28<br>[-1.30; 0.74]	-0.21<br>[-1.07; 0.64]	-0.23<br>[-1.21; 0.75]	-0.01<br>[-0.83; 0.82]	**Antihistaminics**	-0.13<br>[-0.97; 0.72]	NA	NA	NA
-0.32<br>[-1.07; 0.44]	-0.25<br>[-0.75; 0.24]	-0.27<br>[-0.97; 0.42]	-0.05<br>[-0.97; 0.87]	-0.04<br>[-0.79; 0.71]	**Beta-blocker**	-0.49<br>[-1.35; 0.36]	NA	**-0.77<br>[-1.28;-0.26]**
-0.81<br>[-1.95; 0.33]	-0.75<br>[-1.74; 0.24]	-0.77<br>[-1.87; 0.33]	-0.54<br>[-1.80; 0.71]	-0.53<br>[-1.67; 0.60]	-0.49<br>[-1.35; 0.36]	**Triptan**	NA	NA
**-0.83<br>[-1.61;-0.05]**	-0.77<br>[-1.60; 0.07]	-0.78<br>[-1.73; 0.16]	-0.56<br>[-1.75; 0.64]	-0.55<br>[-1.68; 0.57]	-0.51<br>[-1.41; 0.38]	-0.02<br>[-1.25; 1.22]	**Mood stabilizer**	-0.33<br>[-1.16; 0.49]
**-1.01<br>[-1.61;-0.41]**	**-0.95<br>[-1.27;-0.62]**	**-0.97<br>[-1.52;-0.41]**	-0.74<br>[-1.65; 0.17]	-0.73<br>[-1.56; 0.09]	**-0.69<br>[-1.15;-0.24]**	-0.20<br>[-1.17; 0.77]	-0.18<br>[-0.95; 0.59]	**Placebo**

© 2024 Gerolymos C et al. *JAMA Network Open.*

5. eReferences

Supplementary text 3. References of Supplementary materials.

Adler L, Angrist B, Peselow E, Corwin J, Maslansky R, Rotrosen J. A Controlled Assessment of Propranolol in the Treatment of Neuroleptic-Induced Akathisia. Br J Psychiatry. juill 1986;149(1):42-5.

Avital A, Gross-Isseroff R, Stryjer R, Hermesh H, Weizman A, Shiloh R. Zolmitriptan compared to propranolol in the treatment of acute neuroleptic-induced akathisia: A comparative double-blind study. European Neuropsychopharmacology. juill 2009;19(7):476-82.

Balduzzi S, Rücker G, Schwarzer G. How to perform a meta-analysis with R: a practical tutorial. Evid Based Mental Health. nov 2019;22(4):153-60.

Barnes TRE. The Barnes Akathisia Rating Scale–Revisited. J Psychopharmacol. déc 2003;17(4):365-70.

Baskak B, Atbasoglu EC, Ozguven HD, Saka MC, Gogus AK. The Effectiveness of Intramuscular Biperiden in Acute Akathisia: A Double-Blind, Randomized, Placebo-Controlled Study. Journal of Clinical Psychopharmacology. juin 2007;27(3):289-94.

Braude WM, Barnes TR, Gore SM. Clinical characteristics of akathisia. A systematic investigation of acute psychiatric inpatient admissions. Br J Psychiatry. août 1983;143:139-50.

Crippa A, Orsini N. Dose-response meta-analysis of differences in means. BMC Med Res Methodol. déc 2016;16(1):91.

Egger M, Smith GD, Schneider M, Minder C. Bias in meta-analysis detected by a simple, graphical test. BMJ. 13 sept 1997;315(7109):629-34.

Fischel T, Hermesh H, Aizenberg D, Zemishlany Z, Munitz H, Benjamini Y, et al. Cyproheptadine Versus Propranolol for the Treatment of Acute Neuroleptic-Induced Akathisia: A Comparative Double-Blind Study: Journal of Clinical Psychopharmacology. déc 2001;21(6):612-5.

Friis T, Christensen TR, Gerlach J. Sodium vaiproate and biperiden in neuroleptic-induced akathisia, parkinsonism and hyperkinesia.: A double-blind cross-over study with placebo. Acta Psychiatr Scand. mars 1983;67(3):178-87.

Gagrat D, Hamilton J, Belmaker RH. Intravenous diazepam in the treatment of neuroleptic-induced acute dystonia and akathisia. Am J Psychiatry. oct 1978;135(10):1232-3.

Gardner DM, Murphy AL, O'Donnell H, Pharm B, Centorrino F, Baldessarini RJ. International Consensus Study of Antipsychotic Dosing. Am J Psychiatry. 2010;

Hedges LV. Distribution Theory for Glass's Estimator of Effect size and Related Estimators. Journal of Educational Statistics. juin 1981;6(2):107-28.

Higgins JPT. Measuring inconsistency in meta-analyses. BMJ. 6 sept 2003;327(7414):557-60.

Higgins JPT. Commentary: Heterogeneity in meta-analysis should be expected and appropriately quantified. International Journal of Epidemiology. 1 oct 2008;37(5):1158-60.

Janno S, Holi MM, Tuisku K, Wahlbeck K. Validity of Simpson-Angus Scale (SAS) in a naturalistic schizophrenia population. BMC Neurol. déc 2005;5(1):5.

Kramer MS, Gorkin RA, DiJohnson C, Sheves P. Propranolol in the treatment of neuroleptic-induced akathisia (NIA) in schizophrenics: A double-blind, placebo-controlled study. Biological Psychiatry. nov 1988;24(7):823-7.

Lerner V, Bergman J, Statsenko N, Miodownik C. Vitamin B6 Treatment in Acute Neuroleptic-Induced Akathisia: A Randomized, Double-Blind, Placebo-Controlled Study. J Clin Psychiatry. 15 nov 2004;65(11):1550-4.

Leucht S, Rothe P, Davis JM, Engel RR. Equipercentile linking of the BPRS and the PANSS. European Neuropsychopharmacology. août 2013;23(8):956-9.

Miodownik C, Lerner V, Statsenko N, Dwolatzky T, Nemets B, Berzak E, et al. Vitamin B6 Versus Mianserin and Placebo in Acute Neuroleptic-induced Akathisia: A Randomized, Double-blind, Controlled Study. Clin Neuropharmacol. 2006;29(2).

Poyurovsky M, Epshtein S, Fuchs C, Schneidman M, Weizman R, Weizman A. Efficacy of Low-Dose Mirtazapine in Neuroleptic-Induced Akathisia: A Double-Blind Randomized Placebo-Controlled Pilot Study: Journal of Clinical Psychopharmacology. juin 2003;23(3):305-8.

© 2024 Gerolymos C et al. *JAMA Network Open.*

Poyurovsky M, Pashinian A, Weizman R, Fuchs C, Weizman A. Low-Dose Mirtazapine: A New Option in the Treatment of Antipsychotic-Induced Akathisia. A Randomized, Double-Blind, Placebo- and Propranolol-Controlled Trial. Biological Psychiatry. juin 2006;59(11):1071-7.

Poyurovsky M, Shardorodsky M, Fuchs C, Schneidman M, Weizman A. Treatment of neuroleptic-induced akathisia with the 5-HT $_2$ antagonist mianserin: Double-blind, placebo-controlled study. Br J Psychiatry. mars 1999;174(3):238-42.

Pujalte D, Bottaï T, Huë B, Alric R, Pouget R, Blayac JP, et al. A double-blind comparison of clonazepam and placebo in the treatment of neuroleptic-induced akathisia. Clin Neuropharmacol. juin 1994;17(3):236-42.

Pustejovsky JE, Rodgers MA. Testing for funnel plot asymmetry of standardized mean differences. Res Syn Meth. mars 2019;10(1):57-71.

Rücker G, Nikolakopoulou A, Papakonstantinou T, Salanti G, Riley RD, Schwarzer G. The statistical importance of a study for a network meta-analysis estimate. BMC Med Res Methodol. déc 2020;20(1):190.

Rücker G, Schwarzer G. Reduce dimension or reduce weights? Comparing two approaches to multi-arm studies in network meta-analysis. Statist Med. 10 nov 2014;33(25):4353-69.

Shams-Alizadeh N, Maroufi A, Asadi Z, Rahmani K, Hassanzadeh K. Trazodone as an Alternative Treatment for Neuroleptic-Associated Akathisia: A Placebo-Controlled, Double-Blind, Clinical Trial. J Clin Psychopharmacol. 2020;40(6):611-4.

Shim SR, Kim SJ, Lee J, Rücker G. Network meta-analysis: application and practice using R software. Epidemiol Health. 8 avr 2019;41:e2019013.

Sterne JAC, Savović J, Page MJ, Elbers RG, Blencowe NS, Boutron I, et al. RoB 2: a revised tool for assessing risk of bias in randomised trials. BMJ. 28 août 2019;l4898.

Stryjer R, Rosenzcwaig S, Bar F, Ulman AM, Weizman A, Spivak B. Trazodone for the Treatment of Neuroleptic-Induced Acute Akathisia: A Placebo-Controlled, Double-Blind, Crossover Study. Clinical Neuropharmacology. sept 2010;33(5):219-22.

Thompson SG, Sharp SJ. Explaining heterogeneity in meta-analysis: a comparison of methods. Statist Med. 30 oct 1999;18(20):2693-708.

Weiden PJ, Mann JJ, Haas G, Mattson M, Frances A. Clinical nonrecognition of neuroleptic-induced movement disorders: a cautionary study. Am J Psychiatry. sept 1987;144(9):1148-53.

© 2024 Gerolymos C et al. *JAMA Network Open.*
