# Supplementary material for: Drug Efficacy in the Treatment of Antipsychotic-Induced Akathisia: A Systematic Review and Network Meta-Analysis
Source: JAMA Netw Open. 2024 Mar 7;7(3):e241527. doi: 10.1001/jamanetworkopen.2024.1527 (PMC10921255; doi:10.1001/jamanetworkopen.2024.1527)
Supplement: Supplement 2. — Data Sharing Statement [file jamanetwopen-e241527-s002.pdf]

Data Sharing Statement

Gerolymos. Drug Efficacy in the Treatment of Akathisia. *JAMA Netw Open*. Published March 07, 2024. doi:10.1001/jamanetworkopen.2024.1527

Data
Data available: Yes
Data types: Data (not involving human participants)
How to access data: guillaume.fond@ap-hm.fr
When available: With publication

Supporting Documents
Document types: None

Additional Information
Who can access the data: researchers whose proposed use of the data has been approved
Types of analyses: for a specified purpose
Mechanisms of data availability: with investigator support with a signed data access agreement).
